# Extracellular vesicle storm during the course of Ebola virus infection in primates

**DOI:** 10.3389/fcimb.2023.1275277

**Published:** 2023-11-15

**Authors:** Andrea Vucetic, Andrea Lafleur, Marceline Côté, Darwyn Kobasa, Mable Chan, Fernando Alvarez, Ciriaco Piccirillo, George Dong, Martin Olivier

**Affiliations:** ^1^ Department of Microbiology and Immunology, McGill University, Montréal, QC, Canada; ^2^ Infectious Diseases and Immunity in Global Health Program, Research Institute of the McGill University Health Centre, Montréal, QC, Canada; ^3^ Department of Biochemistry, Microbiology and Immunology and Centre for Infection, Immunity and Inflammation, University of Ottawa, Ottawa, ON, Canada; ^4^ Special Pathogen Program, National Microbiology Laboratory, Public Health Agency of Canada, Winnipeg, MB, Canada; ^5^ Department of Medical Microbiology and Infectious Diseases, University of Manitoba, Winnipeg, MB, Canada; ^6^ Federation of Clinical Immunology (FOCiS) Centres of Excellence in Translational Immunology (CETI), Research Institute of the McGill University Health Centre, Montréal, QC, Canada

**Keywords:** extracellular vesicle, exosome, Ebola virus, inflammatory response, inflammation, Ebola virus disease, primates

## Abstract

**Introduction:**

Ebola virus (EBOV) is an RNA virus of the Filoviridae family that is responsible for outbreaks of hemorrhagic fevers in primates with a lethality rate as high as 90%. EBOV primarily targets host macrophages leading to cell activation and systemic cytokine storm, and fatal infection is associated with an inhibited interferon response, and lymphopenia. The EBOV surface glycoprotein (GP) has been shown to directly induce T cell depletion and can be secreted outside the virion via extracellular vesicles (EVs), though most studies are limited to epithelial cells and underlying mechanisms remain poorly elucidated.

**Methods:**

To assess the role of GP on EBOV-induced dysregulation of host immunity, we first utilized EBOV virus-like particles (VLPs) expressing VP40 and NP either alone (Bald-VLP) or in conjunction with GP (VLP-GP) to investigate early inflammatory responses in THP-1 macrophages and in a murine model. We then sought to decipher the role of non-classical inflammatory mediators such as EVs over the course of EBOV infection in two EBOV-infected rhesus macaques by isolating and characterizing circulatory EVs throughout disease progression using size exclusion chromatography, nanoparticle tracking-analysis, and LC-MS/MS.

**Results:**

While all VLPs could induce inflammatory mediators and recruit small peritoneal macrophages, pro-inflammatory cytokine and chemokine gene expression was exacerbated by the presence of GP. Further, quantification of EVs isolated from infected rhesus macaques revealed that the concentration of vesicles peaked in circulation at the terminal stage, at which time EBOV GP could be detected in host-derived exosomes. Moreover, comparative proteomics conducted across EV populations isolated from serum at various time points before and after infection revealed differences in host-derived protein content that were most significantly pronounced at the endpoint of infection, including significant expression of mediators of TLR4 signaling.

**Discussion:**

These results suggest a dynamic role for EVs in the modification of disease states in the context of EBOV. Overall, our work highlights the importance of viral factors, such as the GP, and host derived EVs in the inflammatory cascade and pathogenesis of EBOV, which can be collectively further exploited for novel antiviral development.

## Introduction

Ebola virus (*Zaire ebolavirus*; EBOV) is an enveloped negative-sense RNA virus of the *Filoviridae* family that is responsible for severe hemorrhagic fevers and acute systemic disease, known as Ebola virus disease (EVD), with mortality rates than can reach 90% in both humans and non-human primates (NHPs) (Feldmann and Geisbert, 2011; Bennett et al., 2017). Ebola viruses are endemic to regions of West and Equatorial Africa and have significant epidemic potential, as was demonstrated by the 2013-2016 West African outbreak, which incurred over 28,000 cases and approximately 11,000 fatalities reported in the three most affected countries: Guinea, Liberia, and Sierra Leone (Coltart et al., 2017). EVD is considered to be an emerging zoonotic disease, with fruit bats of the *Pteropodidae* family likely acting as the natural reservoir of the virus (Groseth et al., 2007). In humans, EVD first manifests as non-specific flu-like symptoms followed by a cytokine storm and severe fluid loss, after which it can evolve into fatal complications such as hemorrhage, systemic capillary leak, septic shock, and multi-organ failure leading to death (Hunt et al., 2015; Leligdowicz et al., 2016). Despite concerted research efforts into understanding the pathology of EBOV, there is still a substantial need for comprehensive prevention measures, better surveillance in high-risk areas associated with urbanization, and innovations on prophylaxes and treatments based on further elucidation of mechanisms underlying this host-pathogen interaction (Malvy et al., 2019).

The single-stranded RNA genome of EBOV is comprised of a linear array of seven genes encoding eight viral proteins. Beginning at the 3’ end, these proteins include the nucleoprotein (NP), VP (viral protein) 35, VP40, two major forms of glycoprotein (GP; transmembrane and secreted), VP30, VP24, and the viral RNA-dependent RNA polymerase L (Feldmann et al., 1996; Messaoudi et al., 2015). NP directly encapsidates the viral genome and, in association with the structural elements VP35 and VP24, viral RDRP L and the transcription factor VP30, forms a ribonucleoprotein complex that comprises the helical nucleocapsid (Wan et al., 2017; Yuki Takamatsu, 2018). NP’s interaction with the viral genome occurs through a C-terminal α-helix and is non-specific (Wan et al., 2017; Yuki Takamatsu, 2018). The matrix protein VP40 is responsible for recruiting the nucleocapsid and regulating viral budding from the host plasma membrane (Noda et al., 2002; Noda et al., 2006; Bharat et al., 2012). When expressed either alone or in conjunction with other EBOV proteins, VP40 can assemble and form virus-like particles (VLPs) that are morphologically identical to native viruses. Finally, the surface GP is expressed on the lipid envelope of the virus and mediates viral entry into host cells through the fusion of viral and cellular membranes following cathepsin cleavage, which exposes the receptor binding domain, culminating in binding to the viral receptor, Niemann-Pick C1 (Chandran et al., 2005; Lee and Saphire, 2009; Carette et al., 2011; Cote et al., 2011; Miller et al., 2012; Rhein and Maury, 2015).

Macrophages and dendritic cells are the initial targets of EBOV infection and replication, although the cellular tropism of the virus can be quite broad, especially as it progressively spreads throughout the host organism (Schnittler and Feldmann, 1998; Geisbert et al., 2003; Wong et al., 2014). Virulence first ensues as the virus abrogates the host innate antiviral response through the inhibition of interferon production and signaling, which allows for dissemination and unrestricted replication in infected cells (Cardenas et al., 2006; Reid et al., 2006; Misasi and Sullivan Nancy, 2014). Extensive infection leads to the alteration of early cellular gene expression in activated macrophages, followed by sustained release of pro-inflammatory cytokines and chemokines, including TNF-α, MCP-1, IL-1β, IL-6, IL-8, MIP-1α, and MIP-1β, along with reactive oxygen and nitrogen radicals (Gupta et al., 2001; Stroher et al., 2001; Wahl-Jensen et al., 2011; Wong et al., 2014). This cytokine storm is pivotal for the pathogenesis of EBOV, as it creates a positive feedback loop whereby pro-inflammatory mediators attract new target cells and recruit other inflammatory cells such as neutrophils and eosinophils, collectively contributing to coagulopathy and increased vascular permeability (Fisher-Hoch et al., 1985; Baize et al., 2002; Malvy et al., 2019). Pathology is further associated with diminished T cell activation and proliferation, along with lymphopenia – defined as a massive loss of peripheral CD4^+^ and CD8^+^ T lymphocytes – shown previously in studies characterizing the course of EVD in fatal human and experimentally infected NHP cases (Geisbert et al., 2000; Reed et al., 2004; Wauquier et al., 2010). This severe loss of lymphocytes is a hallmark of EVD, impairing the host’s ability to fight viral infection and recover (Geisbert et al., 2000). While the exact mechanisms driving lymphopenia remain elusive, abortive infection of lymphocytes by EBOV, whereby T cells infected with EBOV undergo autophagy, likely contributes to EBOV-mediated T cell depletion (Younan et al., 2018; Younan et al., 2019).

Extracellular vesicles (EVs) are a critical and generally understudied class of biological players that have been shown to contribute to the pathology of many diseases (Pluchino and Smith, 2019). EVs encompass a heterogeneous group of cell-derived membranous vesicles that include apoptotic bodies, microvesicles (ectosomes), and exosomes – the latter of which is specifically defined as nanovesicles of endosomal origin that are 30-150 nm in size (Johnstone et al., 1987; Raposo and Stoorvogel, 2013; Pluchino and Smith, 2019). EVs are ubiquitously released by almost all cell types, and contain biologically active cargo, including proteins, lipids, and nucleic acids, thus playing a key role in intercellular communication and the transmission of disease states through means such as the modulation of immunological responses (Théry et al., 2002; Anderson et al., 2016; Edgar, 2016). Exosomes have a particularly well-established role in the potentiation of viral infection, whereby viruses such as the human immunodeficiency virus (HIV), Epstein-Barr virus (EBV), hepatitis c virus (HCV) and, more recently, severe acute respiratory syndrome coronavirus 2 (SARS-CoV2) have all been reported to manipulate host endosomal machinery for delivery of viral nucleic acids and proteins to diverse target cells (Saad MH et al., 2021). The viruses, which lack functional machinery required for EV biogenesis, hijack host functions to package viral mediators within exosomes, providing protection of viral cargo from degradation in the extracellular environment, maintaining low immunogenicity, and facilitating uptake by target cells which, in turn, will be modulated to favor infection (Saad MH et al., 2021).

In the context of EBOV, recent studies in NHPs and guinea pig models have indicated unconventional secretion of viral matrix, nucleoproteins, and glycoproteins, suggesting packaging of viral proteins by infected cells into EVs via the exosomal pathway (Reynard et al., 2011; Pleet et al., 2019). The unconventional trafficking of these proteins outside the virion highlights the importance of these proteins in viral immunopathogenesis, given various EBOV proteins play diverse roles throughout disease progression. *In vitro* studies have elucidated novel functions for the matrix protein, suggesting that exosomes released from VP40-transfected cells carry VP40 as cargo, are enriched in cytokines such as IL-15, TGF-β1, and IFN-γ, and are capable of inducing cell death in recipient T cells and monocytes while potentially promoting growth and division of epithelial cells (Pleet et al., 2016; Pleet et al., 2017; Cantoni and Rossman, 2018; Pleet et al., 2018). EVs containing NP, on the other hand, are thought to be mostly immunoregulatory, dampening dose-dependent type I IFN responses by recipient cells (Pleet et al., 2019). Together, these EBOV proteins are thought to cooperate to modulate host cell EV biogenesis – VP40 by increasing the rate of cell cycling and, in turn, amplifying EV output, and NP likely by trafficking viral and host RNAs into EVs (Pleet et al., 2019). The understanding of the role of GP in this process, is thus far limited to its role in virion uptake, despite its identification within circulatory EVs and wide use in immunizations due to its strong immunogenicity (Lai et al., 2017). Furthermore, non-traditional mediators of inflammation such as EVs and exosomes are known to have the ability to activate interacting cells and exacerbate pathology in a number of various diseases, and yet have not been extensively studied in the context of EVD (Olivier and Fernandez-Prada, 2019). To address these knowledge gaps, we aimed to decipher the host-pathogen interaction from the perspective of both classical and unconventional mediators of inflammation using *in vitro* and *in vivo* models, and by investigating EV-mediated inflammatory events in NHPs.

Herein, we hypothesized that the recruitment and activation of inflammatory cells and production of pro-inflammatory cytokines and chemokines was GP-dependent, and that EBOV infection progression in an NHP model would correlate with an increase in circulating EVs and inflammatory mediators collectively contributing to disease pathogenesis. Together, these diverse models provide a more complete picture of the host-pathogen interaction and highlight the synergistic role of both classical and non-classical mediators of inflammation in EVD progression.

## Materials and methods

### Cell culture

293T cells were cultured in Dulbecco’s Modified Eagle Medium (DMEM) 1X (Wisent, St. Bruno, QC, Canada) supplemented with 10% fetal bovine serum (FBS; Wisent) and 1% penicillin-streptomycin-glutamine (PSG; Wisent) at 37°C and 5% CO_2_ for 8-10 days prior to use for transfection.

Immortalized murine macrophage B10R cells (derived from the bone marrow of B10A.Bcg^r^ congenic mouse strain) were cultured in DMEM 1X supplemented with 10% FBS and 1% PSG at 37°C and 5% CO_2_ for 8-10 days before use (Radzioch et al., 1991). 5.0 × 10^5^ cells/well were plated in 6-well plates and incubated for 24 hours prior to the experiment. Following overnight incubation, cells were washed with 1 mL of warm phosphate-buffered saline (PBS) and incubated for 1 hour in new media before stimulation under conditions specific to the experiment.

THP-1 (ATCC® TIB-202™) cells were cultured in RPMI-1640 1X medium (Wisent) supplemented with 10% FBS, 1% PSG, 1 mM sodium pyruvate (Millipore Sigma, Burlington, MA, USA), 4.5 g/L glucose (Millipore Sigma), and 50 μM of 2-β-mercaptoethanol (Millipore Sigma) at 37°C and 5% CO_2_ for 8-10 days prior to use. For differentiation to macrophages, 1.5 × 10^6^ cells/well were plated in 6-well plates and incubated with 50 ng/mL of phorbol 12-myristate 13-acetate (PMA; Millipore Sigma) in RPMI-1640 1X medium supplemented with 5% FBS and 1% PSG (Tsuchiya et al., 1982). After 24 hours, the PMA-containing media was replaced with fresh RPMI-1640 1X medium supplemented with 5% FBS and 1% PSG and the cells were incubated for 48 hours. On the day of the experiment, the cells were washed with 1mL of warm PBS and incubated for 1 hour in Minimum Essential Medium (MEM; Gibco, Grand Island, NY, USA) supplemented with 5% FBS and 1% PSG prior to stimulation under conditions specific to the experiment.

### EBOV VLP production and purification

EBOV VLPs were produced as described previously (Stewart et al., 2021). Briefly, 293T cells were seeded in 10 cm^2^ dishes and incubated overnight in DMEM supplemented with 10% FBS and 1% PSG at 37°C and 5% CO_2_ to achieve target confluency of 60-80%. Cells were co-transfected using jetPRIME® (Polyplus Transfection, Illkirch, France) with 3 μg of VP40 fused to β-lactamase (VP40-BlaM), 3 μg of EBOV NP (both plasmids were kind gifts of Dr. Lijun Rong, University of Chicago), and 4 μg of plasmid encoding full length EBOV GP (kind gift of James Cunningham, Brigham and Women’s Hospital) for the production of VLPs containing GP (VLP-GP). To produce Bald VLPs, cells were co-transfected with 5 μg of VP40-BlaM and 5 μg of EBOV NP expressing plasmids. Culture media were replaced 24 hours the next day and culture supernatants were harvested at 48-, 72-, and 96-hours post-transfection.

Culture supernatants were centrifuged at 240 × *g* (RCF_avg_) for 5 minutes to remove cell debris. Supernatants were then transferred into 17 mL thin-wall polypropylene tubes (Beckman Coulter, Brea, CA, USA) and 2 mL of 20% sucrose (Millipore Sigma) prepared in PBS and filter-sterilized was added to each tube and topped up with PBS. Tubes were centrifuged at 56,000 × *g* (RCF_avg_) for 1 hour and 30 minutes at 4°C in an SW 32.1 Ti swinging bucket rotor (Beckman Coulter). The supernatant was aspirated, and the pellet was re-suspended in 100 μL of PBS, parafilmed, and placed at 4°C overnight. VLP concentrations were measured with the Micro BCA^™^ Protein Assay Kit (Thermo Fisher Scientific, Waltham, MA, USA).

### Animal ethics and biosafety

All mouse experiments were carried out in pathogen-free conditions and in accordance with the regulations of the Canadian Council of Animal Care Guidelines and Institutional Animal Care and Use Committees at McGill University under ethics protocol number 7791. Mice were euthanized after 6 hours using isoflurane (3%) and CO_2_ asphyxiation followed by cervical dislocation.

Experiments with rhesus macaques were carried out at the BSL-4 National Microbiology Laboratory in Winnipeg, Manitoba. Macaques were housed in adjoining individual primate cages allowing social interactions, under controlled conditions of humidity, temperature, and light (12-h light/dark cycles). Food and water were freely available, and animals were monitored at least twice daily and provided with additional enrichment by trained personnel. In accordance with the regulations of the Canadian Council of Animal Care Guidelines, animal procedures were carried out under anesthesia using IM Ketamine (10 mg/kg) and isoflurane inhalation (5%), supplemented with oxygen. All treatments were administered by trained personnel under the supervision of veterinary staff, with important efforts made to promote animal welfare and to minimize the suffering of the animals. Humane endpoint criteria, specified and approved by the Institutional Animal Care and Use Committee, were applied to determine when animals should be humanely euthanized. Human euthanasia was performed by trained personnel under the supervision of veterinary staff using intracardiac injection of pentobarbital sodium (100 mg/kg).

### Mouse experiments

Female BALB/c mice (6-8 weeks old) purchased from Charles River Laboratories (Wilmington, MA, USA) were injected intraperitoneally (i.p.) with 100 μg of VLP-GP or Bald VLP diluted with PBS to a final volume of 250 μL, or 250 μL of PBS. IP injection was utilized to ascertain the inflammatory response to acute infection. Mice were euthanized after 6 hours using isoflurane and CO_2_ asphyxiation followed by cervical dislocation. Immune cells from the peritoneal cavity were collected by lavage with 5 mL of ice-cold PBS. Total cell counts of the lavage fluid were made using a hemocytometer. An aliquot of the cell suspension was stained for analysis by flow cytometry. The remaining cell suspension was centrifuged at 365 × *g* (RCF_avg_) for 10 minutes, the supernatant was removed but retained for cytokine/chemokine array analysis, and the pellet was lysed with TRIzol^™^ reagent (Thermo Fisher Scientific) for qRT-PCR.

### Quantitative RT-PCR

Time-course experiments were performed with B10R macrophage cells that were left untreated, stimulated with 100 ng/mL LPS (Millipore Sigma) for 1 hour, or stimulated with 5 μg/mL Bald VLP for 30 minutes, 1, 2, and 4 hours. Dose-response experiments were performed with B10R macrophage cells that were left untreated, stimulated with 100 ng/mL LPS, or stimulated with 1, 3, or 5 μg/mL VLP-GP or Bald VLP for 4 hours. Human cell experiments were performed with PMA-differentiated THP-1 cells that were left untreated, stimulated with 100 ng/mL LPS, or stimulated with 5 μg/mL VLP-GP or Bald VLP for 6 hours. Mouse experiments were analyzed using cells collected from i.p. lavage of mice injected for 6 hours with 100 μg of VLP-GP or Bald VLP, or PBS, as previously described.

Following stimulation, cells were washed with warm PBS (*in vitro* only) and lysed with TRIzol™ reagent (Thermo Fisher Scientific) according to the manufacturer’s protocol. Clearance of possible genomic DNA contamination was performed using RQ1 RNAse-free DNAse (Promega, Madison, WI, USA) according to the manufacturer’s protocol. 1 μg of total RNA was used to perform cDNA first-strand synthesis using the ProtoScript® II Reverse Transcriptase (New England BioLabs, Ipswich, MA, USA), random primers (Invitrogen, Carlsbad, CA, USA), and deoxynucleotides (New England BioLabs). Samples were then treated with *Escherichia coli* Rnase H (New England BioLabs) for clearance of RNA-DNA helices. Standardized amounts of cDNA were mixed with custom-designed murine/human primers (Integrated DNA Technologies, Coralville, IA, USA) and SYBR Green Supermix (Bio-Rad, Hercules, CA, USA) to perform qRT-PCR using the CFX96 Touch Real-Time PCR Detection System (Bio-Rad). Results were analyzed by ▵▵Ct method with the CFX-managing software (Bio-Rad) using 18S (B10R/mouse experiments) and GAPDH (THP-1 experiments) as housekeeping genes.

### Flow cytometry

Single-cell suspensions collected from mouse peritoneal cavity lavages were stained with the following fluorescence-conjugated mAbs: α-CD3-BUV737 (17A2) (BD Biosciences, Franklin Lakes, NJ, USA), α-CD4-FITC (GK1.5) (BD Biosciences), α-CD8-V500 (53-6.7) (BD Biosciences), α-CD11b-e450 (M1/70) (Invitrogen), α-CD11c-PerCP-Cy™ 5.5(HL3) (BD Biosciences), α-CD19-APC (1D3) (Invitrogen), α-CD49b-PE (DX5) (BD Biosciences), α-F4/80-PECy7 (BM8) (Invitrogen), and α-Ly6G-Alexa700 (BioLegend, San Diego, CA, USA). Non-viable cells were excluded using fixable viability dye eFluor780 or 506 reagents (Thermo Fisher Scientific). Data were collected using a FACS Fortessa X-20 flow cytometer (BD Biosciences), and results were analyzed using FlowJo version 9 software (TreeStar, Ashland, OR, USA).

### Rhesus macaque infections

On day 0, two rhesus macaques (NHP 2441 and NHP 2401) (*Macaca mulatta)* were infected by intramuscular route with 1000 TCID_50_ of Ebola virus Makona C07 diluted in DMEM (Wisent) at a dose volume of 2 mL (1 mL at each site, two sites) and monitored post-challenge for clinical signs of disease (Wong et al., 2016). Intramuscular infection was utilized to follow the course of infection, in contrast to IP, which provides a snapshot of acute infection. Animals were sampled on day 0 (pre-infection bleed), days 3-6 post-infection, as well as on the date of mandatory euthanasia (day 7). On these dates, a blood specimen was collected for serum analysis and quantification of viremia. Oral, nasal, and rectal swab specimens were collected to quantify levels of virus shedding. Viral genome copies were measured using qRT-PCR, and infectious viral titer was quantified by TCID_50_ assay from samples collected on day 0 (pre-infection) and days 4, 6, and 7 post-infection. Serum was isolated from blood samples and irradiated with 5 MegaRads for inactivation prior to being shipped to the BSL-2 laboratory at the Research Institute of the McGill University Health Centre (RI-MUHC; Montreal, QC, Canada).

### Multiplex cytokine/chemokine quantification assay

Supernatant from mouse i.p. lavage fluid was analyzed by bead-based mouse multiplex assay for 44 different cytokines, chemokines, and growth factors (MD44), while serum from rhesus macaques was analyzed by bead-based human multiplex assay for 42 different cytokines, chemokines, and growth factors (HD42; Eve Technologies, Calgary, AB, Canada). Collectively, these include 6Ckine, epidermal growth factor (EGF), erythropoietin, Eotaxin-1, fibroblast growth factor (FGF)-2, FMS-like tyrosine kinase 3 ligand (Flt-3L), Fractalkine, granulocyte-colony stimulating factor (G-CSF), granulocyte-macrophage colony-stimulating factor (GM-CSF), growth-regulated oncogene (GRO)-α, interferon (IFN)-α2, IFN-γ, IFN-β1, interleukin (IL)-1α, IL-1β, IL-1RA, IL-2, IL-3, IL-4, IL-5, IL-6, IL-7, IL-8, IL-9, IL-10, IL-11, IL-12 (p40), IL-12 (p70), IL-13, IL-15, IL-16, IL-17A, IL-18, IL-20, interferon γ-induced protein (IP)-10, keratinocyte chemoattractant (KC), leukemia inhibitory factor (LIF), lipopolysaccharide-induced CXC chemokine (LIX), monocyte chemoattractant protein (MCP)-1, MCP-3, MCP-5, monocyte colony-stimulating factor (M-CSF), macrophage-derived chemokine (MDC), monokine induced by interferon gamma (MIG), macrophage-inflammatory protein (MIP)-1α, MIP-1β, MIP-2, MIP-3α, MIP-3β, platelet-derived growth factor (PDGF)-AA, PDGF-AB/BB, regulated on activation normal T cell expressed and secreted (RANTES), sCD40L, thymus and activation regulated chemokine (TARC), transforming growth factor (TGF)-α, tissue inhibitor of metalloproteinase (TIMP)-1, tumor necrosis factor (TNF)-α, TNF-β, and vascular endothelial growth factor (VEGF)-A. Multiplex data was visualized using a cytokine/chemokine heat map that was generated using Heatmapper (Babicki et al., 2016).

### Size exclusion chromatography column

Sepharose CL-4B (20 mL; GE Healthcare, Uppsala, Sweden) was poured into a 10 mL polypropylene column (Thermo Fisher Scientific) and the slurry liquid was allowed to drain through the bottom to permit the packing of beads. The column was then equilibrated by washing with ~100 mL of 100 mM ammonium acetate (0.22 μm filtered; Millipore Sigma).

### Purification of extracellular vesicles from serum of EBOV-infected rhesus macaques

Serum (~250 μL) samples collected from each animal on day 0 (pre-infection), and days 3, 4, 5, 6, and 7 post-infection with EBOV were treated by exposure to 5Mrad gamma radiation to destroy viral infectivity. They were then diluted with an equal volume of 200 mM ammonium acetate. Diluted samples were then loaded onto the column, followed by elution with 100 mM ammonium acetate (0.22 μm filtered). The eluate was collected in 2 sequential fractions of 1 mL (waste) followed by 12 sequential fractions of 500 μL. For each fraction, the number of particles was determined by nanoparticle tracking analysis (NTA) and protein concentrations were dosed with the Micro BCA^™^ Protein Assay Kit. Of each fraction, ~200 μL was stored at -80°C for subsequent proteomic analysis and transmission electron microscopy (TEM) on thawed fractions. Whenever fractions were pooled together, protein content in the samples was re-dosed.

### Nanoparticle tracking analysis

NTA measurements of isolated EVs were performed with the NanoSight NS500 (Malvern Panalytical, Malvern, Worcestershire, UK) in the laboratory of Dr. Janusz Rak (RI-MUHC). Fractions were diluted with PBS before injection in the sample chamber. For the determination of particle sizes and numbers, 3 sequential videos of 30 seconds were acquired and analyzed to provide the mean, mode, median, and estimated concentration for each particle size (Filipe et al., 2010; Oosthuyzen et al., 2013). Instrument settings were optimized and kept constant between corresponding samples.

### Transmission electron microscopy

Bald VLPs and VLP-GPs were diluted with PBS, and EVs were diluted with 100 mM ammonium acetate to a concentration of 100 ng/μL. Samples were directly placed on Fomvar Carbon grids (Mecalab, Montreal, QC, Canada), fixed with 1% glutaraldehyde in 0.1 M sodium cacodylate buffer for 1 minute, washed 3 times with autoclaved Milli-Q® water for 1 minute each, and stained with 1% uranyl acetate for 1 minute. Samples were visualized using FEI Technai-12 120 kV transmission electron microscope and AMT XR80C CCD Camera (Facility for Electron Microscopy Research, McGill University, Montreal, QC, Canada).

### Liquid chromatography-MS/MS

Liquid chromatography tandem mass spectrometry (LC-MS/MS) was performed at the Institut de Recherches Cliniques de Montréal (Université de Montréal, Montreal, QC, Canada). Proteins (5 μg) derived from purified serum EVs were precipitated with 15% trichloroacetic acid/acetone and processed for LC-MS/MS analysis. After precipitation, in-solution digestion was performed with trypsin at a ratio of 1:25 protease/protein. After overnight incubation at 37°C, the reactions were quenched by the addition of formic acid to a final concentration of 0.2% and cleaned with C18 Zip Tip pipette tips (Millipore Sigma), before MS analysis. Extracted peptides were injected into a Zorbax Extended-C18 desalting column (Agilent, Santa Clara, CA, USA) and subsequently chromatographically separated on a Biobasic 18 Integrafit capillary column (Thermo Fisher Scientific) on a Nano high-performance liquid chromatography system (1100 series unit; Agilent). Eluted peptides were electrosprayed as they exited the capillary column and were analyzed on a QTRAP 4000 linear ion trap mass spectrometer (SCIEX, Framingham, MA, USA).

### Protein database search

Individual sample tandem mass spectrometry spectra were peak listed using the Distiller version 2.1.0.0 software (http://www.matrixscience.com/distiller.html) with peak picking parameters set at 1 for signal-noise ratio and 0.3 for correlation threshold (Atayde et al., 2019). The peak-listed data were then searched against the NCBI database with the Mascot software version 2.3.0.523 (Matrix Science, Boston, MA, USA). Mascot was set up to search the Refseq (txid9544; 68,028 proteins) and Uniprot (45,199 proteins) *Macaca mulatta* database with a fragment ion mass tolerance of 0.020 Da and a parent ion tolerance of 10.0 PPM. Carbamidomethyl of cysteine was specified as a fixed modification and oxidation of methionine residues was specified as a variable modification in the search engine. Scaffold software version 4.8.9 (Proteome Software Inc., Portland, OR, USA) was used to validate MS/MS peptide and protein identifications. Peptide identifications were accepted if they could be established at greater than 95.0% probability by the Peptide Prophet algorithm with Scaffold delta-mass correction (Keller et al., 2002). Protein identifications were accepted if they could be established at greater than 95.0% probability and contained at least 2 identified peptides (Keller et al., 2002). Proteins that contained similar peptides and could not be differentiated using MS/MS analysis alone were grouped to satisfy the principles of parsimony. The proteins sharing significant peptide evidence were grouped into clusters. The final number of peptides per protein was represented by the average of the biological replicates after normalization to the total number of peptides.

### Bioinformatics analysis

Normalization, quantification, and comparisons of proteins among serum-derived EV samples from NHP 2401 and 2441 were performed using the Scaffold software. Visualizations of set intersections in a matrix layout were generated by UpSetR (Lex et al., 2014). Gene Ontology comparisons were performed via Panther (www.pantherdb.org) (Mi et al., 2018). Pathway analysis was performed by mapping protein sets to human orthologues using Blast2GO (www.blast2go.com), followed by Gene Set Enrichment Analysis (GSEA) using Reactome Pathway Database (www.reactome.org) (Ana Conesa et al., 2005; Marc Gillespie et al., 2021).

### Statistical analysis

For all experiments, all data points are shown, along with the mean and standard error of the mean (SEM) or standard deviation (SD), unless otherwise stated. Differences between groups were tested using a one-way or repeated measures ANOVA with Tukey’s or Dunnett’s *post-hoc* test for multiple comparisons. For single comparisons, statistical analyses were performed using the one-tailed unpaired Student’s *t*-test with Welch’s correction. *P*-values are represented on each figure as follows: *p < 0.05, **p < 0.01, ***p < 0.001, ****p < 0.0001. Data and statistical analyses were performed using GraphPad Prism (version 8; GraphPad Software, San Diego, CA, USA).

## Results

### EBOV VLPs display the characteristic filamentous morphology of EBOV

**Figure 1:**
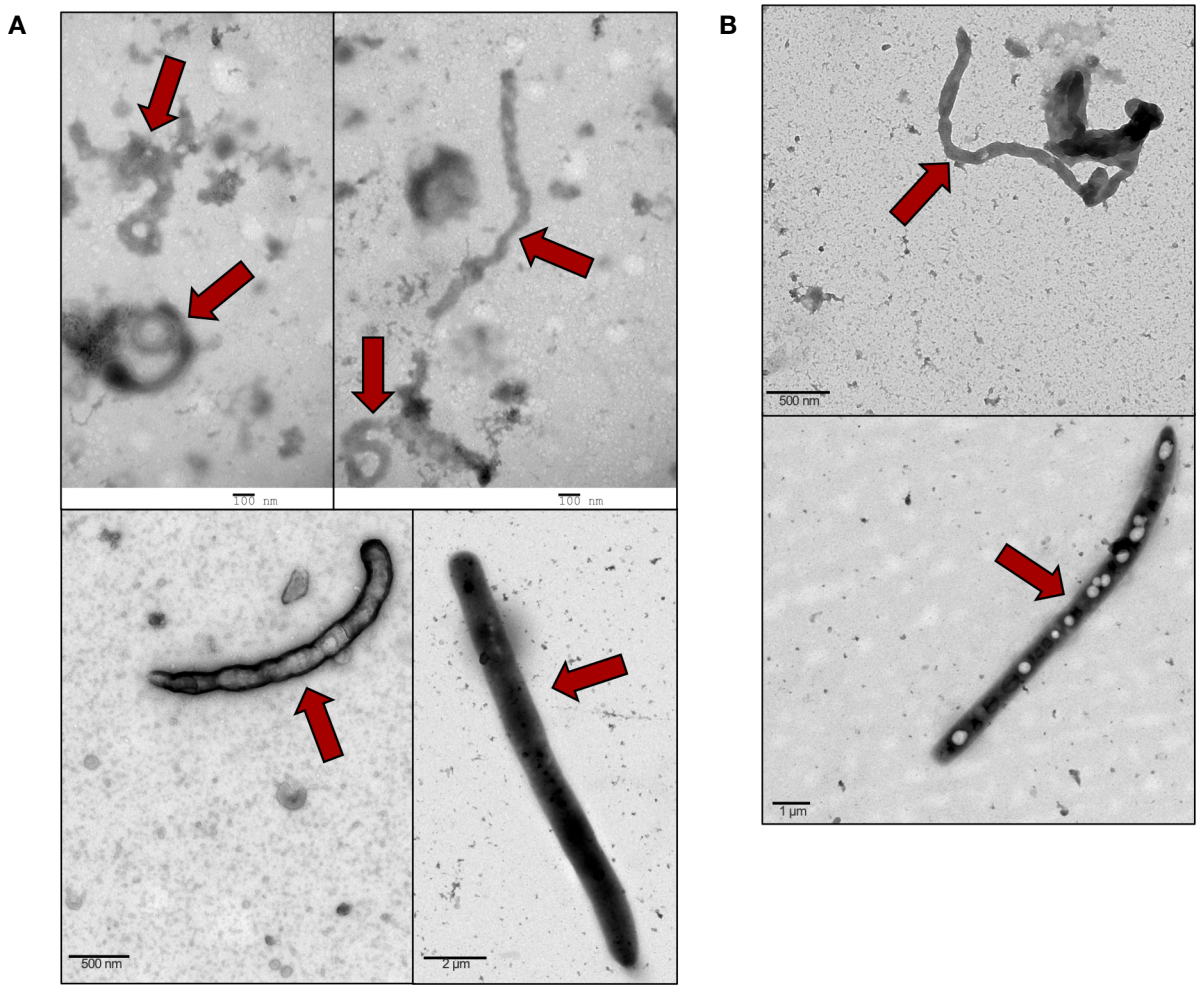
Purified VLPs display the characteristic filamentous morphology of EBOV. VLPs were purified by sucrose cushion ultracentrifugation of transfected cell supernatants. VLPs (red arrows) were prepared for TEM by negative staining with uranyl acetate to reveal the ultrastructure. **(A)** VLP-GPs were generated by co-transfection of 293T cells with VP40-BlaM, NP, and full-length Zaire GP with the mucin region. TEM magnifications: 18,500x (top panels), 9,300x (bottom left panel), 1,900x (bottom right panel). **(B)** Bald VLPs were generated by co-transfection of 293T cells with VP40-BlaM and NP, alone. TEM magnifications: 9,300x (top panel), 2,900x (bottom panel).

VLP-GPs and Bald VLPs were produced by co-transfecting 293T cells with VP40-BlaM, NP, and *Zaire ebolavirus* GP, or only VP40-BlaM and NP, respectively, then purified on a sucrose cushion by ultracentrifugation. TEM analysis of produced VLP-GPs (**Figure 1A**) and Bald VLPs (**Figure 1B**) confirmed the presence of particles in the preparation that are morphologically identical to native filoviruses (Noda et al., 2002). These VLPs were subsequently used to stimulate murine macrophages (*in vitro*), human THP-1 monocyte-derived macrophages (*in vitro*), and mouse peritoneal cavity cells (*in vivo*) to model and investigate the inflammatory response induced in host cells upon interaction with the virus.

### EBOV GP exacerbates VLP-induced pro-inflammatory response in human macrophages

In order to investigate the mechanisms underlying the induction of pro-inflammatory mediators in human macrophages, for which there is limited data in the context of EBOV, we first performed time-course and dose-response experiments in murine-derived B10R macrophages to determine the best conditions for maximal activation of host cells and release of cytokines and chemokines of interest (Stroher et al., 2001; Wahl-Jensen et al., 2011). These data revealed optimal stimulation conditions of macrophages with 5 μg/mL of VLPs for 4 hours or longer for appropriate downstream activation (**Figure S1**).

**Figure 2:**
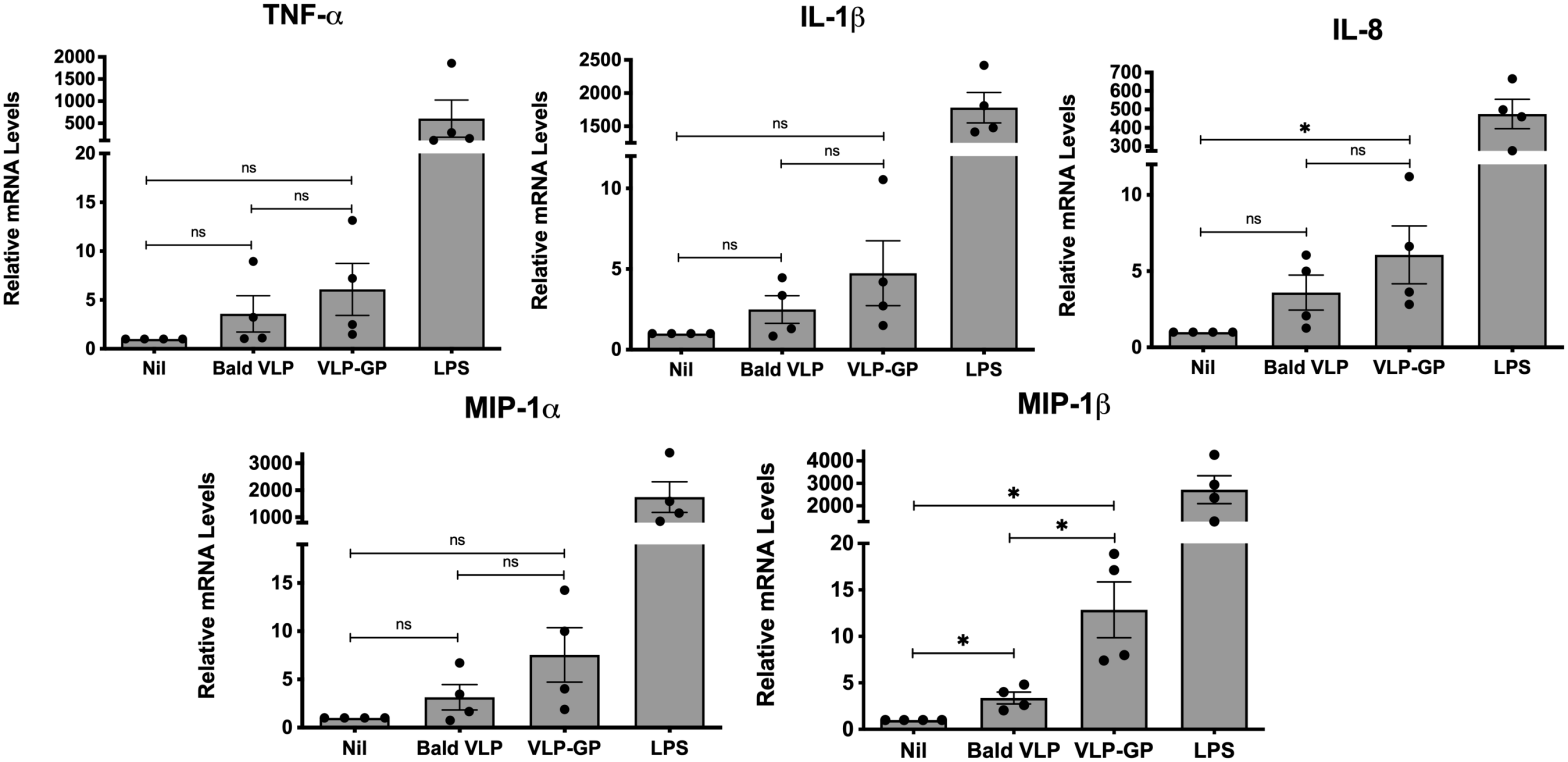
EBOV GP exacerbates induction of inflammatory gene expression by VLPs *in vitro* in PMA-differentiated THP-1 cells. Pro-inflammatory cytokine and chemokine mRNA levels in PMA-differentiated THP-1 cells following stimulation with Bald VLP (5 µg/mL), VLP-GP (5 µg/mL), or LPS (100 ng/mL) for 6 hours, as measured by qRT-PCR. The unstimulated state (Nil) is shown for reference. GAPDH was used as a normalization gene. Data are shown as mean ± SEM and each point represents one experiment, performed in duplicate (*n* = 4, one-tailed unpaired t-test with Welch’s correction, *p < 0.05; ns, not significant).

We next sought to explore the inflammatory responses induced by EBOV in human macrophages using our predetermined conditions. Given evidence of TLR4-mediated of induction of pro-inflammatory chemokines and cytokines by GP, we chose the THP-1 cell line for further experiments (Okumura et al., 2010; Escudero-Pérez et al., 2014; Lai et al., 2017). This macrophage cell line expresses high levels of TLR4 and NOD1/NOD2, thus providing significant insight into the immunomodulatory role of molecules and macromolecular structures (Chanput et al., 2014; Jakopin and Corsini, 2019). THP-1 monocytes were differentiated into macrophages with PMA and then left unstimulated (Nil; basal expression of 1) or stimulated for 6 hours with 5 μg/mL of Bald VLP, VLP-GP, or LPS (100 ng/mL) as a positive control. Six hours was chosen as the stimulation period in this experiment to further increase the window of time for the modulation of genes for pro-inflammatory mediators. After 6 hours, gene expression of all pro-inflammatory cytokines and chemokines was induced upon stimulation with Bald VLP or VLP-GP compared to the unstimulated group, as measured by qRT-PCR (**Figure 2**). Specifically, expression of chemokines IL-8 (murine MIP-2; ~6-fold) and MIP-1β (~13-fold) was significantly upregulated in cells stimulated with VLP-GP relative to the unstimulated group (*p* < 0.05; one-tailed unpaired t-test with Welch’s correction), while expression of TNF-α (~6-fold; *p* = 0.0765), IL-1β (~5-fold; *p* = 0.0801), and MIP-1α (~8-fold; *p* = 0.0516) followed similar trends. While all measured cytokine and chemokine genes were more induced in cells stimulated with VLP-GP compared to Bald VLP, expression of MIP-1β significantly increased by approximately 4-fold in the presence of the GP (*p* < 0.05). Collectively, we observed that although both VLPs were immunogenic in human macrophages after stimulation for 6 hours, mediators induced by VLP-GP consistently followed a trend indicating a greater inflammatory response, though only significant for MIP-1β. These data suggest an exacerbating role for the GP in promoting inflammation during the early stages of host-EBOV interaction, at which time macrophages and DCs are the initial targets of infection and the sites of replication (Schnittler and Feldmann, 1998; Geisbert et al., 2003; Wong et al., 2014).

### Interaction with EBOV VLPs alters mouse peritoneal immune cell population profiles

**Figure 3:**
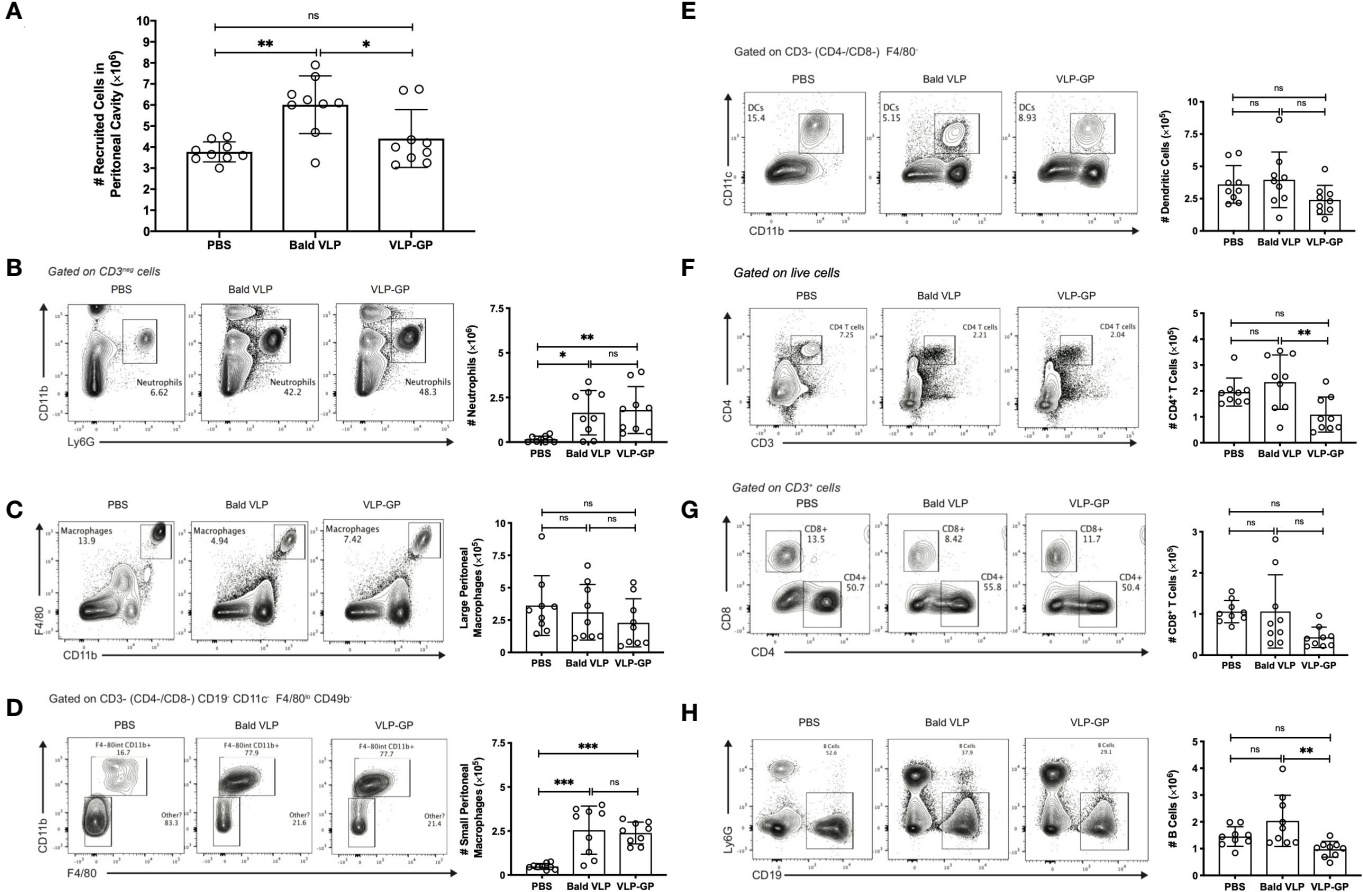
Interaction with EBOV VLPs changes immune cell population profile in mouse peritoneal cavity compared to PBS control. **(A)** Number of cells collected from peritoneal cavity lavage (5 mL) following 6-hour stimulation with PBS, Bald VLP (100 µg), or VLP-GP (100 µg), as measured with hemocytometer. Bars represent mean ± SD of three separate experiments and each point represents one mouse (*n* = 9 mice per group, one-way ANOVA with Tukey’s correction for multiple comparisons, *p < 0.05; **p < 0.01). **(B-H)** Representative flow cytometry plots of gating strategies and corresponding peritoneal cavity cell counts (5 mL) of **(B)** neutrophils (lymphoid^-^, CD11c^-^, CD11b^+^, Ly6G^+^), **(C)** large peritoneal macrophages (lymphoid^-^, CD11c^-^, F4/80^+^, CD11b^+^), **(D)** small peritoneal macrophages (lymphoid^-^, CD11c^-^, F4/80^lo^, CD11b^+^), **(E)** dendritic cells (lymphoid^-^, F4/80^-^, CD11c^+^, CD11b^+^), **(F)** CD4^+^ T cells (myeloid^-^, CD3^+^, CD4^+^), **(G)** CD8^+^ T cells (myeloid^-^, CD3^+^, CD8^+^), and **(H)** B cells (myeloid^-^, CD3^-^, CD19^+^). Bars represent mean ± SD of three separate experiments and each point represents one mouse (*n* = 9 mice per group, one-way ANOVA with Tukey’s correction for multiple comparisons, *p < 0.05; **p < 0.01; ***p < 0.001; ns, not significant).

Following our *in vitro* observations of VLP-GP-mediated production of inflammatory cytokines and chemokines, we sought to investigate the effect of our VLPs on the inflammatory response *in vivo*. The mouse peritoneal cavity is a membrane-bound and fluid-filled abdominal compartment, in which multiple immune cell types reside and are easily recruited due to direct proximity to lymphatic vessels (Ghosn et al., 2010; Ray and Dittel, 2010; Zimmerman, 2018). Importantly, increases in immune cell numbers occur in response to antigenic stimulation in the peritoneal space over a short period of time. For example, injection of LPS has been shown to enhance peritoneal cavity inflammation through recruitment and activation of various inflammatory cells at early timepoints post-intraperitoneal injection (1-6 hours) (Miyazaki et al., 2004; Adhikari et al., 2017; Zimmerman, 2018). To further characterize the inflammatory response observed *in vitro*, we therefore studied total and specific immune cell recruitment to the peritoneal cavity of BALB/c mice, along with simultaneous changes in gene expression and production of pro-inflammatory mediators after challenge with PBS, Bald VLP, or VLP-GP for 6 hours – a timepoint that was determined based on prior studies investigating LPS and induction of the inflammatory response by other pathogens, notably the protozoan parasite *Leishmania* (Yang et al., 2014; Adhikari et al., 2017; Charlebois et al., 2023). We initially found that the Bald VLP recruited a significantly higher number of total cells to the peritoneal cavity of mice compared to both PBS (*p* < 0.01) and VLP-GP (*p* < 0.05; one-way ANOVA with Tukey’s correction for multiple comparisons) (**Figure 3A**). To explain the immunological dynamics during this 6-hour time lapse and the observed differences between groups, we measured changes in quantity and phenotype of peritoneal immune cells by flow cytometry. From this, we first observed that both Bald VLP (*p* < 0.05) and VLP-GP (*p* < 0.01) induced significant recruitment of neutrophils (lymphoid^-^, CD11c^-^, CD11b^+^, Ly6G^+^) into the mouse peritoneal space, compared to PBS (**Figure 3B**). We then observed that both Bald VLP and VLP-GP caused recruitment of small (SPM; lymphoid^-^, CD11c^-^, F4/80^lo^, CD11b^+^) but not large peritoneal macrophages (LPM; lymphoid^-^, CD11c^-^, F4/80^+^, CD11b^+^) (**Figures 3C, D**) (Ghosn et al., 2010). Our initial probing of macrophage populations in the peritoneum following challenge with VLPs was centered on LPMs, which make up approximately 90% of the peritoneal macrophages in unstimulated animals and express high levels of established macrophage surface markers CD11b and F4/80 (Ghosn et al., 2010). However, when we unexpectedly saw no differences across all three groups, we decided to investigate other populations of cells that represented an intermediate, to investigate whether another cell population was arising. Using the available panel, we were able to segregate LPMs (blue) and SPMs (red), the latter of which express much lower levels of CD11b and F4/80, derive from blood monocytes that rapidly infiltrate the peritoneal cavity in response to inflammatory stimuli, and become the predominant population in the peritoneal cavity after differentiation (**Figure S2A**) (Ghosn et al., 2010). Given the inflammatory nature of the VLPs, our observation that Bald VLP (*p* < 0.001) and VLP-GP (*p* < 0.001) significantly increased the number of SPMs in the peritoneal space after 6 hours, comparatively to PBS, is logical. We did not see any significant differences in the number of DCs (lymphoid^-^, F4/80^-^, CD11c^+^, CD11b^+^) present in the mouse peritoneal cavity across all three experimental groups (**Figure 3E**). When further dissecting lymphocyte populations, no significant decreases in cell counts were apparent between either VLP-GP or Bald VLP and the control group. While more apparent differences were expected considering lymphopenia is a well-established consequence of EBOV infection, previous work has only reported significant GP-mediated T lymphocyte death 4 days post-exposure to GP (Iampietro et al., 2017). Thus, the slight decrease in the number of CD4^+^ (myeloid^-^, CD3^+^, CD4^+^) and CD8^+^ (myeloid^-^, CD3^+^, CD8^+^) T cells in the peritoneal space when mice were challenged with VLP-GP for 6 hours, compared to both Bald VLP (*p* < 0.01; *p* = 0.0617) and PBS (*p* = 0.0668; *p* = 0.0636), may indicate the beginning of this process (**Figures 3F, G**). Further, injection with VLP-GP led to a lower number of B lymphocytes (myeloid^-^, CD3^-^, CD19^+^) in the murine peritoneal space after 6 hours, as compared to Bald VLP (*p* < 0.01) challenge, but not the control group (**Figure 3H**). Altogether, while no striking decrease in lymphocytes was observed in response to VLP-GP as compared to the PBS group, these data highlight alterations to the peritoneal immune profile at early timepoints post-infection. Further, differences between Bald VLP and VLP-GP groups indicate a potential role for GP in this process. Finally, we confirmed that other cells (negative for all stains) present in the mouse peritoneal cavity fluid did not display differences between the experimental groups (**Figure S2B**).

**Figure 4:**
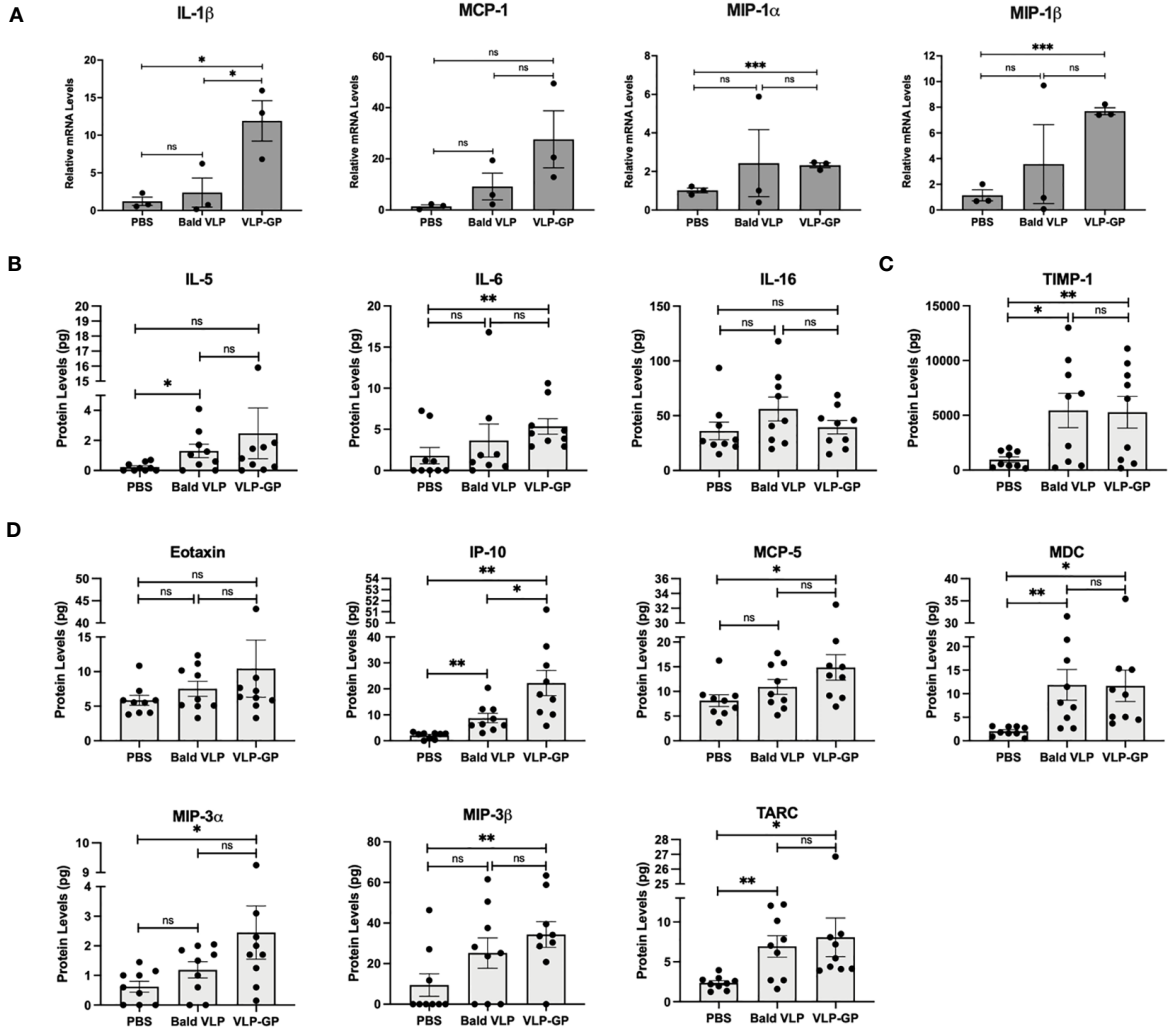
Interaction with EBOV VLPs alters inflammatory gene expression and protein production in mouse peritoneal cells compared to PBS control. **(A)** Pro-inflammatory cytokine and chemokine mRNA levels in peritoneal cells following i.p stimulation with PBS, Bald VLP (100 µg), or VLP-GP (100 µg) for 6 hours, as measured by qRT-PCR. 18S rRNA was used as a normalization gene. Bars represent mean ± SEM of one experiment and each point represents one mouse (*n* = 3 mice per group, one-tailed unpaired *t*-test with Welch’s correction, *p < 0.05; ***p < 0.001). **(B)** Cytokine, **(C)** soluble factor TIMP-1, and **(D)** chemokine levels (pg) in 5 mL of peritoneal lavage fluid following i.p stimulation with PBS, Bald VLP (100 µg), or VLP-GP (100 µg) for 6 hours, measured by multiplex cytokine/chemokine array. Bars represent mean ± SEM of three separate experiments and each point represents one mouse (*n* = 9 mice per group, one-tailed unpaired *t*-test with Welch’s correction, *p < 0.05; **p < 0.01; ns, not significant).

Alongside these early changes in immune cell population profiles after challenge with VLPs, we also demonstrated that gene expression of several pro-inflammatory cytokines and chemokines was induced in the mouse peritoneal space in a manner exacerbated by the GP using qRT-PCR (**Figure 4A**). Specifically, gene expression of IL-1β (~12-fold; *p* < 0.05), MIP-1α (~2-fold; *p* < 0.001), and MIP-1β (~7-fold; *p* < 0.001) was significantly upregulated in cells of the peritoneal space of mice challenged with VLP-GP, compared to the PBS group (one-tailed unpaired *t*-test with Welch’s correction). Expression of MCP-1 (~20-fold; *p* = 0.0712) strongly followed this same trend but was not significant. Exceptionally, IL-1β expression was also significantly increased by approximately 5-fold in the peritoneal cells of mice challenged with VLP-GP compared to the Bald VLP group (*p* < 0.05), while expression of MCP-1 (~3-fold) followed this same trend. No remarkable differences were noted in the expression of MIP-1α between VLP-GP and Bald VLP groups. Altogether, these data are suggestive of a mechanism of inflammatory transcriptional activation that is initiated by the VLP and enhanced by the presence of GP.

Lastly, we evaluated the effects of intraperitoneal stimulation with VLPs on the production of cytokines, chemokines, and other mediators by immune cells after 6 hours, using a multiplex cytokine/chemokine array to quantify levels in lavage supernatants (**Figures 4B-D**). Production of T cell-derived cytokine IL-5 was significantly increased after challenge with Bald VLP (~1.5-fold; *p* < 0.05) relative to that of the PBS group, while a similar trend was seen after challenge with VLP-GP (~2-fold), although not statistically significant (one-tailed unpaired *t*-test with Welch’s correction) (**Figure 4B**). In mice injected with VLP-GP, we observed significantly higher levels of IL-6 compared to PBS-treated mice (~3-fold; *p* < 0.01), along with approximately 1.5-fold higher levels compared to the Bald VLP group, although not significant (**Figure 4B**). Production of IL-16, on the other hand, was slightly increased in the peritoneal cavity of mice injected with Bald VLP relative to both PBS (~1.6-fold; *p* = 0.0805) and VLP-GP (~1.4-fold; **Figure 4B**). Levels of inflammatory biomarker TIMP-1 were approximately 6-fold higher in the peritoneal cavity of mice challenged with both Bald VLP (*p* < 0.05) and VLP-GP (*p* < 0.01), compared to the PBS group (**Figure 4C**). Previous functional genomics studies performed with the spleens of BALB/c mice infected intraperitoneally with EBOV identified that TIMP-1 was upregulated in lethal infections only and is associated with leukocyte extravasation signaling (Cilloniz et al., 2011). There were no significant differences noted between levels of eotaxin, the eosinophil chemoattract, in the peritoneal cavity of mice across the three experimental groups (**Figure 4D**). Furthermore, quantification of chemokine IP-10 (CXCL10) revealed significantly higher protein levels in mice challenged with VLP-GP relative to the Bald VLP (~3-fold; *p* < 0.05) and PBS (~11-fold; *p* < 0.01), along with ~4-fold higher production in mice injected with Bald VLP compared to the PBS group (*p* < 0.01) (**Figure 4D**). Protein levels of MDC (CCL22) and TARC (CCL17) were significantly higher (~4-fold and ~2-fold, respectively) in mice that were challenged with Bald VLP (*p* < 0.01) or VLP-GP (*p* < 0.05), compared to PBS-injected mice (**Figure 4D**). No significant differences in the levels of these chemokines in the peritoneal space were observed between Bald VLP and VLP-GP groups. Finally, MCP-5 (CCL12; *p* < 0.05), MIP-3α (CCL20; *p* < 0.05), and MIP-3β (CCL19; *p* < 0.01) levels were found to be significantly increased (~1.5, 3, and 4-fold, respectively) in the mice injected with VLP-GP, compared to the PBS group (**Figure 4D**). Although Bald VLP administration increased levels of MCP-5 (~1.3-fold), MIP-3α (~2-fold), and MIP-3β (~3-fold) relative to that of the mice treated with PBS, the extent of induction did not reach statistical significance (*p* = 0.0833, 0.0542, 0.0561; respectively). There were no significant differences in the quantities of these chemokines in the peritoneal cavity between mice challenged with Bald VLP or VLP-GP. MCP-5 is a chemokine that has only been described in mice and is a structural and functional homologue of human MCP-1 (Sarafi et al., 1997). In fact, it has not yet been reported in the context of EBOV, and thus represents a novel marker of EBOV-induced inflammation in future studies performed in mouse models. Intriguingly, a similar study in which 100 μg of EBOV GP was administered i.p. in BALB/c mice showed that levels of IL-5, IL-6, MCP-1, and MIP-1β, among others, did not increase in the serum of the animals at 6 hours after treatment, relative to that measured pre-administration; significant differences were instead only observed when serum samples were assessed at time points greater than 6 hours (Lai et al., 2017). Collectively, our data has demonstrated clear changes in immune cell population profiles, along with alterations in pro-inflammatory cytokine and chemokine expression and production in response to interactions with VLPs, which are often exacerbated by the presence of the GP. Overall, this supports the findings from our studies *in vitro*, corroborates previous work on the crucial role of GP in viral pathogenesis, and offers new insight into early immune responses at the cellular and soluble factor level upon host contact with EBOV *in vivo*.

### Changes in circulating immune mediators correlate with viraemia in an NHP model

To investigate a model more akin to human EVD and to elucidate the role of circulating immune mediators over the course of EBOV infection, we next utilized an NHP model. Two rhesus macaques (NHP 2401 and 2441) were challenged with 1000 TCID_50_ of *Zaire ebolavirus* Makona strain C07. Both animals were humanely euthanized 7 days after infection according to scoring of clinical progression. Viral RNA was measured in whole blood using qRT-PCR and viral titers were quantified by TCID_50_ assay. Viremia was detected 4 days post-infection and significantly increased as the infection progressed (**Figure S3**). The peak viremia levels for both macaques were detected at days 6-7 post-infection. Peak viremia levels ranged from 5.62 × 10^4^ TCID_50_/mL (NHP 2401) to 3.16 × 10^5^ TCID_50_/mL (NHP 2441), or 8.93 × 10^6^ genome copies/mL (NHP 2401) to 7.46 × 10^7^ genome copies/mL (NHP 2441). The average peak viremia level for both macaques was 1.86 × 10^5^ TCID_50_/mL and 4.17 × 10^7^ genome copies/mL.

**Figure 5:**
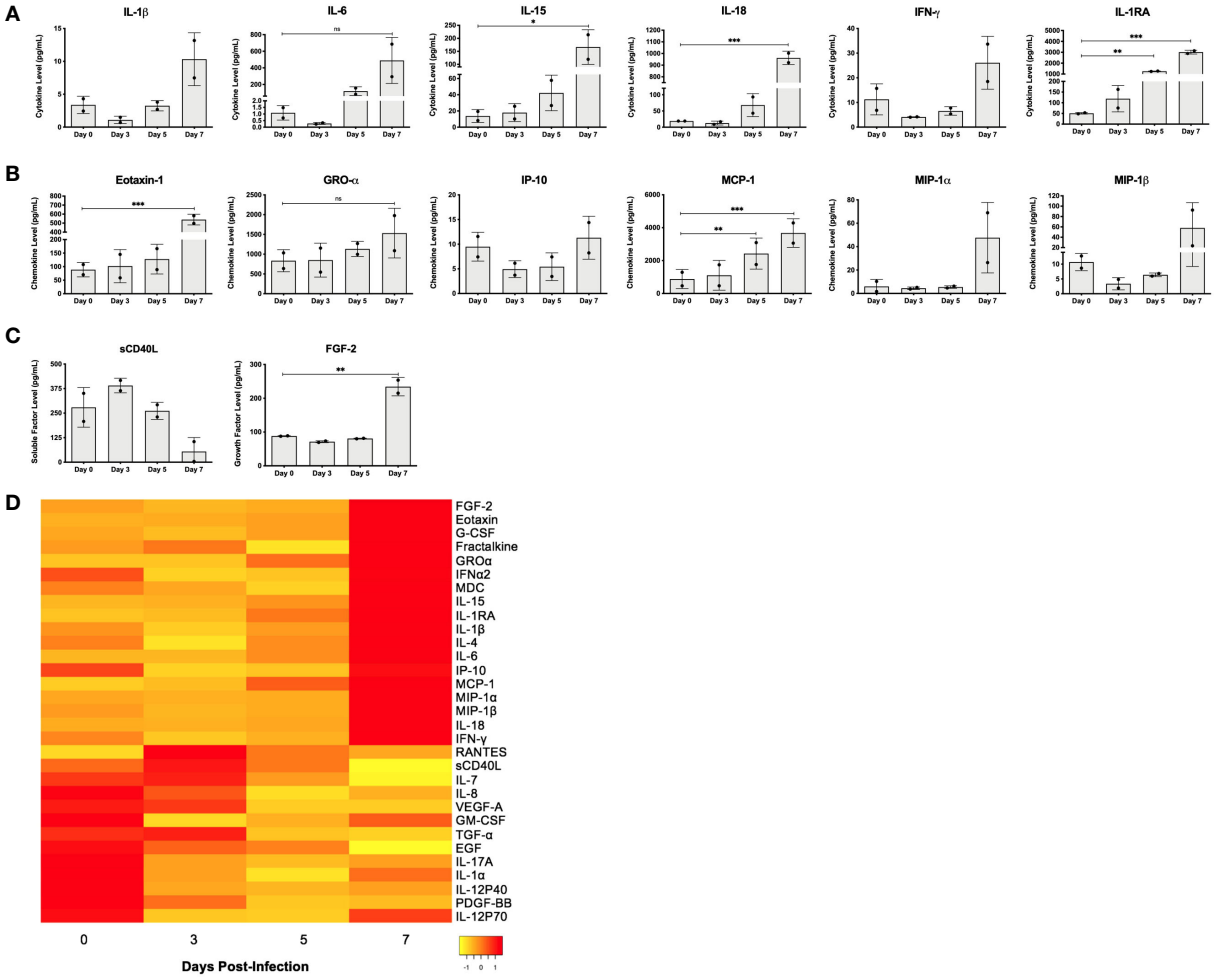
EBOV infection in rhesus macaques leads to differential production of cytokines, chemokines, and other factors throughout disease progression. **(A)** Cytokine, **(B)** chemokine, and **(C)** other soluble factor concentrations (pg/mL) in serum of rhesus macaques on day 0 (pre-infection), and days 3, 5 and 7 post-infection, as measured by multiplex cytokine/chemokine array. Bars represent mean ± SD (*n =* 2 macaques per time point, repeated measures one-way ANOVA with Dunnett’s correction for multiple comparisons, *p < 0.05; **p < 0.01; ***p < 0.001). Statistical significance is measured against day 0. **(D)** Heat map representation of serum cytokine, chemokine, and other factor levels from samples collected on day 0 (pre-infection), and days 3, 5, and 7 post-infection, as measured by multiplex cytokine/chemokine array (*n* = 2 macaques per time point, biological replicates averaged for heat map expression). Concentrations are indicated using a color scale that ranges from yellow (low) through orange to red (high). NS, Non-significant.

Levels of circulating cytokines, chemokines, and soluble factors present in gamma irradiated serum samples (5Mrads) collected from NHPs on day 0 (pre-infection) and days 3, 5, and 7 days after infection with EBOV were measured by multiplex cytokine/chemokine array (**Figure 5**). Notably, we detected significantly increased levels of inflammatory cytokines IL-15 (~12-fold; *p* < 0.05) and IL-18 (~51-fold; *p* < 0.001), and regulatory cytokine IL-1 receptor antagonist (IL-1RA; ~60-fold; *p* < 0.001) at 7 days post-infection as compared to day 0. IL-6 convincingly replicated this trend at the same time points (~449-fold; *p* = 0.0720; repeated measures one-way ANOVA with Dunnett’s correction for multiple comparisons) (**Figure 5A**). Furthermore, although not statistically significant, we observed higher levels of IL-1β (~ 3-fold) and IFN-γ (~2-fold) at the terminal stage of infection (day 7), relative to pre-infection levels (**Figure 5A**). Chemokines eotaxin-1 (~6-fold; *p* < 0.001) and MCP-1 (~4-fold; *p* < 0.001) were detected at levels that were significantly higher on day 7 as compared to measures before infection, while GRO-α (~2-fold; *p* = 0.0738) followed a similar trend (**Figure 5B**). Again, while not statistically significant, we observed higher levels of activated T cell chemoattractant IP-10 (~1.2 fold; CXCL10), and leukocyte attractants MIP-1α (~8-fold) and MIP-1β (~5-fold) 7 days post-infection, relative to conditions prior to infection (**Figure 5B**). Serum measurements of cytokine IL-1RA (~25-fold; *p* < 0.01) and chemokine MCP-1 (~3-fold; *p* < 0.01) also increased significantly between day 0 and day 5 post-infection (**Figures 5A, B**). Lastly, we observed a significant increase in fibroblast growth factor 2 (FGF-2; ~3-fold; *p* < 0.01) and a sharp decrease in soluble CD40-ligand (sCD40L; ~5-fold) 7 days post-infection, compared to day 0 (**Figure 5C**). Interestingly, we detected an initial decrease in serum concentrations of IL-1β, IL-6, IL-18, IFN-γ, IP-10, MIP-1α, MIP-1β, and FGF-2 from day 0 to day 3, followed by a progressive increase after day 3 (**Figures 5A-C**). These specific observations were also noted for plasma levels of IL-1β, IFN-γ, IL-6, and MIP-1α reported in a similar study in which cynomolgus macaques were challenged with 1000 PFU of EBOV Makona strain C07 and resultant immune responses were followed over time to provide insight into potential mechanisms of pathogenesis (Versteeg et al., 2017). The overarching pattern pertaining to the remainder tested including IL-15, eotaxin-1, GRO-α, MCP-1, and IL-1RA reflects a stepwise increase in serum concentration over time, from day 0 to day 7 post-infection (**Figures 5A, B**). Relative changes in serum levels of all tested markers over the time course of infection can be visualized by heat map (**Figure 5D**). Overall, changes in the levels of circulating immune mediators correlated with EVD progression in the macaques, though the number of NHPs utilized prohibited statistical significance.

### The release of EVs in circulation correlates with EVD progression in an NHP model

Following the investigation of soluble inflammatory factors over the course of EBOV infection in NHPs, we aimed to decipher the potential role of EBOV extracellular vesicles as non-classical inflammatory mediators. To this end, EVs were isolated by size-exclusion chromatography (SEC) from serum samples collected from the macaques at various time points-post infection, as detailed in the materials and methods section (Böing et al., 2014; Monguio-Tortajada et al., 2019). EV preparations were assessed for purity and composition using nanoparticle analysis, protein/EV ratios, and transmission electron microscopy (**Figures S4-S6**) (Filipe et al., 2010; Théry et al., 2018). By virtue of comprehensiveness, EVs derived from serum samples corresponding to day 0 (pre-infection), and days 3, 4, 5, 6, and 7 post-infection are referred to as D0, D3, D4, D5, D6, and D7 EVs, respectively.

**Figure 6:**
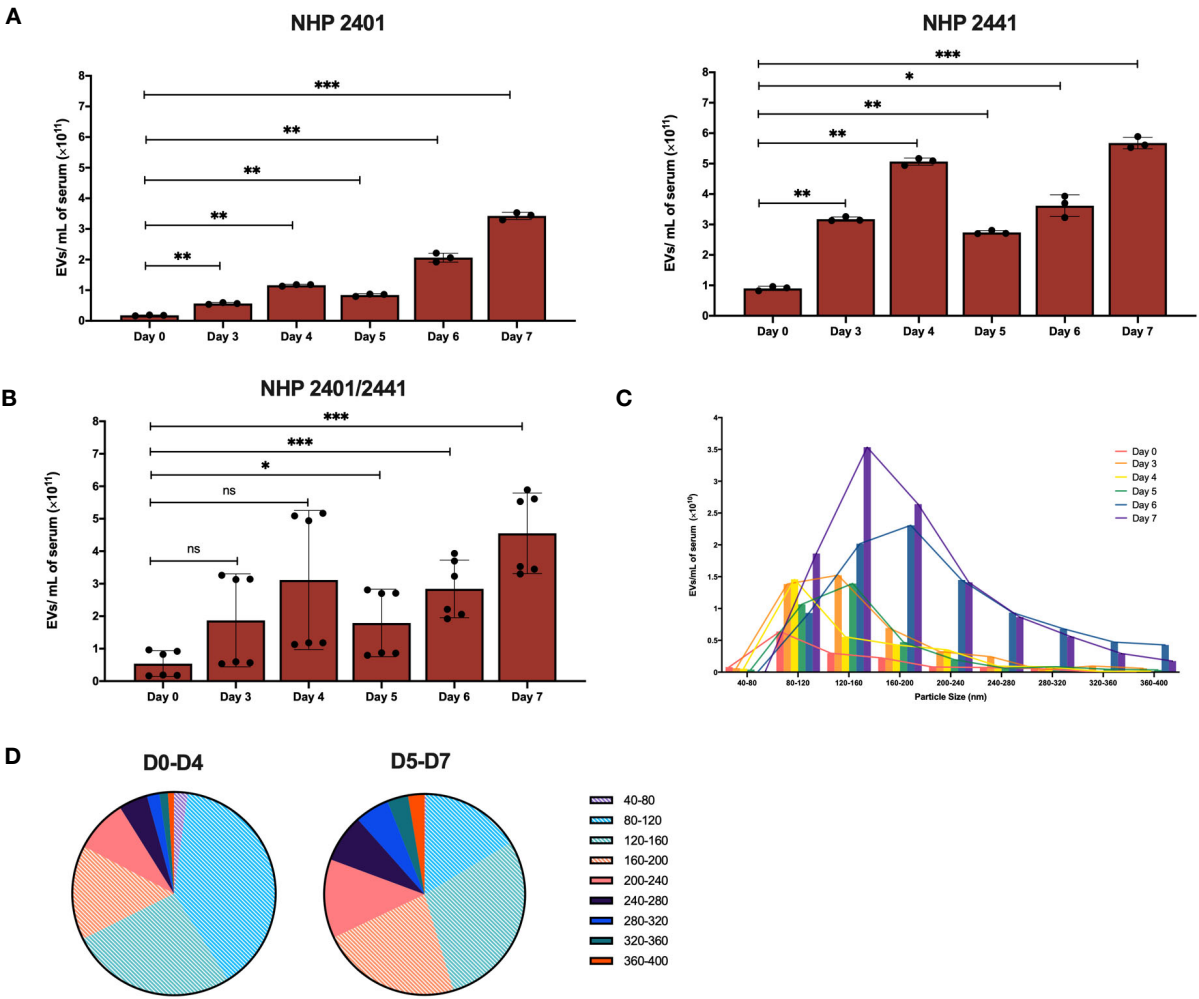
Concentration of EVs in circulation of EBOV-infected rhesus macaques peaks at the terminal stage of infection. **(A)** Concentration of EVs isolated by SEC from serum of NHP 2401 (left panel) and NHP 2441 (right panel), or **(B)** both. Samples collected on day 0 (pre-infection), and days 3, 4, 5, 6, and 7 post-infection were analyzed by nanoparticle tracking analysis of fractions 4-8. Bars represent mean ± SD of 3 separate NanoSight videos recorded per fraction (*n* = 2, repeated measures one-way ANOVA with Dunnett’s correction for multiple comparisons, *p < 0.05; **p < 0.01; ***p < 0.001). Statistical significance is measured against day 0. **(C)** Representative histogram of serum-derived EV size distributions (40-80 nm, 80-120 nm, 120-160 nm, 160-200 nm, 200-240 nm, 240-280 nm, 280-320 nm, 320-360 nm, 360-400 nm) according to respective concentrations in fraction 6 isolated by SEC from samples collected on day 0 (pre-infection) and days 3, 4, 5, 6, and 7 post-infection in NHP 2401 and 2441. Bars represent mean of isolations from two macaques per time point (*n* = 2). **(D)** Pie charts of size distributions of entire EV populations recovered in fraction 6 at early timepoints post-infection (D0-D4) and at late stages of EVD (D5-D7). Small EVs (< 200 nm) are indicated with a white hatching pattern. NS, Non-significant.

Following validation of the purity of EV preparations, we aimed to profile the dynamics of the EV populations (numbers and size) in circulation over the course of EBOV infection in the NHP model. Using the NTA measurements previously obtained for each EV-containing fraction, we were able to calculate the concentrations of serum-derived EVs (particles/mL of serum) present at each day pre- and post-infection (collective analysis of fractions 4-8) (**Figure S7**). In both EBOV-infected NHPs, the concentration of EVs was significantly higher (repeated measures one-way ANOVA with Dunnett’s correction for multiple comparisons) on all days after infection compared to D0 EV concentrations in both NHP 2401 (D3-D6: *p* < 0.01; D7: *p* < 0.001) and NHP 2441 (D3-D5: *p* < 0.01; D6: *p* < 0.05; D7: *p* < 0.001) (**Figure 6A**). Of note, the concentration of EVs in circulation peaked at the terminal stage of infection (**Figure 6A**). Interestingly, the concentration of EVs did not increase stepwise with each passing day post-infection, but rather dropped on D5 for NHP 2401 and on D5 and D6 for NHP 2441 (**Figure 6A**). In both NHPs, this decrease was followed by a sharp increase in the number of EVs on the final day of disease (7 days post-infection). This pattern may be reflective of changes occurring in the host during disease progression, a manifestation of alterations in the viral life cycle, or a combination of the two – though it is important to note that the number of macaques enables only the observation of a trend. Further studies are required to decipher the precise host-pathogen interaction responsible for this EV release trend. Furthermore, although there were variations in concentration values between both NHPs, the combined data displayed persistent and significant upwards trends relative to the pre-infection state (D3: *p* = 0.0819; D4: *p* = 0.0501; D5: *p* < 0.05; D6-D7: *p* < 0.001) (**Figure 6B**). With respect to size distribution, circulatory EVs between 80-160 nm were most abundant at earlier stages of infection (D0-D4), while EVs between 120-200nm were most abundant in later stages of infection, indicating a shift towards the production of slightly larger vesicles over the disease course (*n* = 2) (**Figures 6C, D**). Moreover, EVs with diameters in the ranges of 80-120 nm, 120-160 nm, and 160-200 nm, were most abundant on day 7 relative to other days post-infection (**Figures 6C, D**). Extended data corroborated that the purification process enabled complete recovery of EV populations and did not result in exclusion of any subtypes (**Figure S8**). Altogether, these data indicate that the number of circulatory EVs tends to increase over the course of EBOV infection, and that the EVs are larger later in EVD as compared to earlier timepoints post-infection.

### The proteomic content of extracellular vesicles in circulation is altered over the course of EBOV infection in NHP model

EVs are membrane-bound nanovesicles that are known to mirror the phenotype and function of the cell from which they are derived, which is signified through the presence of specific cargo such as proteins, lipids, metabolites, and nucleic acids (Monguio-Tortajada et al., 2019). To further dissect the consequences of EBOV infection in the NHP model, liquid chromatography tandem mass spectrometry (LC-MS/MS) was performed to compare the proteomic content of the serum-derived EVs from each time point before and after infection (pooled fractions 4-8) and attempt to identify their nature. Analysis of MS data against the UniProt Viruses database identified 4 distinct EBOV proteins present in circulating EVs at terminal stages of disease: GP, VP30, VP40 and NP (**Figure S9**, Data Sheet 1). Interestingly, no EBOV proteins were detected in the EVs prior to D6 post-infection, and very few spectra were identified for VP30, VP40 and NP. Further, while NP and VP30 were solely identified on D7 and in only one macaque, the viral protein VP30 was present in both macaques by the end of the course of infection. The presence of GP, however, was much more pronounced, with an average spectral count of 6.5 on D6 and of 25.5 on D7, indicating a rapid increase in its expression and trafficking in circulatory EVs towards the terminal phase of EVD.

**Figure 7:**
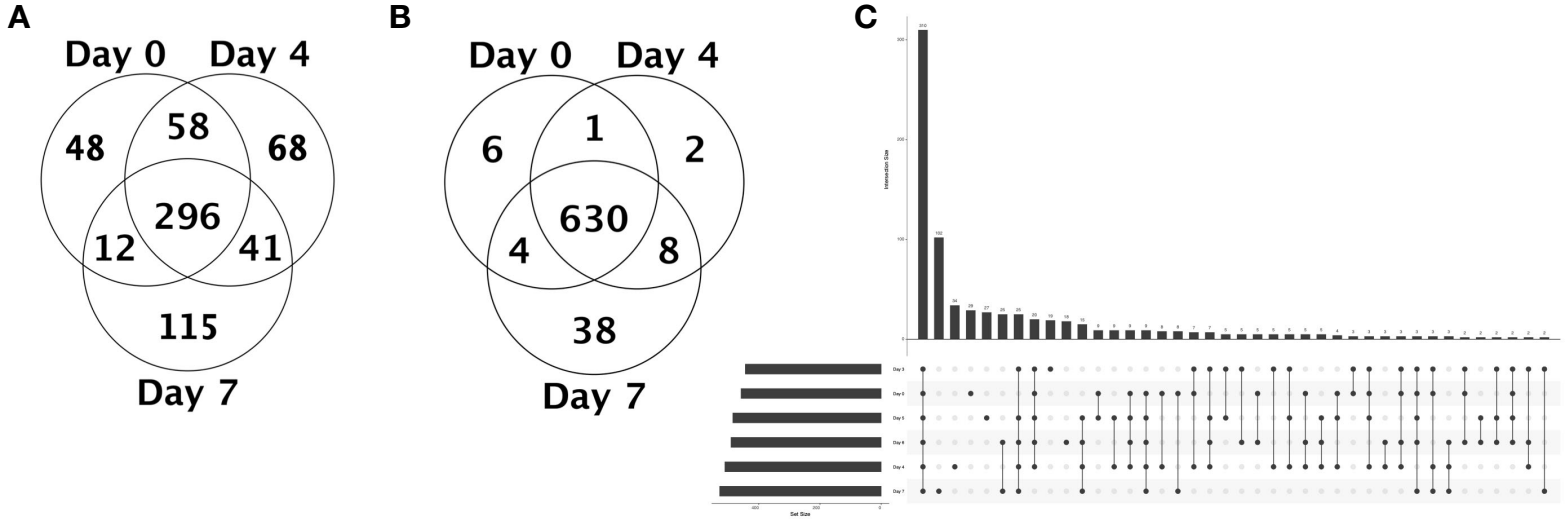
Unique and quantitative proteomic analysis of serum-derived EVs from EBOV-infected rhesus macaques before, during, and at the terminal stage of infection. **(A)** Venn diagram of proteins shared between day 0, 4, and 7 serum EVs, as well as unique proteins. If a protein is identified in only one sample among all samples within a category, the protein is considered to be present. **(B)** Venn diagram of proteins differentially enriched between day 0, 4, and 7 serum EVs, showing number of proteins with higher abundance in each category (day post-infection) compared to other categories. Venn diagram counts are based on a combination of statistical significance and quantitative profile. Proteins determined to be statistically insignificant are placed in the center region. Proteins determined to be statistically significant are placed in the segment with the best-fitting quantitative profile. (*n* = 2, ANOVA with Benjamini-Hochberg correction for multiple comparisons, *p* < 0.05). **(C)** UpSet plot for common and unique proteins across serum derived EVs isolated from blood samples collected pre- and post-infection. Each column corresponds to a set, and each row corresponds to one segment in a hypothetical six-set Venn diagram. Cells are either empty (light gray), indicating that this set is not part of that intersection, or filled (dark gray), showing that the set is participating in the intersection. Filled circles in the matrix (bottom) indicate the EV populations implicated in the respective intersection (above), or number of proteins shared between EVs from implicated time points pre- or post-infection (sets reflect an average of NHP 2401 and 2441). The total set size (number of proteins) of the EVs corresponding to the day pre-infection (day 0) or post-infection (days 3-7) is visualized in the bottom left. Vertical bars corresponding to a single filled circle in the matrix reflect the number of proteins unique to EVs from that given time point.

After corroborating that EBOV proteins – notably GP – were integrated into EVs, we sought to decipher the modulation of host-derived proteins in the exoproteome. Analysis of MS data against the RefSeq *Macaca mulatta* database revealed a total of 787 protein hits (689 individual proteins + 98 clusters) with a minimum average of 2 total spectrum counts between NHP 2401 and 2441 samples (S1 File). Strikingly, we observed considerable difference between the protein content of the EVs across all time points, but especially between D7 EVs relative to all others. To get a closer look at the physiological changes occurring during progressive EBOV infection at the level of EVs, we chose to first investigate unique and differentially enriched proteins between D0 (pre-infection), D4 (middle stage of infection), and D7 (time of death) EVs. We found that: 296 proteins were shared between D0, D4, and D7 EVs; 48 proteins were unique to D0 EVs; 68 proteins were unique to D4 EVs; 115 proteins were unique to D7 EVs (**Figure 7A**, **S7-9 Tables**). Among these uniquely expressed proteins, we found multiple proteins that are known to be enriched in exosomes or extracellular vesicles. For D0 EVs, these include tetraspanin CD9 antigen and multimerin-1, the latter of which was previously shown to be present in healthy human plasma-derived exosomes (**S7 Table**) (Caby et al., 2005; Looze et al., 2009; Kalra et al., 2012). For D4 EVs, these proteins include periostin and pleckstrin, both of which have been reported to be present in exosomes derived from human B cells infected with gamma herpesviruses, for example (**S8 Table**) (Kalra et al., 2012; Meckes et al., 2013). Amongst the 115 unique proteins identified in D7 EVs, some well-reported EV/exosome markers include heat shock protein HSP 90 kDa alpha (cytosolic)/beta, heat shock 70 kDa protein, heat shock cognate 71 kDa protein, MHC Class I protein, and integrin beta-2 precursor (**S9 Table**) (Kalra et al., 2012; Kowal et al., 2016). Collectively, these proteomic data confirm that the fraction preparations isolated from serum by SEC are composed of EVs and exosomes.

Moreover, looking at the proteins that displayed statistically significant enrichment at specific time points pre- or post-infection compared to the other two categories (D0 vs. D4 vs. D7), we observed that 6 proteins were more abundant in D0 EVs, 2 proteins were more abundant in D4 EVs, and 38 proteins were more abundant in D7 EVs, while 630 proteins were expressed at relatively similar levels (no significant differences; ANOVA with Benjamini-Hochberg correction for multiple comparisons) (**Figure 7B**, **S10 Table**). These data, along with the previous analysis of unique proteins, further demonstrate that EVs have the capacity to reflect distinct physiological changes occurring in the host and can contribute to disease pathogenesis in the context of EBOV infection.

We then utilized UpSet plot analysis to visualize intersecting protein sets across the EVs from all time points post-infection, which allowed us to identify both common and unique proteins in various combinations (Lex et al., 2014). Determinations of presence or absence of specific proteins were based on the average total spectrum counts from NHP 2401 and 2441. This analysis revealed 310 proteins that were common to EVs isolated from all time points post-EBOV infection (**Figure 7C**). Quantification of unique proteins revealed that D0, D3, D4, D5, D6 and D7 EVs contained 29, 19, 34, 27, 18, and 102 unique proteins, respectively (**Figure 7C**, **Tables S1-S6**). Interestingly, EVs from pre-infection (day 0), middle stage of infection (day 4), and time of death (day 7) were the populations with the highest relative number of unique proteins (29, 34, and 102, respectively), which may be reflective of broad changes in the physiology of the host at these phases in response to progressive infection (**Tables S1, S3, S6**). Unsurprisingly, when comparing serum derived EVs isolated from all pre- and post-infection time points, D7 EV populations contained a notably higher number of unique proteins (102 peptides), which was also observed in our earlier analysis of D0, D4, and D7 EVs (**Table S6**). In addition, the pair with the highest number of intersections were D6 and D7 EVs, which were released by the host during the later stages of disease and shared 25 proteins in common (**Figure 7C**, specific protein data not shown).

**Figure 8:**
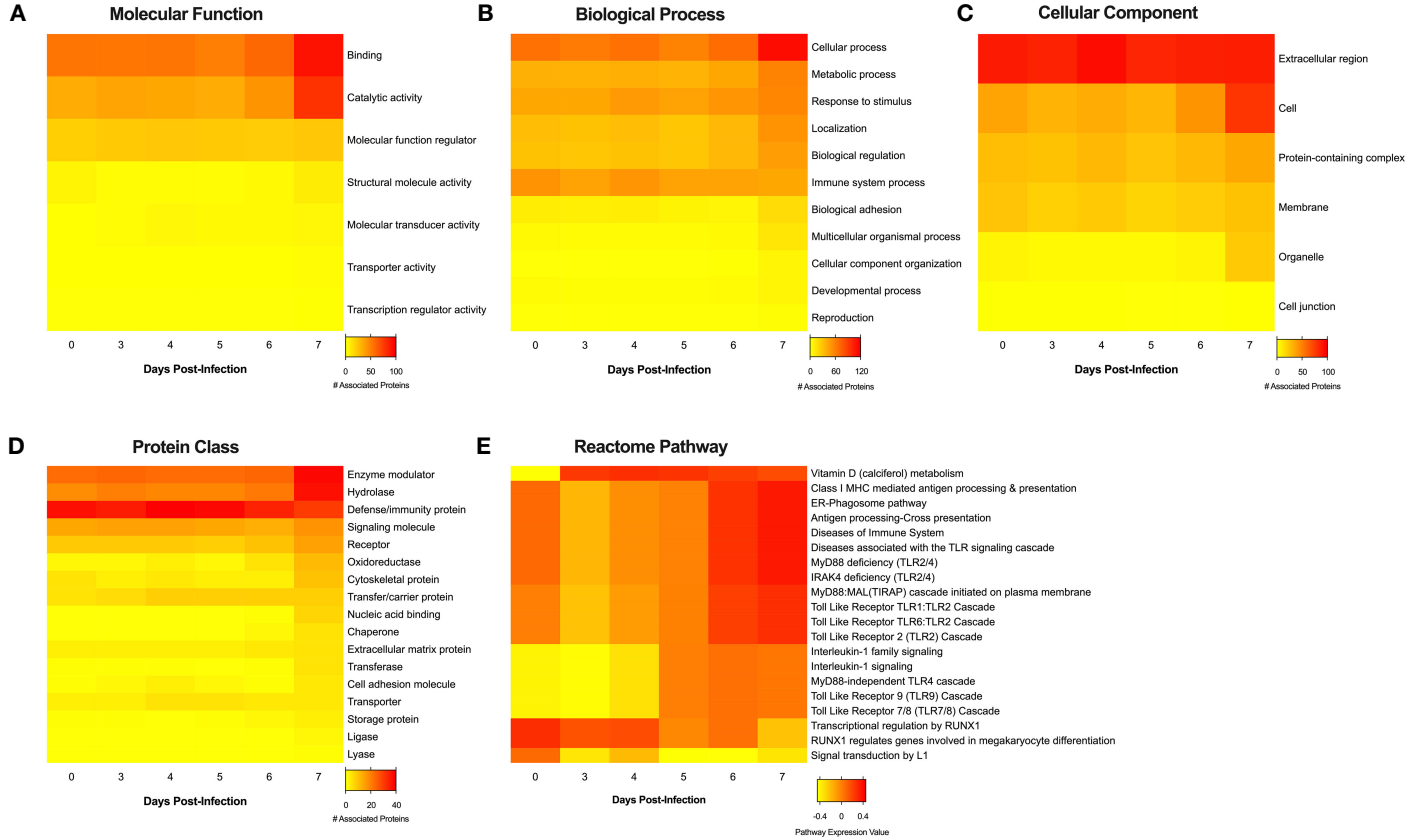
Functional annotation analyses of proteins identified in serum-derived EVs from EBOV-infected rhesus macaques over the course of infection. Upregulated proteins (averages greater or equal to the mean, *n* = 2, ANOVA with Benjamini-Hochberg correction for multiple comparisons, *p* < 0.05) from EVs isolated from serum collected on days 0, 3, 4, 5, 6 and 7 post-infection were classified using Gene Ontology analysis (Panther Database) according to **(A)** molecular function, **(B)** biological process, **(C)** cellular component, and **(D)** protein class. **(E)** Pathway expression coefficients were obtained by mapping entire protein sets to human orthologues (Blast2GO) followed by Gene Set Enrichment Analysis (Reactome Database). In all heatmaps, expression is indicated in a color scale ranging from yellow (low) to red (high).

Finally, analysis of the MS data set against the UniProt *Macaca mulatta* database allowed us to perform bioinformatic annotations of the proteins identified in the EVs released over the course of EBOV infection. The database revealed a total of 504 protein hits (417 individual proteins + 87 clusters) with a minimum average of 2 total spectrum counts between NHP 2401 and 2441 samples (S1 File). Gene ontology (GO) analysis was first performed on the complete proteome identified in EVs at different timepoints post-infection (D0, D3, D4, D5, D6, and D7), revealing a high degree of similarity between EVs from all stages of infection (**Figure S10**). Further analysis of proteins found to be enriched (high) in each group, according to statistically significant differences between groups (average of NHP 2401 and 2441) regarding total spectrum counts (ANOVA with Benjamini-Hochberg correction for multiple comparisons) revealed apparent differences in the functionality of the exoproteome over the course of EBOV infection (**Figure 8**). D7 EVs showed the most stimulatory profile across all protein classifications groups provided by PANTHER (**P**rotein **aN**alysis **TH**rough **E**volutionary **R**elationships) (Thomas et al., 2003). When classified according to their molecular functions, EV proteins associated with binding, catalytic activity, molecular function regulation and structural molecule activity generally increased with time post-infection, and were most expressed in D7 EVs, relative to other groups (**Figure 8A**). Further GO analysis revealed that the D7 exoproteome was enriched in proteins involved in almost all biological processes, including cellular and metabolic processes, response to stimulus, localization, biological regulation, biological adhesion, cellular component organization/biogenesis and developmental processes (**Figure 8B**). Similarly, EV proteins associated with cellular processes and responses to stimulus correlated with time post-infection (**Figure 8B**). In terms of cellular component, D7 EVs displayed the highest number of proteins involved in cell-cell junction, extracellular region, membrane, organelle, and protein-containing complex associated GO terms, and upregulation of EV proteins associated with the extracellular region was correlated with time post-infection (**Figure 8C**). This specific remark interestingly matches previous observations by our lab, in which exosomes from *Leishmania major* exhibited a stimulatory profile in terms of extracellular associated GO terms (Hassani et al., 2014). Next, when dissecting protein classes represented in the proteome, D7 EVs were the most enriched in proteins associated with cell adhesion molecule, chaperone, cytoskeletal protein, enzyme modulator, extracellular matrix protein, hydrolase, ligase, lyase, nucleic acid binding, oxidoreductase, receptor, signaling molecule, and transferase, compared to other groups (**Figure 8D**). Upregulation of proteins belonging to enzyme modulator and hydrolase protein classes also displayed a correlation with time post-EBOV infection (**Figure 8D**). Lastly, to obtain additional mechanistic insight into the variation in exoproteome content over the course of EBOV infection, we utilized Reactome Gene Set Enrichment Analysis (GSEA), which provides pathway expression levels for each group (Marc Gillespie et al., 2021). The most differentially regulated pathways reveal important modulation of pathways over the course of EBOV infection, including the downregulation of signal transduction by L1 and RUNX-1 mediated transcriptional regulation, and the upregulation of immune pathways and Vitamin D (calciferol) metabolism (**Figure 8E**). Of note, pathways involved in various stages of immunological response increase over the course of infection, including class I MHC antigen processing and presentation, the ER-phagosome, and multiple toll-like receptor pathways (**Figure 8E**).

Altogether, functional annotation of EV proteomes with Gene Ontology and Reactome assigned meaning to the differences observed in terms protein expression, which correspond to various stages of EBOV infection. Collectively, our data reveals that EVs are not only released in higher numbers towards latter timepoints of EVD in the NHP model, but that EVs from this late stage of infection (D7) possess a unique proteomic profile.

## Discussion

Ebola virus is the causative agent of human and non-human primate infections which dysregulate productive antiviral immune responses, leading to high case fatality rates (Hensley et al., 2002). Despite concerted research efforts into understanding the immunopathogenesis of the virus, the underlying mechanisms responsible for host susceptibility and disease progression remain elusive. Gaining a better understanding of host- and virus-derived factors that determine the severity of EVD can facilitate the development of novel antiviral therapeutics.

### GP exacerbates the immunostimulatory effect of EBOV VLPs *in vitro*

The first portion of our work utilized EBOV VLPs as a surrogate research model due to their morphological similarities to native filoviruses as well as BSL laboratory limitations (Noda et al., 2002). While VLPs can be generated with VP40 alone, co-expression of VP40 and NP enhances VLP release and forms structures that are more stable, organized and resemble authentic viral particles more closely (Licata et al., 2004; Johnson et al., 2006). Further, NP, which acts as the interface between several host and viral factors to enable viral transcription and replication, is unable to bud and form VLPs when expressed alone (Licata et al., 2004; Johnson et al., 2006). VLPs utilized in this study were therefore comprised of VP40 and NP, either alone (Bald VLP) or expressed along with GP (VLP-GP). Whereas some studies have explored the induction of pro-inflammatory mediators in the context of EBOV, the vast majority have been performed using epithelial cells, which are neither the primary targets of infection nor the most biologically relevant producers of these factors. We therefore opted for a the THP-1 human macrophage cell line, which expresses high levels of NOD1/NOD2 and TLR4 – the latter of which has been implicated in the induction of pro-inflammatory chemokines and cytokines by EBOV GP (Okumura et al., 2010; Escudero-Pérez et al., 2014; Lai et al., 2017). Stimulation of PMA-differentiated THP-1 monocytes with VLP-GP or Bald VLP revealed early upregulation of pro-inflammatory genes encoding TNF-α, IL-1β, IL-8 (MIP-2), MIP-1α, along with an increase in MIP-1β that was exacerbated by the presence of the GP. These results are in agreement with previous empirical knowledge that pro-inflammatory cytokines and chemokines are released by host cells early in infection as a means of attracting additional target cells, inducing vasodilation, and increasing vascular permeability to facilitate pathogenesis (Hensley et al., 2002; Bray and Geisbert, 2005). VLPs retain most of the functionality of EBOV aside from replication, though internalization efficiency is variable depending on cell type and origin. Previous studies have shown that the percentage of murine and human macrophages as well as Vero cells that uptake EBOV VLPs are 40%, 75%, and 50% respectively, and that the VLPs are particularly inefficient at entering undifferentiated monocytes (Martinez et al., 2013; Hu et al., 2019). Although VLP-GP was consistently more stimulatory across the inflammatory genes tested – though mostly not statistically significant – we also noted that Bald VLP displayed immunogenic functionality in both the murine B10R and human macrophages, which is suggestive of a potentially overlooked role of VP40 and NP proteins as EBOV virulence factors. The use of Bald-VLP as a control may also be further assessed, as the lack of GP may affect internalization efficiency, and other VLP systems for evaluating the immunostimulatory properties of GP may provide more adequate models of internalization and infection. Furthermore, these findings corroborate existing evidence that viral replication itself is not required for the predominantly GP-mediated release of pro-inflammatory cytokines and chemokines in the immediate cellular response, and simultaneously solidify the notion of the initial host-viral interaction as a crucial stage in the onset of pathology (Wahl-Jensen et al., 2011).

Impurities arising from the preparation and isolation of VLPs may also exert an effect on our models. Ultracentrifugation at 56,000 x g, which is utilized to concentrate VLPs, can sediment all major subtypes of cell derived EVs, including exosomes, ectosomes and apoptotic bodies (Kowal et al., 2016). Removal of all such EVs poses a particular challenge to the field, as they share similar biophysical characteristics to enveloped viruses (Minh et al., 2021). However, EVs derived from 293T cells display an uptake preference for recipient cells of the same cell line, and visualization of VLP preparations by TEM revealed very few co-sedimented EVs (Brian et al., 2020). Follow-up experiments assessing the role of EVs derived from VLP-transfected or VLP-infected cells may provide additional insight into the effect of GP-bearing EVs on the inflammatory response.

### EBOV VLPs are drivers of inflammation: both naked- and GP-VLPs can induce an inflammatory response *in vivo*, but lymphopenia is GP-dependent

Following our confirmation that our VLPs could induce an inflammatory response *in vitro*, we investigated their effect *in vivo* using an acute infection model. Intraperitoneal challenge with VLPs in BALB/c mice further allowed us to observe the effects of immediate host-viral interactions and offered new insight into early immune responses *in vivo.* Flow cytometry analysis revealed that both Bald VLP and VLP-GP facilitated significant recruitment of neutrophils and small peritoneal macrophages – a subset known to be recruited from circulating monocytes upon infection – into the peritoneum, 6 hours after injection (Ghosn et al., 2010). Although surprising given our initial hypothesis that the GP was mainly responsible for modulating innate cell recruitment, these findings substantiated our *in vitro* results that both VLPs were immunogenic in macrophages. Interestingly, analysis of pro-inflammatory gene expression showed that i.p. challenge with VLP-GP increased transcription of MCP-1 and MIP-1β genes, and significantly augmented the expression of IL-1β relative to the Bald VLP group. However, relative mRNA expression of the gene for MIP-1α, the macrophage-derived inflammatory mediator known for its role as a neutrophil and macrophage attractant, was similarly upregulated in mice injected with both VLPs (Geisbert et al., 2003; Zeng et al., 2003). These observations at the transcriptional level serve as preliminary indications explaining the comparable influx of neutrophils and inflammatory macrophages following challenge with VLPs, despite the array of genes tested being non-comprehensive. Quantification of protein levels is perhaps a more reliable measure of the immune changes occurring in the mouse peritoneal space in this context. Accordingly, we found that the production of TIMP-1, MCP-5, MDC, TARC, MIP-3α, and MIP-3β was analogously higher following challenge with Bald VLP or VLP-GP as compared to PBS-challenged mice, which again supports the flow cytometry data on neutrophil and small peritoneal macrophage influx to the peritoneum upon VLP challenge. Of these factors, gene expression of TIMP-1, TARC, MIP-3α, and MIP-3β was particularly upregulated in various cell and animal models of EBOV infection, although there was no inquiry into the subsequent effects at the protein level (Cilloniz et al., 2011; Olejnik et al., 2017; Speranza et al., 2018). MCP-5, the murine structural and functional homologue of human MCP-1, is of notable interest, as while it is known to be induced in activated macrophages under inflammatory conditions, it has not yet been reported in the context of EBOV infection (Sarafi et al., 1997). Thus, it may represent a novel marker of EBOV-induced inflammation in future mouse model studies (Sarafi et al., 1997). However, no effective quantification of peritoneal cavity levels of certain mediators such as IL-1β, TNF-α, MIP-1α, and MIP-1β, for example – which could have potentially uncovered a more robust impact of EBOV GP on the pro-inflammatory innate immune response – was obtained (Lai et al., 2017). This may be due, in part, to the 6-hour post-challenge timepoint, which could be too early for detection of such mediators, as peak concentrations can only be discerned 24-hours post-challenge in similar studies (Lai et al., 2017). Nevertheless, our data consistently followed a trend suggesting a GP-dependent loss of CD4^+^ T cells, CD8^+^ T cells, and B cells when investigating the effects of VLP challenge on lymphocyte populations in the mouse peritoneal cavity after 6 hours, though these changes were not significant. However, as the decrease in lymphocyte populations was not statistically significant, further investigation at later time-points post-challenge may indicate more important GP-mediated changes to lymphocyte populations. Lymphopenia is well-associated with fatal outcomes of EBOV infection in both animal models and human cases of EVD, and recent *in vitro* studies have shown evidence that the GP can directly trigger the death of T lymphocytes (Geisbert et al., 2000; Reed et al., 2004; Iampietro et al., 2017). Further, while previously thought to be incapable of infecting lymphocytes, more recent evidence indicates that EBOV is infectious to T cells – though this infection is not productive and induces host cell autophagy, mediated by GP and TLR-4/ER-stress (Younan et al., 2019). Our observations strengthen the traditional role of the GP as a critical and dynamic virulence factor; to our knowledge, no other groups have reported this decline in lymphocyte count within such a short period after GP (in our case, VLP-GP) exposure *in vivo* (Bradfute et al., 2007). Revisiting our measurements of chemokines, we found significantly higher levels of IP-10 in mice challenged with VLP-GP compared to Bald VLP. Previous reports have shown that IP-10 both contributes to activation-independent apoptosis (p38-mediated) of primary human T cells and becomes elevated in the context of chronic hepatitis C virus infection, leading to the sensitization of primary human CD4^+^ and CD8^+^ T cells to activation-induced apoptosis (Sidahmed et al., 2012; Zhao et al., 2013). These remarks may offer one possible explanation among many other proposed hypothetical mechanisms of action, in which lymphocyte populations are being depleted in early stages of host exposure to EBOV. Future studies using specific markers of apoptosis and necrosis, and varying stimulation times in mice, for example, should be performed to unpack these results.

Results indicating a role for VP40 and NP alone in the inflammatory response is particularly of interest, as GP is generally accepted to be EBOVs main immunogen (Pleet et al., 2019). Indeed, the role of EBOV GP has been well described to mediate viral entry into host cells, strongly implicated in the host pro-inflammatory response and cytokine storm, and even linked to T lymphocyte death *in vitro* (Wahl-Jensen et al., 2005; Lee and Saphire, 2009; Wahl-Jensen et al., 2011; Rhein and Maury, 2015; Iampietro et al., 2017). In fact, most current EBOV vaccine candidates utilize the GP as the only antigen (Marzi and Feldmann, 2014; Younan et al., 2018). However, given high dose requirements (~10^7^ PFU) to achieve appropriate immune protection, some adverse effects on the quality of T cell responses have been reported in a previous clinical trial of the VSV-vectored vaccine, which further supports the need to continue to decipher GP-mediated immune modulation and to concurrently uncover the role of other mediators crucial for host-viral interactions (Huttner et al., 2015; Younan et al., 2018). VP40, for example, is the most abundantly expressed protein in the EBOV virion and is responsible for viral budding from the inner leaflet of the plasma membrane (Noda et al., 2002; Stahelin, 2014). In addition, the matrix protein has been shown to play a role in cellular interactions upon binding/entry of viral particles and in the subsequent triggering of pro-inflammatory responses in macrophages, which is an observation we validated both in murine and human macrophages (Wahl-Jensen et al., 2011). Further, soluble VP40 has been detected outside of the viral particle in epithelial cells of EBOV-infected NHP and guinea pig models, which suggests a potentially unconventional role for the protein in viral pathogenesis, and additional studies have suggested that VP40-containing EVs may contribute to bystander T lymphocyte apoptosis (Reynard et al., 2011; Pleet et al., 2018). While our data did not reflect alterations to lymphocyte populations caused by Bald-VLP (VP40/NP), this mechanism may explain the upregulation of other inflammatory mediators at early timepoints post-stimulation. NP, on the other hand, which acts as the interface between viral and host-derived factors to facilitate transcription and translation, and is responsible for the formation of inclusion bodies, has not been reported to exert any direct effect on host immune function (Kruse et al., 2018; Morwitzer et al., 2019; Wendt et al., 2020). Its strong affinity for RNA, however, is non-specific to the viral genome, enabling the nucleoprotein to package and traffic host RNAs during VLP assembly– which could, in turn, potentiate the state of the transfected cells (Noda et al., 2010). While this could modulate the immune response, it is unclear whether non-specific binding of NP would favor any specific gene transcript in large enough copy numbers to exert a notable effect.

Being an enveloped virus, EBOV utilizes acquired phosphatidylserine (PS), the cytosolic leaflet lipid that is exposed on its outer membrane envelope, as one method to facilitate entry into new target cells via interaction with host receptor TIM-1 (Jemielity et al., 2013; Moller-Tank et al., 2013; Stahelin, 2014). Indeed, VLPs expressing VP40 and NP without the GP (Bald VLP) are capable of target cell internalization in a PS-dependent manner that may be an outcome of the intrinsic functional capacity of VP40 to encode for PS expression on the surface of VLPs (Jemielity et al., 2013; Moller-Tank et al., 2013; Stahelin, 2014). Due to the multiple essential roles of VP40 in the life cycle of EBOV and its potential for antigenicity, several studies have viewed it as a promising therapeutic target – via inhibition or modulation of phosphorylation – and vaccine antigen, although further investigation is required (Wilson et al., 2001; Madara et al., 2015; Monreal-Escalante et al., 2017). Altogether, our preliminary *in vitro* and *in vivo* work using EBOV VLPs has confirmed the effect of GP and suggested a role for VP40 in the characteristic inflammatory response of EVD, indicating that viral replication and the presence of genetic material can be, to a certain extent, uncoupled from the production of inflammatory mediators.

### EBOV infection induces a cytokine storm: production of classical mediators of inflammation increases as EVD progresses in NHPs

To further characterize the inflammatory response, we shifted towards the utilization of infective virus and to a model that more accurately represented human infection – non-human primates. NHPs are a gold-standard animal model to study EVD given their close resemblance to humans in terms of clinical disease and the pathophysiological processes that occur following EBOV infection (Bente et al., 2009; Geisbert et al., 2015). We conducted a longitudinal investigation of the rhesus macaque host response to progressive infection with *Zaire ebolavirus* Makona strain C07 focused on circulating inflammatory mediators and extracellular vesicles. Disease progression in the two rhesus macaques involved in our study largely recapitulated what was seen in many previous studies showing that the animals become viremic 3-5 days post-exposure (4 days in our study) and succumb to infection on days 5-9 post-inoculation (7 days in our study) (Bennett et al., 2017). Further, our observations agree with much of the reported literature on human and NHP cases of EVD, which demonstrates that the accumulation of circulating immune mediators commences 3-4 days post-inoculation, and that further changes correlate with disease progression; animals who die of disease typically exhibit significant increases in the serum concentrations of various pro-inflammatory cytokines and chemokines (Hensley et al., 2002; Wauquier et al., 2010; Versteeg et al., 2017; Banadyga et al., 2019). Indeed, we observed that the most considerable augmentation of pro-inflammatory IL-1β, IL-6, IL-15, IL-18, IFN-γ, eotaxin, GRO-α, IP-10, MCP-1, MIP-1α, MIP-1β, and regulatory IL-1RA, occurred at the terminal stage of the disease (7 days post-infection) in our rhesus macaques. Similar analyses have reported an increase in the production of these cytokines and chemokines in serum or plasma samples from humans and NHPs affected by fatal EBOV infection (Hensley et al., 2002; Wauquier et al., 2010; Versteeg et al., 2017; Banadyga et al., 2019). Of note, elevated levels of IFN-γ, its inducible chemokine IP-10 (CXCL10), IL-6 and IL-10 are hallmarks of cytokine storm disorders, correlating with our findings (Fajgenbaum and June, 2020). Levels of FGF-2, an indirectly pro-inflammatory growth factor capable of potentiating inflammatory mediator-induced recruitment of leukocytes, were also significantly higher at the terminal stage of the disease – data which we have yet to see documented in an EBOV context (Zittermann and Issekutz, 2006). In contrast, sCD40L, described to be immunosuppressive in cancer and HIV infection, was detected at very low levels on day 7 post-infection. This corroborates existing evidence that elevated sCD40L can be used as a biomarker of EVD survival (Huang et al., 2012; Jenabian et al., 2014; McElroy et al., 2014). Collectively, through the analysis of cytokines, chemokines, and other factors in circulation over the course of infection in the rhesus macaques, this branch of our study provided a viewpoint into the progression of the fatal disease, which was valuable to the subsequent exploration of host derived EVs.

### EBOV infection induces an “EV storm”: production of distinct EVs with an immunostimulatory profile increases as EVD progresses in NHPs

Following our study of classical inflammatory mediators, we sought to characterize unconventional effectors of inflammation and of disease states: extracellular vesicles. As previously described, EVs are a significant yet understudied factor in the pathogenesis of many infectious diseases, including EBOV and its virulence, as evidenced by a general lack of information and sparse empirical studies. To our knowledge, this is the first reported longitudinal characterization of EVs released during progressive *in vivo* infection with viable EBOV. NTA, TEM, and proteomic analyses of EVs isolated from the serum of the EBOV-infected rhesus macaques was performed to validate the purity of the preparation, according to international standards and guidelines (Théry et al., 2018). In both animals, an overall augmentation of EVs in circulation over the course of infection was observed, with a notable peak on the final day of the disease. Most of these vesicles fell within the size range of small EVs (< 200 nm diameter) and exosomes (30-150 nm in diameter). These findings are supported by earlier serum cytokine and chemokine analyses exhibiting a heightened pro-inflammatory response on the final day post-infection, in conjunction with the knowledge that EVs play a role in the pathogenesis of infection in numerous other studied diseases. Further, the notable drop in EV counts observed in both macaques at 5 days post-infection may be attributed to the downregulation of genes associated with translation and viral processes including translation initiation factors, elongation factors, and ribosomal proteins (Versteeg et al., 2017). This response suppression was previously detected by Versteeg et al. at similar time points following infection in cynomolgus macaques with the same EBOV strain – although the exact link between this prevention of EBOV replication by the host and the apparent reduction in EV release is still relatively unclear (Versteeg et al., 2017).

LC-MS/MS proteomic analysis corroborated previous findings indicating that EBOV proteins can be trafficked by host cells via exosomes (Pleet et al., 2016; Pleet et al., 2018). Among the proteins identified in circulatory EVs were VP40, NP, GP and VP30 – the latter of which is a critical mediator of viral transcription and of nucleocapsid assembly, which has not previously been identified in EV cargo (Wilson et al., 2001; Xu et al., 2017; Pleet et al., 2019). Interestingly, no EBOV proteins were detected prior to D6 post-infection, and spectral counts for VP30, VP40, and NP were particularly low (≤1 on D6 and D7). However, this is likely a product of the lack of sensitivity of LC-MS/MS for quantitation of low-abundance protein targets, as is the case with EBOV proteins in comparison to host-derived proteins, of which hundreds were detected in the EVs. In addition, while the frequency of EBOV protein integration into host derived EVs has yet to be characterized, it is likely a relatively small subpopulation of circulating EVs that derive from infected cells and carry viral cargo, further explaining this low protein abundance. EBOV GP, however, was much more abundant in the EVs, rapidly appearing at D6 and increasing in expression levels at D7 of EVD in both NHPs. This finding contradicts a previous report by Pleet et al. of VP40 as the most abundant EBOV protein in EVs derived from infected human umbilical vein endothelial cells, though differential viral protein trafficking may be cell-type specific and may vary over the course of disease progression (Pleet et al., 2018). While work by this group was key in showing the correlative expression of EBOV proteins in EVs over the course of EVD, their study on serum-derived EVs from EBOV-infected NHPs did not assess the presence of any other protein than VP40 and utilized pooled EVs from two macaques on different days post-infection (Pleet et al., 2018). Thus, our data indicating a rapid increase in EBOV GP expression in NHP EVs towards later stages of EVD suggests selective trafficking of GP and further corroborates the role of GP in EBOV pathogenesis.

Comparative analysis of the host-derived protein composition of all serum derived EVs revealed interesting trends in terms of uniqueness and levels of enrichment of proteins that were modified in the proteomes of EVs released at various time points pre- and post-infection. Most notably, we saw that EVs from day 7 post-infection were associated with the highest number of both unique and significantly enriched proteins, and that certain functional activities such as binding activity, catalytic activity, enzymatic activity (hydrolase), and enzyme modulator activity were significantly over-represented by these proteomes. However, progressive infection in general was enough to alter the protein composition of EVs across all time points. Our findings suggest that EVs in circulation during host infection with EBOV could potentially serve as biomarkers that reflect the physiological state of the cell; discernable increases in the number of unique proteins were noted in D4 EVs, relative to pre-infection, which reflects the typical onset of fever in the animals (4 days post-infection) (Versteeg et al., 2017). Further, proteins identified in the EVs from late stages of infection (day 6 and 7) were largely representative of an activated and inflamed host state. For example, neutrophil elastase (D7 EVs only) is associated with neutrophil-derived exosomes and involved in the promotion of vascular leakage and inflammation; pentraxin 3 is associated with hypoxia-induced exosomes and provides defense against infectious agents (D6 and D7 EVs only); while versican core protein is associated with exosomes derived from hepatitis B virus-infected cells and is recognized as a promoter of inflammation (D6 and D7 EVs only) (Kunes et al., 2012; Zhang et al., 2012; Kucharzewska et al., 2013; Doni et al., 2019; Genschmer et al., 2019). While the first two have not previously been identified in the context of EVD, versican has been shown to be upregulated in cells experimentally infected with EBOV, corroborating selective inflammatory cargo trafficking into EVs (Wynne et al., 2017).

Pathway analysis of the exoproteome provided additional mechanistic insight into the role of EVs during EVD progression. It is the upregulation of toll-like receptor pathways, which have been strongly associated to EVD, that is potentially the most striking and relevant in the context of EBOV infection (Barrenas et al., 2015; Amene Saghazadeh, 2017). Several TLRs have been associated to EVD, including TLR7/8 and TLR9, which stimulate the IRF7 pathway, TLR4 and TLR2, which are responsible for the MyD88-dependent pathway, and TLR 1 and TLR6, which have previously been reported to be upregulated during EBOV infection though their exact role remains elusive (Barrenas et al., 2015; Amene Saghazadeh, 2017). TLR4 is likely the most important of these, as it is widely accepted as a mediator of the inflammatory response to EBOV, through direct interaction with highly glycosylated molecules, such as viral GP (Escudero-Pérez et al., 2014). In fact, Volchkov et al. showed that pre-treatment of mice using an anti-TLR4 antibody completely prohibits the inflammatory effect of GP. Further, TLR4 and the ER-phagosome have been directly implicated in GP-dependent lymphocyte autophagy (Younan et al., 2019). Given EV cargo is enriched in mediators of these pathways at later timepoints post-infection, assessment of the capacity of these EVs to induce autophagy would be of substantial interest. While it is likely that multiple TLRs are involved in the systemic response to EBOV, shared downstream effectors between the distinct pathways may explain the number of receptors implicated in the response through bioinformatic annotation. The involvement of TLRs is corroborated by the induction of downstream effectors (including interferon-inducible genes), which we observed *in vitro* and *in vivo* in response to VP40/GP VLP stimulation and detected in increasing levels over the course of EBOV infection in rhesus macaques. Given that GP is likely expressed on the surface of EVs released from EBOV-infected cells, it follows that EVs themselves could stimulate a TLR-dependent inflammatory cascade (Pleet et al., 2019). Interleukin-production pathways are also shown to be increasingly expressed in the exoproteome over the course of EVD, further indicating the pro-inflammatory nature of EVs at later timepoints post-infection. The sustained upregulation of Vitamin D (Calciferol) metabolism-associated proteins in EVs following D0 post-infection was particularly unexpected, though Vitamin D has a relatively well-established role in innate and adaptive immune modulation, and has been associated to reduced mortality rates in patients with EVD when supplemented along with Vitamin A (Aranow, 2012; Aluisio et al., 2019). Inversely, pathways associated to the T cell differentiation transcription factor RUNX1 and to proliferative signaling by L1 are downregulated in EVs released over the course of EBOV infection. While some studies indicate that a decrease in RUNX1 signaling is indicative of increased cell death and diminished lymphocyte proliferation, which would correlate with late stage EVD’s characteristic lymphopenia, there is evidence that the opposite can also be true (Korinfskaya et al., 2021). Nonetheless, the concurrent decrease in L1 signaling suggests a decrease in proliferation of EV-producing cells, though L1 and RUNX1 have yet to be studied in the context of EBOV infection. Altogether, proteomic analysis of both viral and host-derived EV cargo indicates that exosomal trafficking of viral proteins – most notably GP – increases rapidly towards terminal stages of EVD, and that host proteins involved in various inflammatory processes are concurrently upregulated. The increase in expression of host-derived cargo involved in TLR4 signaling, among other immune pathways, suggests the promotion of inflammation by the EVs, which may contribute to the positive inflammatory feedback loop that is characteristic of EVD, in turn recruiting new target cells and inflammatory cells (Fisher-Hoch et al., 1985; Baize et al., 2002; Malvy et al., 2019). Secreted GP has also been shown to trigger a TLR4-dependent cascade, rendering target cells more susceptible to subsequent infection by EBOV (Iampietro et al., 2018). Other roles of secreted GP include the disruption of epithelial integrity, increasing vascular permeability, and the induction of immune activation of DCs, macrophages and monocytes (Okumura et al., 2010; Escudero-Pérez et al., 2014; Lai et al., 2017). Further, glycosylated GP is suspected to act as a decoy for antibodies over the course of infection, hindering an effective immune response (Gopi S. Mohan et al., 2012). It follows that host and viral factors trafficked via EVs may act in synergy to pre-emptively attract and modulate naïve target cells to create a permissive environment for EBOV entry and replication, effectively potentiating systemic infection and inflammation.

Returning to analyses of particle size distributions, we observed an overall shift towards slightly larger vesicles in the population throughout infection, which could also be linked to variations in EV protein cargo requirements as EVD progresses. Despite our best efforts, we did notice relatively high levels of macroglobulin and immunoglobulins in our proteomic analyses, which may have impacted the quality of our comparisons – particularly for those EVs isolated from early time points post-infection. Based on our evidence that the size distributions of vesicles did not immensely vary between fractions, it would perhaps be optimal to use only fraction 4 or 5 (most pure vesicle fraction) for proteomics moving forward, in order to achieve the lowest possible contamination (Böing et al., 2014). On the other hand, there is also the possibility that many of these detected immunoglobulins, for example, are related to B cell-derived exosomes (Saunderson et al., 2008). These exosomes are released when B lymphocytes are exposed to activating cytokine signals and characteristically express high levels of MHC class I, surface Ig (IgD, IgM, IgA, IgG1, IgG2a/2b, and IgG3), and tetraspanins, and have been implicated in antigen presentation (Saunderson et al., 2008). This is further corroborated by the upregulation of the Class I MHC mediated antigen presentation and processing pathways over the course of disease progression, although this avenue needs to be further explored under the conditions of EBOV infection.

Incidentally, other non-protein components of extracellular vesicles were not investigated in this study, including RNA species and lipids. These EV biomolecules present another layer of complexity that should be explored by RNA sequencing and lipidomics to better understand the functional and physiological consequences of EVs in the modulation of EBOV pathogenesis (Pathan et al., 2019). In addition, more work is planned to ascertain the differences in the capacity of the EVs from different time points post-infection to cause downstream effects on the host pro-inflammatory response, following stimulation in macrophages and the peritoneal cavity of mice.

### Conventional and unconventional mediators of inflammation work synergistically to favor the immunopathogenesis of EBOV

Collectively, findings stemming from our study highlight the importance of both viral and host factors in modulating the host inflammatory response to EBOV infection. Initial host-viral interactions through the GP and VP40/NP are crucial for affecting immune cells and precipitating the characteristic cytokine storm, while host-derived EVs play a dynamic and likely functional role over the course of progressive EBOV infection, themselves acting as an “EV storm”. Finally, further characterization to determine why such factors differ in their physiological effects, or work together to cumulatively influence disease severity, will allow for the development of novel vaccine candidates and antiviral therapeutics for patients affected by EVD.

## Data availability statement

The datasets presented in this study can be found in online repositories. The names of the repository/repositories and accession number(s) can be found in the article/**Supplementary Material**.

## Ethics statement

Ethical approval was not required for the studies on humans in accordance with the local legislation and institutional requirements because only commercially available established cell lines were used. The animal study was approved by Canadian Council of Animal Care Guidelines and Institutional Animal Care and Use Committees at McGill University under ethics protocol number 7791. The study was conducted in accordance with the local legislation and institutional requirements.

## Author contributions

AV: Conceptualization, Data curation, Formal Analysis, Investigation, Methodology, Validation, Writing – original draft. AL: Data curation, Formal Analysis, Writing – original draft, Writing – review & editing. MCo: Conceptualization, Funding acquisition, Writing – review & editing. DK: Conceptualization, Investigation, Methodology, Resources, Writing – review & editing. MCh: Data curation, Investigation, Methodology, Resources, Writing – review & editing. FA: Conceptualization, Data curation, Formal Analysis, Methodology, Writing – review & editing. CP: Resources, Writing – review & editing. GD: Data curation, Formal Analysis, Methodology, Writing – review & editing. MO: Conceptualization, Funding acquisition, Investigation, Methodology, Project administration, Resources, Supervision, Writing – original draft, Writing – review & editing.
