# Supplementary material for: Extracellular vesicle storm during the course of Ebola virus infection in primates
Source: Front Cell Infect Microbiol. 2023 Nov 15;13:1275277. doi: 10.3389/fcimb.2023.1275277 (PMC10684970; doi:10.3389/fcimb.2023.1275277)
Supplement: Supplementary file 1 [file DataSheet_1.pdf]

*Supplementary Material*

## Supplementary Text

### EBOV GP exacerbates VLP-induced pro-inflammatory response in murine-derived macrophages.

B10R murine macrophages were utilized to determine maximal activation conditions for human macrophage experiments given limited data surrounding EBOV-mediated chemokine and cytokine induction (26, 27). In the time-course experiment, B10R macrophages were either unstimulated (Nil; basal expression of 1) or stimulated with Bald VLP (5 μg/mL) for 30 minutes, 1, 2, or 4 hours, or LPS (100 ng/mL) for 1 hour as a positive control. Four hours after stimulation with Bald VLP, gene expression of TNF-α, MIP-1α (CCL3), and MIP-1β (CCL4) was significantly upregulated compared to the unstimulated group (~2-fold; $p < 0.05$; one-tailed unpaired t-test with Welch's correction), while gene expression of MCP-1 (CCL2; ~3-fold), MIP-2 (CXCL2; ~5-fold), and IL-1β (~3-fold) followed similar trends, as measured by qRT-PCR, though not statistically significant (S1A Fig). Relative mRNA levels of the pro-inflammatory genes tested suggest that a stimulation period of 4 hours led to the greatest activation of B10R cells in this experiment.

Next, we performed a dose-response experiment to confirm the optimal stimulation dose of VLPs required for maximal expression of selected pro-inflammatory genes. B10R macrophages were left unstimulated (Nil; basal expression of 1) or stimulated for 4 hours with increasing concentrations of Bald VLP or VLP-GP (1, 3, or 5 μg/mL), or LPS (100 ng/mL) as a positive control. All measured stimulation doses of Bald VLP and VLP-GP led to some upregulation of pro-inflammatory cytokine and chemokine gene expression compared to the unstimulated group, but strongest induction was most consistently observed with 5 μg/mL across all mediators, as measured by qRT-PCR (S1B Fig). Importantly, stimulation of cells with VLP-GP led to approximately 1.5-fold higher levels of gene expression of TNF-α ($p = 0.0572$), MCP-1 ($p = 0.0751$), MIP-1α ($p = 0.0632$), and MIP-1β ($p = 0.0404$), relative to Bald VLP at the highest concentration (5 μg/mL), suggesting that the GP is likely responsible for exacerbating the host pro-inflammatory immune response upon interaction with EBOV VLPs (one-tailed unpaired t-test with Welch's correction). While these data are only significant for

MIP-1β, this trend denotes a potential role for GP in the production of pro-inflammatory mediators. From these collective data, we determined that stimulation of cells with 5 μg/mL of VLPs for 4 hours or longer was optimal to achieve appropriate downstream immune activation for further studies *in vitro.*

**Circulating Extracellular vesicles can be purified from serum of EBOV-infected NHPs using size-exclusion chromatography.**

Given both the wide gap in understanding and the lack of empirical studies regarding the role of extracellular vesicles in the life cycle and overall pathogenesis of EBOV, we conducted a longitudinal investigation to characterize the host response to NHP infection with viable EBOV Makona strain C07, in the context of EVs. Gamma irradiated serum samples were collected from NHP 2401 and 2441 on day 0 (healthy control), and days 3, 4, 5, 6, and 7 post-infection. Irradiation enabled elimination of viral infectivity for subsequent experiments. EVs were purified from the serum samples by size-exclusion chromatography (SEC) – also known as "gel filtration" – as detailed in the materials and methods section (75). This methodology enabled EV isolation from limiting volumes of serum (250 μL) and took advantage of the capacity of SEC to allow for efficient recovery of EVs while preventing contamination from the majority of the soluble protein (76). The significant difference in size between the EVs and serum proteins permits the EVs to elute from the column first, while proteins elute in later fractions as they are smaller than the SEC matrix pores (76). By virtue of comprehensiveness, EVs derived from serum samples corresponding to day 0 (pre-infection), and days 3, 4, 5, 6, and 7 post-infection will be referred to as D0, D3, D4, D5, D6, and D7 EVs, respectively.

To appropriately distinguish EV-containing fractions from those harbouring the bulk of plasma-soluble proteins, 20 fractions from NHP 2401 serum collected 6 days after infection (D6) were collected. The concentration and size distribution of particles in these collected fractions were then assessed by nanoparticle tracking analysis (NTA), while protein content of each fraction was dosed by micro BCA assay (77). Following SEC, the highest concentration of particles was found in fractions 4-8, as measured by NTA (S4A Fig). Low levels of protein became detectable from fraction 7 or 8 onwards, while most of the protein eluted from fraction 10 onwards, as measured by micro-BCA (S4A Fig). When comparing the vesicle and protein distribution across all fractions, it is apparent that the presence of particles peaked at fraction 5, protein concentration was at its highest at fraction 15 (S4A Fig). This series of analyses was repeated with D6 serum from NHP 2441, which corroborated

these observations and confirmed that EVs can be separated from soluble protein by SEC (S4B Fig). Hence, all subsequent serum-derived EV analyses pertaining to this longitudinal study of EVD progression in NHP 2401 and 2441 were performed with only fractions 4-8 isolated with SEC.

Following the isolation of EVs from the time points before and after infection for both NHPs, we evaluated the purity of the vesicles by transmission electron microscopy of the pooled fractions of interest (4-8) and measured the size distribution and quantity of particles in each separate fraction by NTA. We observed similar morphological characteristics in the EV preparations purified from both NHPs across all timepoints, including the double-layered membrane (S5A-S6 Figs). The size distribution profiles of the particles present in each of the five fractions isolated from serum pre- and post-infection ($n$ = 2 macaques, 6 serum samples each) further confirmed the presence of EVs (S5B and S7A-B Figs). Notably, the population of particles present at the highest concentration in almost all fractions across all collection points before and after infection had diameters that fell within the size range of ~80-200 nm – characteristic of small EVs (< 200 nm) (54).The exceptions to this observation were a select few fractions collected from serum from day 0 and day 3 post-infection (NHP 2401 D0 fractions 4-5, D3 fraction 4; NHP 2441 D0 fraction 8) (S7A-B Fig). However, these fraction samples contained lower concentrations of particles (~$10^6$ particles/mL) relative to other samples, which may limit the accuracy of quantification by the NanoSight instrument and NTA software (78).

**Size-exclusion chromatography enables recovery of complete populations of circulatory EVs in an NHP model of EVD progression.**

To validate our purification methodology and characterization of EVs in terms of quantification via particle number, we sought to determine the concentration of particles in the starting material (unmanipulated serum) collected on day 0 (pre-infection) and days 3, 5, and 7 post-infection. Since these samples were not purified or modified, the particles detected by NTA are not necessarily small EVs, and thus may include protein aggregates (75). As observed with the isolated EVs, serum from day 7 post-infection (death of both animals) contained the highest number of particles (S8A Fig). Further, the increase in the concentration of particles on day 7 was significant compared to day 0 ($p < 0.01$; repeated measures one-way ANOVA with Dunnett's correction for multiple comparisons); the average concentration of particles in circulation on day 3 was also higher than pre-infection, although not statistically significant. Remarkably, analysis of the starting material corroborated the previous observation made with EVs, in which the number of particles decreases on day 5 (relative to day 3),

rather than increasing in a stepwise pattern over time, as was hypothesized. Of note, due to the severe nature of the disease in the animals 7 days post-infection, it Is critical to consider the possibility that there may be many serum protein aggregates contributing to the extremely high particle counts in the starting material ($\sim 10^{12}$). Overall, the size distributions of particles corresponding to the four measured time points pre- and post-infection were relatively similar between EV-containing fraction 6 (Fig 6C) and the starting material (S8B Fig), as measured by NTA.

<u>**Supplementary Tables**</u>

**Supplementary Table 1.** Unique proteins to D0 EVs relative to all other days post-EBOV infection.

| Identified Protein [Macaca mulatta] | Accession Number | Molecular Weight | Total Spectrum Counts | | | |
|---|---|---|---|---|---|---|
| | | | NHP 2401 D0 | NHP 2441 Counts | Average Day 0 | Standard Deviation |
| Immunoglobulin heavy chain variable region, partial | AKE97596.1 | 13 kDa | 0 | 70 | 35 | 49.50 |
| Immunoglobulin heavy chain variable region, partial | AHN95047.1 | 13 kDa | 0 | 69 | 34.5 | 48.79 |
| Immunoglobulin heavy chain variable region, partial | ATV91258.1 | 11 kDa | 59 | 0 | 29.5 | 41.72 |
| Immunoglobulin heavy chain variable region, partial | AHN94658.1 | 13 kDa | 53 | 0 | 26.5 | 37.48 |
| Immunoglobulin heavy chain, partial | ACN96915.1 | 22 kDa | 41 | 0 | 20.5 | 28.99 |
| Immunoglobulin heavy chain variable region, partial | AKE98177.1 | 14 kDa | 0 | 27 | 13.5 | 19.09 |
| Immunoglobulin heavy chain, partial | AAR83990.1 | 16 kDa | 26 | 0 | 13 | 18.38 |
| Immunoglobulin kappa variable region, partial | AWN00198.1 | 12 kDa | 20 | 0 | 10 | 14.14 |
| Immunoglobulin heavy chain variable region, partial | AHN95252.1 | 13 kDa | 0 | 19 | 9.5 | 13.44 |
| Anti-SIV env immunoglobulin ITS16.01 light chain variable region, partial | AMZ04084.1 | 12 kDa | 19 | 0 | 9.5 | 13.44 |
| Immunoglobulin heavy chain variable region, partial | ARO71155.1 | 11 kDa | 0 | 15 | 7.5 | 10.61 |
| Immunoglobulin heavy chain variable region, partial | AKE98020.1 | 13 kDa | 0 | 11 | 5.5 | 7.78 |
| Immunoglobulin heavy chain variable region, partial | ANZ54631.1 | 11 kDa | 0 | 10 | 5 | 7.07 |
| Immunoglobulin kappa light chain variable region, partial | ASR74653.1 | 12 kDa | 10 | 0 | 5 | 7.07 |
| Immunoglobulin heavy chain variable segment precursor, partial | AAF89366.1 | 13 kDa | 8 | 0 | 4 | 5.66 |
| Immunoglobulin G light chain variable region, partial | AMS25321.1 | 11 kDa | 0 | 8 | 4 | 5.66 |
| Immunoglobulin G heavy chain variable region, partial | AMS24937.1 | 13 kDa | 8 | 0 | 4 | 5.66 |
| PREDICTED: multimerin-1 | XP_014994280.1 | 138 kDa | 5 | 3 | 4 | 1.41 |
| Immunoglobulin heavy chain variable region, partial | ATV91294.1 | 9 kDa | 0 | 7 | 3.5 | 4.95 |
| Immunoglobulin kappa chain variable region | EHH25606.1/ ATV91337.1 | 11 kDa | 7 | 0 | 3.5 | 4.95 |
| Immunoglobulin heavy chain variable region, partial | AKE97878.1 | 14 kDa | 0 | 6 | 3 | 4.24 |
| CD9 antigen | NP_001247590.1 | 25 kDa | 6 | 0 | 3 | 4.24 |
| Immunoglobulin heavy chain, partial | AER46899.1 | 15 kDa | 0 | 6 | 3 | 4.24 |
| Immunoglobulin heavy chain variable region, partial | AHN94776.1 | 13 kDa | 0 | 5 | 2.5 | 3.54 |
| Immunoglobulin kappa light chain variable region, partial | APG53617.1 | 10 kDa | 0 | 5 | 2.5 | 3.54 |
| Immunoglobulin G light chain variable region, partial | AMS24876.1 | 12 kDa | 0 | 5 | 2.5 | 3.54 |
| Immunoglobulin heavy chain variable region, partial | ATV91224.1 | 11 kDa | 4 | 0 | 2 | 2.83 |
| Immunoglobulin kappa chain variable region, partial | ATV91080.1 | 13 kDa | 4 | 0 | 2 | 2.83 |
| Immunoglobulin G light chain variable region, partial | AMS25506.1 | 12 kDa | 4 | 0 | 2 | 2.83 |

**Supplementary Table 2.** Unique proteins to D3 EVs relative to all other days post-EBOV infection.

| Identified Protein [Macaca mulatta] | Accession Number | Molecular Weight | Total Spectrum Counts | | | |
|---|---|---|---|---|---|---|
| | | | NHP 2401 D3 | NHP 2441 D3 | Average D3 | Standard Deviation |
| Immunoglobulin lambda light chain, partial | ACN96934.1 | 21 kDa | 281 | 0 | 141 | 198.70 |
| Immunoglobulin heavy chain, partial | AAC02643.1 | 16 kDa | 43 | 0 | 21.5 | 30.41 |
| Immunoglobulin kappa light chain variable region, partial | APG53644.1 | 11 kDa | 18 | 19 | 18.5 | 0.71 |
| Immunoglobulin G light chain variable region, partial | AMS25162.1 | 12 kDa | 18 | 0 | 9.0 | 12.73 |
| Immunoglobulin heavy chain variable region, partial | ANZ54652.1 | 10 kDa | 0 | 14 | 7.0 | 9.90 |
| Immunoglobulin heavy chain, partial | AER46744.1 | 15 kDa | 0 | 8 | 4.0 | 5.66 |
| Immunoglobulin heavy chain variable region, partial | AKE98070.1 | 14 kDa | 0 | 8 | 4 | 5.66 |
| Immunoglobulin heavy chain variable region, partial | AKE98303.1 | 12 kDa | 0 | 6 | 3 | 4.24 |
| Immunoglobulin G heavy chain variable region, partial | AMS24992.1 | 14 kDa | 6 | 0 | 3.0 | 4.24 |
| Immunoglobulin heavy chain variable region | EHH25651.1/ ATV91065.1 | 13 kDa | 5 | 0 | 2.5 | 3.54 |
| Immunoglobulin kappa light chain variable region, partial | ANC49889.1 | 12 kDa | 0 | 5 | 2.5 | 3.54 |
| Cytokeratin-24 | EHH24921.1 | 55 kDa | 5 | 0 | 2.5 | 3.54 |
| Immunoglobulin heavy chain variable region, partial | AVN88743.1 | 13 kDa | 4 | 0 | 2 | 2.83 |
| Immunoglobulin kappa light chain, partial | AER46502.1 | 13 kDa | 4 | 0 | 2 | 2.83 |
| Immunoglobulin kappa light chain, partial | AER46518.1 | 13 kDa | 4 | 0 | 2 | 2.83 |
| Immunoglobulin kappa light chain, partial | AER46598.1 | 14 kDa | 4 | 0 | 2 | 2.83 |
| Immunoglobulin heavy chain variable region, partial | AKE97895.1 | 13 kDa | 0 | 4 | 2 | 2.83 |
| PREDICTED: corticosteroid-binding globulin | XP_001098128.1 | 45 kDa | 4 | 0 | 2 | 2.83 |
| PREDICTED: protein S100-A7 | XP_001102452.2 | 12 kDa | 4 | 0 | 2.0 | 2.83 |

**Supplementary Table 3.** Unique proteins to D4 EVs relative to all other days post-EBOV infection.

| Identified Protein [Macaca mulatta] | Accession Number | Molecular Weight | Total Spectrum Counts | | | |
|---|---|---|---|---|---|---|
| | | | NHP 2401 D4 | NHP 2441 D4 | Average | Standard Deviation |
| Immunoglobulin lambda light chain, partial | ACN96972.1 | 22 kDa | 284 | 0 | 142 | 200.82 |
| Immunoglobulin lambda light chain, partial | ACN96977.1 | 21 kDa | 0 | 258 | 129.0 | 182.43 |
| Immunoglobulin kappa light chain, partial | ACN96958.1 | 21 kDa | 0 | 121 | 60.5 | 85.56 |
| Immunoglobulin heavy chain, partial | AAR83983.1 | 16 kDa | 0 | 105 | 52.5 | 74.25 |
| Immunoglobulin heavy chain variable region, partial | ALW83516.1 | 13 kDa | 0 | 73 | 36.5 | 51.62 |
| Chain H, DH522UCA Fab fragment heavy chain | pdb\|5UKN\|H | 24 kDa | 61 | 0 | 30.5 | 43.13 |
| Immunoglobulin lambda light chain, partial | AER46314.1 | 13 kDa | 0 | 30 | 15 | 21.21 |
| Immunoglobulin heavy chain variable region, partial | AKE97669.1 | 14 kDa | 27 | 0 | 14 | 19.09 |
| Immunoglobulin heavy chain variable region, partial | ARO71081.1 | 13 kDa | 26 | 0 | 13.0 | 18.38 |
| Immunoglobulin heavy chain variable region, partial | ATV90971.1 | 13 kDa | 11 | 14 | 12.5 | 2.12 |
| Immunoglobulin heavy chain variable region, partial | AKE97478.1 | 13 kDa | 0 | 24 | 12.0 | 16.97 |
| Immunoglobulin kappa light chain variable region, partial | APG53655.1 | 10 kDa | 22 | 0 | 11.0 | 15.56 |
| Immunoglobulin G light chain variable region, partial | AMS25079.1 | 12 kDa | 19 | 0 | 10 | 13.44 |
| Immunoglobulin heavy chain variable region, partial | AHN94873.1 | 13 kDa | 18 | 0 | 9 | 12.73 |
| Immunoglobulin heavy chain variable region, partial | AKC54351.1 | 10 kDa | 0 | 14 | 7 | 9.90 |
| Immunoglobulin G light chain variable region, partial | AMS24779.1 | 12 kDa | 13 | 0 | 7 | 9.19 |
| Immunoglobulin G light chain variable region, partial | AMS24726.1 | 11 kDa | 0 | 13 | 7 | 9.19 |
| Immunoglobulin G light chain variable region, partial | AMS25499.1 | 12 kDa | 7 | 0 | 4 | 4.95 |
| Immunoglobulin lamda light chain variable region, partial | ASR74637.1 | 12 kDa | 5 | 0 | 2.5 | 3.54 |
| Immunoglobulin heavy chain variable region | EHH28237.1/ ATV90954.1 | 13 kDa | 5 | 0 | 2.5 | 3.54 |
| Cytokeratin-6C | EHH20755.1 | 60 kDa | 5 | 0 | 3 | 3.54 |
| Immunoglobulin G light chain variable region, partial | AMS24740.1 | 12 kDa | 2 | 3 | 3 | 0.71 |
| Anti-SIV env immunoglobulin ITS08 light chain variable region, partial | AMZ04072.1 | 12 kDa | 0 | 5 | 3 | 3.54 |
| Immunoglobulin heavy chain variable region, partial | AAO43419.1 | 11 kDa | 0 | 5 | 2.5 | 3.54 |
| Immunoglobulin heavy chain, partial | AAC02642.1 | 16 kDa | 0 | 4 | 2.0 | 2.83 |
| Immunoglobulin heavy chain variable region, partial | ATV91124.1 | 13 kDa | 0 | 4 | 2.0 | 2.83 |
| Immunoglobulin gamma variable region, partial | AWN00208.1 | 13 kDa | 0 | 4 | 2 | 2.83 |
| Immunoglobulin kappa chain variable region, partial | ATV91469.1 | 9 kDa | 0 | 4 | 2 | 2.83 |
| Pleckstrin | AFH29190.1 | 40 kDa | 0 | 4 | 2 | 2.83 |
| Immunoglobulin G light chain variable region, partial | AMS25466.1 | 12 kDa | 4 | 0 | 2 | 2.83 |
| Cluster of immunoglobulin kappa light chain variable region, partial (APG53735.1) | APG53735.1 | 10 kDa | 4 | 0 | 2 | 2.83 |
| Immunoglobulin kappa light chain variable region, partial | APG53735.1 | 10 kDa | 4 | 0 | 2 | 2.83 |

| | | | | | | |
|---|---|---|---|---|---|---|
| Immunoglobulin G light chain variable region, partial | AMS24744.1 | 12 kDa | 0 | 4 | 2 | 2.83 |
| Cluster of immunoglobulin lambda chain variable region, partial (ATV91558.1) | ATV91558.1 | 10 kDa | 0 | 4 | 2 | 2.83 |

**Supplementary Table 4.** Unique proteins to D5 EVs relative to all other days post-EBOV infection.

| Identified Protein [Macaca mulatta] | Accession Number | Molecular Weight | Total Spectrum Counts | | | |
|---|---|---|---|---|---|---|
| | | | NHP 2401 D5 | NHP 2441 D5 | Average | Standard Deviation |
| Chain B, Light Chain Of Fab Fragment Of Hiv Vaccine-elicited Cd4bs- Directed Antibody | pdb\|4Q2Z\|B | 23 kDa | 152 | 0 | 76 | 107.48 |
| Immunoglobulin heavy chain variable region, partial | AHN95196.1 | 13 kDa | 0 | 56 | 28.0 | 39.60 |
| Immunoglobulin lamda variable region, partial | AWN00195.1 | 12 kDa | 0 | 33 | 16.5 | 23.33 |
| Immunoglobulin heavy chain variable region, partial | AVN88741.1 | 14 kDa | 18 | 0 | 9.0 | 12.73 |
| Immunoglobulin kappa chain variable region, partial | ARW79889.1 | 11 kDa | 15 | 0 | 7.5 | 10.61 |
| Immunoglobulin G heavy chain variable region, partial | AMS24925.1 | 13 kDa | 0 | 11 | 5.5 | 7.78 |
| Immunoglobulin G light chain variable region, partial | AMS24869.1 | 11 kDa | 9 | 0 | 5 | 6.36 |
| Immunoglobulin G light chain variable region, partial | AMS24751.1 | 12 kDa | 8 | 0 | 4 | 5.66 |
| Immunoglobulin kappa chain variable region, partial | ATV91457.1 | 10 kDa | 5 | 3 | 4.0 | 1.41 |
| Immunoglobulin heavy chain variable region, partial | ARO71089.1 | 14 kDa | 0 | 7 | 3.5 | 4.95 |
| Anti-SIV env immunoglobulin ITS03 heavy chain variable region, partial | AMZ03999.1 | 13 kDa | 7 | 0 | 3.5 | 4.95 |
| Cluster of immunoglobulin G light chain variable region, partial (AMS24814.1) | AMS24814.1 | 12 kDa | 0 | 6 | 3.0 | 4.24 |
| Immunoglobulin G light chain variable region, partial | AMS24814.1 | 12 kDa | 0 | 6 | 3 | 4.24 |
| Immunoglobulin heavy chain variable region, partial | ATV90968.1 | 13 kDa | 5 | 0 | 3 | 3.54 |
| Immunoglobulin heavy chain variable region, partial | AHN94834.1 | 14 kDa | 5 | 0 | 3 | 3.54 |
| Immunoglobulin G light chain variable region, partial | AMS25457.1 | 11 kDa | 0 | 5 | 3 | 3.54 |
| Immunoglobulin kappa chain variable region, partial | ATV91452.1 | 10 kDa | 5 | 0 | 3 | 3.54 |
| Immunoglobulin light chain variable region, partial | AKP06489.1 | 12 kDa | 5 | 0 | 3 | 3.54 |
| Immunoglobulin heavy chain variable region, partial | AHN94608.1 | 13 kDa | 5 | 0 | 2.5 | 3.54 |
| PREDICTED: complement receptor type 2 isoform X1 | XP_014973177.1 | 120 kDa | 3 | 2 | 2.5 | 0.71 |
| Anti-HIV immunoglobulin heavy chain variable region, partial | AIT16780.1 | 15 kDa | 4 | 0 | 2 | 2.83 |
| Immunoglobulin heavy chain variable region, partial | AHN94737.1 | 14 kDa | 0 | 4 | 2 | 2.83 |
| Anti-SIV env immunoglobulin ITS02 light chain variable region, partial | AMZ04066.1 | 12 kDa | 4 | 0 | 2 | 2.83 |
| Immunoglobulin heavy chain variable region, partial | AHN94343.1 | 13 kDa | 4 | 0 | 2.0 | 2.83 |
| Anti-SIV env immunoglobulin ITS30 light chain variable region, partial | AMZ04091.1 | 12 kDa | 0 | 4 | 2.0 | 2.83 |
| Immunoglobulin G light chain variable region, partial | AMS25430.1 | 13 kDa | 4 | 0 | 2.0 | 2.83 |
| Immunoglobulin G heavy chain variable region, partial | AMS25035.1 | 14 kDa | 4 | 0 | 2 | 2.83 |

**Supplementary Table 5.** Unique proteins to D6 EVs relative to all other days post-EBOV infection.

| Identified Protein [Macaca mulatta] | Accession Number | Molecular Weight | Total Spectrum Counts | | | |
|---|---|---|---|---|---|---|
| | | | NHP 2401 D6 | NHP 2441 D6 | Average | Standard Deviation |
| Immunoglobulin kappa light chain, partial | ACN96919.1 | 21 kDa | 0 | 122 | 61 | 86.27 |
| Immunoglobulin heavy chain variable region, partial | AHN94369.1 | 14 kDa | 57 | 0 | 28.5 | 40.31 |
| Immunoglobulin heavy chain variable region, partial | AKE97944.1 | 14 kDa | 53 | 0 | 26.5 | 37.48 |
| Immunoglobulin lambda light chain, partial | AER46326.1 | 12 kDa | 0 | 23 | 11.5 | 16.26 |
| Immunoglobulin G heavy chain variable region, partial | AMS25009.1 | 14 kDa | 0 | 20 | 10.0 | 14.14 |
| Immunoglobulin lambda light chain, partial | AER46294.1 | 13 kDa | 0 | 20 | 10.0 | 14.14 |
| Hyaluronan-binding protein 2 isoform X1 | EHH19382.1/ XP_001090138.2 | 63 kDa | 9 | 9 | 9 | 0.00 |
| Immunoglobulin heavy chain variable region, partial | AHN94932.1 | 13 kDa | 0 | 11 | 6 | 7.78 |
| Immunoglobulin heavy chain variable region, partial | AHN95068.1 | 13 kDa | 11 | 0 | 5.5 | 7.78 |
| Immunoglobulin heavy chain variable region, partial | AKE98226.1 | 13 kDa | 0 | 7 | 3.5 | 4.95 |
| Cluster of glycogen phosphorylase, muscle form (NP_001248463.1) | NP_001248463.1 | 97 kDa | 5 | 2 | 3.5 | 2.12 |
| Glycogen phosphorylase, muscle form | NP_001248463.1 | 97 kDa | 5 | 2 | 3.5 | 2.12 |
| Immunoglobulin heavy chain variable region, partial | AKE97527.1 | 13 kDa | 6 | 0 | 3 | 4.24 |
| Immunoglobulin light chain variable region, partial | AKP06492.1 | 11 kDa | 0 | 6 | 3 | 4.24 |
| Immunoglobulin G heavy chain variable region, partial | AMS24986.1 | 13 kDa | 0 | 5 | 3 | 3.54 |
| Platelet glycoprotein Ib alpha polypeptide | ACJ38405.1 | 82 kDa | 5 | 0 | 3 | 3.54 |
| Anti-SIV env immunoglobulin ITS52 light chain variable region, partial | AMZ04104.1 | 12 kDa | 0 | 4 | 2 | 2.83 |
| Immunoglobulin heavy chain variable region, partial | ATV91244.1 | 11 kDa | 4 | 0 | 2 | 2.83 |

**Supplementary Table 6.** Unique proteins to D7 EVs relative to all other days post-EBOV infection.

| Identified Protein [Macaca mulatta] | Accession Number | Molecular Weight | Total Spectrum Counts | | | |
|---|---|---|---|---|---|---|
| | | | NHP 2401 D7 | NHP 2441 D7 | Average | Standard Deviation |
| MHC class I protein | SOF04253.1 | 41 kDa | 60 | 0 | 30 | 42.43 |
| MHC class I antigen, partial | ACA65899.1 | 40 kDa | 45 | 13 | 29.0 | 22.63 |
| MHC class I antigen, partial | ACF93206.1 | 39 kDa | 39 | 18 | 28.5 | 14.85 |
| Histone H2B type 2-E, partial | AFE72265.1 | 11 kDa | 29 | 14 | 21.5 | 10.61 |
| MHC class I antigen, partial | ABQ41415.1 | 39 kDa | 37 | 0 | 18.5 | 26.16 |
| MHC class I antigen, partial | ABQ41418.1 | 39 kDa | 32 | 0 | 16.0 | 22.63 |
| Cluster of tubulin beta-2B chain (AFI36483.1) | AFI36483.1 | 50 kDa | 20 | 11 | 16 | 6.36 |
| Tubulin beta-2B chain | AFI36483.1 | 50 kDa | 20 | 11 | 16 | 6.36 |
| Cluster of PREDICTED: histone H3.1-like (XP_002803695.2) | XP_002803695.2 | 28 kDa | 17 | 12 | 14.5 | 3.54 |
| PREDICTED: histone H3.1-like | XP_002803695.2 | 28 kDa | 17 | 12 | 14.5 | 3.54 |
| PREDICTED: amiloride-sensitive amine oxidase [copper-containing] | XP_014990764.1 | 86 kDa | 20 | 6 | 13.0 | 9.90 |
| Heat shock 70 kda protein 1/2 | EHH18181.1 | 70 kDa | 13 | 12 | 12.5 | 0.71 |
| PREDICTED: carbamoyl-phosphate synthase [ammonia], mitochondrial | XP_001110375.1 | 165 kDa | 6 | 17 | 12 | 7.78 |
| Guanine nucleotide-binding protein G(i) subunit alpha-2 isoform 1, partial | AFE71714.1 | 40 kDa | 16 | 7 | 12 | 6.36 |
| PREDICTED: neutrophil elastase | XP_014977795.1 | 28 kDa | 15 | 7 | 11 | 5.66 |
| Unc-112-related protein 2 | EHH22743.1 | 76 kDa | 17 | 4 | 11 | 9.19 |
| ADP-ribosylation factor 1 | NP_001180216.1 | 21 kDa | 15 | 5 | 10 | 7.07 |
| Cluster of integrin beta-2 precursor (AFH32301.1) | AFH32301.1 | 85 kDa | 10 | 7 | 9 | 2.12 |
| Integrin beta-2 precursor | AFH32301.1 | 85 kDa | 10 | 7 | 8.5 | 2.12 |
| PREDICTED: receptor-type tyrosine-protein phosphatase C isoform X1 | XP_014976209.1 | 148 kDa | 14 | 3 | 8.5 | 7.78 |
| Cell division control protein 42 homolog | NP_001244809.1 | 21 kDa | 11 | 6 | 8.5 | 3.54 |
| Cytosol aminopeptidase | NP_001247627.1 | 56 kDa | 11 | 5 | 8 | 4.24 |
| Fc gamma receptor RII, partial | AEC03703.1 | 31 kDa | 8 | 8 | 8 | 0 |
| Immunoglobulin kappa light chain variable region, partial | APG53699.1 | 11 kDa | 15 | 0 | 7.5 | 10.61 |
| Guanine nucleotide-binding protein G(I)/G(S)/G(T) subunit beta-1 | AFI33718.1 | 37 kDa | 11 | 4 | 7.5 | 4.95 |
| Cluster of PREDICTED: integrin alpha-M isoform X1 (XP_014981504.1) | XP_014981504.1 | 128 kDa | 7 | 8 | 7.5 | 0.71 |
| PREDICTED: integrin alpha-M isoform X1 | XP_014981504.1 | 128 kDa | 7 | 8 | 7.5 | 0.71 |
| Immunoglobulin G heavy chain variable region, partial | AMS24956.1 | 13 kDa | 14 | 0 | 7 | 9.9 |
| Immunoglobulin kappa chain variable region, partial | ATV91410.1 | 10 kDa | 0 | 14 | 7 | 9.9 |
| Catalase | EHH22994.1 | 60 kDa | 14 | 0 | 7 | 9.9 |
| Cluster of ras-related protein Rab-1B (NP_001253093.1) | NP_001253093.1 | 22 kDa | 10 | 3 | 6.5 | 4.95 |
| PREDICTED: fructose-bisphosphate aldolase A isoform X2 | XP_014981390.1 | 39 kDa | 9 | 4 | 6.5 | 3.54 |
| Cluster of PREDICTED: transforming protein rhoa (XP_014986395.1) | XP_014986395.1 | 22 kDa | 9 | 4 | 6.5 | 3.54 |
| MHC class I antigen, partial | AFV31657.1 | 41 kDa | 0 | 12 | 6 | 8.49 |
| Vascular cell adhesion protein 1 isoform a precursor | AFH33883.1 | 81 kDa | 8 | 4 | 6 | 2.83 |
| Platelet glycoprotein 4 | NP_001028085.1 | 53 kDa | 7 | 5 | 6 | 1.41 |
| Cluster of alcohol dehydrogenase 1B (NP_001247556.1) | NP_001247556.1 | 40 kDa | 7 | 5 | 6 | 1.41 |
| Alcohol dehydrogenase 1B | NP_001247556.1 | 40 kDa | 7 | 5 | 6 | 1.41 |

| | | | | | | |
|---|---|---|---|---|---|---|
| PREDICTED: transforming protein rhoa | XP_014986395.1 | 22 kDa | 8 | 4 | 6 | 2.83 |
| Aspartyl aminopeptidase | AFH29347.1 | 53 kDa | 8 | 4 | 6 | 2.83 |
| Tenascin | EHH23833.1 | 241 kDa | 7 | 4 | 5.5 | 2.12 |
| Cluster of ras-related C3 botulinum toxin substrate 2 (NP_001248306.1) | NP_001248306.1 | 21 kDa | 6 | 4 | 5 | 1.41 |
| Ras-related C3 botulinum toxin substrate 2 | NP_001248306.1 | 21 kDa | 6 | 4 | 5 | 1.41 |
| Ras-related protein Rab-5C | NP_001253100.1 | 23 kDa | 7 | 3 | 5 | 2.83 |
| Polyubiquitin-C | EHH24564.1 | 26 kDa | 6 | 4 | 5 | 1.41 |
| Cluster of histone H2A type 2-A (AFI33531.1) | AFI33531.1 | 14 kDa | 9 | 0 | 4.5 | 6.36 |
| Elongation factor 1-alpha 1 | NP_001182679.1 | 50 kDa | 5 | 4 | 4.5 | 0.71 |
| Laminin subunit gamma-1 precursor | AFJ71489.1 | 178 kDa | 5 | 4 | 4.5 | 0.71 |
| Elongation factor 2 | NP_001252725.1 | 95 kDa | 7 | 2 | 4.5 | 3.54 |
| Transforming growth factor-beta-induced protein ig-h3 precursor | AFH33796.1 | 75 kDa | 6 | 3 | 4.5 | 2.12 |
| PREDICTED: thymidine phosphorylase | XP_001112945.1 | 50 kDa | 9 | 0 | 4.5 | 6.36 |
| Cathepsin G preproprotein | AFH28827.1 | 29 kDa | 7 | 2 | 4.5 | 3.54 |
| Clathrin heavy chain 1 | AFE76626.1 | 147 kDa | 7 | 2 | 4.5 | 3.54 |
| Immunoglobulin heavy chain variable region, partial | ATV91288.1 | 9 kDa | 8 | 0 | 4 | 5.66 |
| Immunoglobulin light chain variable region, partial | APW29843.1 | 10 kDa | 0 | 8 | 4 | 5.66 |
| 78 kda glucose-regulated protein precursor | AFE63852.1 | 72 kDa | 8 | 0 | 4 | 5.66 |
| Histone H2A type 2-A | AFI33531.1 | 14 kDa | 8 | 0 | 4 | 5.66 |
| PREDICTED: ras-related protein Rab-10 | XP_014967350.1 | 23 kDa | 5 | 3 | 4 | 1.41 |
| Plastin-2 | NP_001253236.1 | 70 kDa | 8 | 0 | 4 | 5.66 |
| Interferon-induced GTP-binding protein Mx1 | AFI37198.1 | 75 kDa | 8 | 0 | 4 | 5.66 |
| Sulfhydryl oxidase 1 isoform a | AFH28885.1 | 82 kDa | 8 | 0 | 4 | 5.66 |
| Fatty acid synthase | AFE77136.1 | 273 kDa | 4 | 4 | 4 | 0 |
| Immunoglobulin kappa chain variable region, partial | ARW79875.1 | 10 kDa | 0 | 8 | 4 | 5.66 |
| Endoplasmin precursor | AFE79921.1 | 93 kDa | 8 | 0 | 4 | 5.66 |
| Mannosyl-oligosaccharide 1,2-alpha-mannosidase IA, partial | AFE70673.1 | 52 kDa | 6 | 2 | 4 | 2.83 |
| Rho-related GTP-binding protein rhoc precursor | AFE75896.1 | 22 kDa | 7 | 0 | 3.5 | 4.95 |
| PREDICTED: EH domain-containing protein 1 isoform X1 | XP_014969249.1 | 62 kDa | 5 | 2 | 3.5 | 2.12 |
| Integrin beta-1 isoform 1A precursor | AFH32300.1 | 88 kDa | 5 | 2 | 3.5 | 2.12 |
| Immunoglobulin light chain variable region, partial | APD72158.1 | 12 kDa | 0 | 6 | 3 | 4.24 |
| Cluster of 14-3-3 protein zeta/delta (NP_001247828.1) | NP_001247828.1 | 28 kDa | 6 | 0 | 3 | 4.24 |
| Cullin-associated NEDD8-dissociated protein 1 | NP_001248621.1 | 136 kDa | 6 | 0 | 3 | 4.24 |
| Monocyte differentiation antigen CD14 precursor | AFH30092.1 | 40 kDa | 4 | 2 | 3 | 1.41 |
| Rho-related GTP-binding protein rhog | EHH23200.1 | 21 kDa | 6 | 0 | 3 | 4.24 |
| Immunoglobulin lambda light chain, partial | AER46406.1 | 13 kDa | 0 | 5 | 2.5 | 3.54 |
| Ras-related protein Rab-1B | NP_001253093.1 | 22 kDa | 5 | 0 | 2.5 | 3.54 |
| Ras-related protein Rab-8B | NP_001244884.1 | 24 kDa | 5 | 0 | 2.5 | 3.54 |
| Cathepsin B preproprotein | AFI34702.1 | 38 kDa | 0 | 5 | 2.5 | 3.54 |
| Cluster of aldehyde dehydrogenase, mitochondrial isoform 1 precursor, partial (AFE70498.1) | AFE70498.1 | 55 kDa | 5 | 0 | 2.5 | 3.54 |
| 14-3-3 protein zeta/delta | NP_001247828.1 | 28 kDa | 5 | 0 | 2.5 | 3.54 |

| | | | | | | |
|---|---|---|---|---|---|---|
| PREDICTED: 14-3-3 protein epsilon isoform X1 | XP_014973666.1 | 29 kDa | 5 | 0 | 2.5 | 3.54 |
| ADP-ribosyl cyclase/cyclic ADP-ribose hydrolase 1 | NP_001248702.1 | 34 kDa | 5 | 0 | 2.5 | 3.54 |
| Cluster of L-lactate dehydrogenase A chain (NP_001244664.1) | NP_001244664.1 | 40 kDa | 3 | 2 | 2.5 | 0.71 |
| L-lactate dehydrogenase A chain | NP_001244664.1 | 40 kDa | 3 | 2 | 2.5 | 0.71 |
| PREDICTED: spectrin beta chain, erythrocytic isoform X1 | XP_014999378.1 | 268 kDa | 5 | 0 | 2.5 | 3.54 |
| Lipoprotein lipase | EHH28317.1 | 49 kDa | 5 | 0 | 2.5 | 3.54 |
| UDP-glcnac:betagal beta-1,3-N-acetylglucosaminyltransferase 7 | AFE67015.1 | 46 kDa | 5 | 0 | 2.5 | 3.54 |
| Cluster of alpha-actinin-1 isoform a, partial (AFE68656.1) | AFE68656.1 | 91 kDa | 5 | 0 | 2.5 | 3.54 |
| Alpha-actinin-1 isoform a, partial | AFE68656.1 | 91 kDa | 5 | 0 | 2.5 | 3.54 |
| Adipocyte plasma membrane-associated protein | NP_001252979.1 | 47 kDa | 5 | 0 | 2.5 | 3.54 |
| Low-density lipoprotein receptor isoform 3 precursor, partial | AFE73357.1 | 37 kDa | 0 | 5 | 2.5 | 3.54 |
| Olfactomedin-4 precursor, partial | AFE73522.1 | 19 kDa | 5 | 0 | 2.5 | 3.54 |
| Membrane-organizing extension spike protein, partial | EHH30801.1 | 67 kDa | 2 | 2 | 2 | 0 |
| PREDICTED: protein S100-A9 | XP_015008404.1 | 13 kDa | 0 | 4 | 2 | 2.83 |
| PREDICTED: retinal dehydrogenase 1 isoform X2 | XP_001097604.2 | 57 kDa | 4 | 0 | 2 | 2.83 |
| Hepatocyte-derived fibrinogen-related protein 1 | EHH28308.1 | 36 kDa | 0 | 4 | 2 | 2.83 |
| Cluster of actin-related protein 3B isoform 1 (AFH29197.1) | AFH29197.1 | 48 kDa | 4 | 0 | 2 | 2.83 |
| PREDICTED: actin-related protein 3 | XP_014965432.1 | 47 kDa | 4 | 0 | 2 | 2.83 |
| Actin-related protein 2/3 complex subunit 1B | AFH30667.1 | 41 kDa | 4 | 0 | 2 | 2.83 |
| PREDICTED: UTP--glucose-1-phosphate uridylyltransferase isoform X1 | XP_014967970.1 | 58 kDa | 4 | 0 | 2 | 2.83 |
| PREDICTED: coagulation factor XI isoform X1 | XP_014995138.1 | 70 kDa | 0 | 4 | 2 | 2.83 |
| 40S ribosomal protein SA | NP_001182409.1 | 33 kDa | 4 | 0 | 2 | 2.83 |
| 6-phosphogluconate dehydrogenase, decarboxylating | AFI34674.1 | 53 kDa | 4 | 0 | 2 | 2.83 |

**Supplementary Table 7.** Unique proteins to D0 EVs relative to D4 and D7 EVs.

| Identified Protein [Macaca mulatta] | Accession Number | Molecular Weight | Total Spectrum Counts | | | |
|---|---|---|---|---|---|---|
| | | | NHP 2401 D0 | NHP 2441 D0 | Average | Standard Deviation |
| Immunoglobulin kappa light chain, partial | ACN96923.1 | 21 kDa | 0 | 145 | 73 | 102.53 |
| Immunoglobulin heavy chain variable region, partial | AKE97596.1 | 13 kDa | 0 | 70 | 35.0 | 49.50 |
| Immunoglobulin heavy chain variable region, partial | AHN95047.1 | 13 kDa | 0 | 69 | 34.5 | 48.79 |
| PREDICTED: iggfc-binding protein | EHH30036.1/ XP_028694767.1 | 183 kDa | 31 | 33 | 32.0 | 1.41 |
| Immunoglobulin heavy chain variable region, partial | ATV91245.1 | 10 kDa | 62 | 0 | 31.0 | 43.84 |
| Immunoglobulin heavy chain variable region, partial | ATV91258.1 | 11 kDa | 59 | 0 | 29.5 | 41.72 |
| Anti-SIV env immunoglobulin ITS53 heavy chain variable region, partial | AMZ04037.1 | 12 kDa | 53 | 0 | 27 | 37.48 |
| Immunoglobulin heavy chain variable region, partial | AHN94658.1 | 13 kDa | 53 | 0 | 27 | 37.48 |
| Immunoglobulin heavy chain, partial | ACN96915.1 | 22 kDa | 41 | 0 | 20.5 | 28.99 |
| Immunoglobulin heavy chain variable region, partial | ANZ54740.1 | 11 kDa | 39 | 0 | 19.5 | 27.58 |
| Immunoglobulin heavy chain, partial | ACN96942.1 | 22 kDa | 0 | 30 | 15.0 | 21.21 |
| Immunoglobulin G light chain variable region, partial | AMS24778.1 | 12 kDa | 0 | 27 | 13.5 | 19.09 |
| Immunoglobulin heavy chain variable region, partial | AKE98177.1 | 14 kDa | 0 | 27 | 14 | 19.09 |
| Immunoglobulin heavy chain, partial | AAR83990.1 | 16 kDa | 26 | 0 | 13 | 18.38 |
| Immunoglobulin G heavy chain variable region, partial | AMS24931.1 | 13 kDa | 18 | 7 | 13 | 7.78 |
| Immunoglobulin kappa variable region, partial | AWN00198.1 | 12 kDa | 20 | 0 | 10 | 14.14 |
| Anti-SIV env immunoglobulin ITS16.01 light chain variable region, partial | AMZ04084.1 | 12 kDa | 19 | 0 | 10 | 13.44 |
| Immunoglobulin heavy chain variable region, partial | AHN95252.1 | 13 kDa | 0 | 19 | 10 | 13.44 |
| Immunoglobulin heavy chain variable region, partial | ARO71155.1 | 11 kDa | 0 | 15 | 7.5 | 10.61 |
| Anti-SIV env immunoglobulin ITS42 light chain variable region, partial | AMZ04098.1 | 11 kDa | 7 | 7 | 7.0 | 0.00 |
| Immunoglobulin light chain variable region, partial | AIN43896.1 | 12 kDa | 5 | 7 | 6 | 1.41 |
| Immunoglobulin heavy chain variable region, partial | AKE98020.1 | 13 kDa | 0 | 11 | 6 | 7.78 |
| Immunoglobulin heavy chain variable region, partial | ANZ54631.1 | 11 kDa | 0 | 10 | 5 | 7.07 |
| Immunoglobulin kappa light chain variable region, partial | ASR74653.1 | 12 kDa | 10 | 0 | 5.0 | 7.07 |
| Immunoglobulin light chain variable region, partial | APW29870.1 | 10 kDa | 6 | 4 | 5.0 | 1.41 |
| Anti-HIV immunoglobulin lambda chain variable region, partial | AME15461.1 | 12 kDa | 0 | 9 | 4.5 | 6.36 |
| Immunoglobulin kappa light chain, partial | AER46506.1 | 13 kDa | 0 | 9 | 5 | 6.36 |
| Immunoglobulin G heavy chain variable region, partial | AMS24937.1 | 13 kDa | 8 | 0 | 4 | 5.66 |
| Immunoglobulin G light chain variable region, partial | AMS25321.1 | 11 kDa | 0 | 8 | 4 | 5.66 |
| Immunoglobulin heavy chain variable region, partial | AKE97557.1 | 13 kDa | 0 | 8 | 4 | 5.66 |

| | | | | | | |
|---|---|---|---|---|---|---|
| Immunoglobulin heavy chain variable segment precursor, partial | AAF89366.1 | 13 kDa | 8 | 0 | 4 | 5.66 |
| Immunoglobulin kappa light chain variable region, partial | ASR74643.1 | 12 kDa | 5 | 3 | 4 | 1.41 |
| PREDICTED: multimerin-1 | XP_014994280.1 | 138 kDa | 5 | 3 | 4 | 1.41 |
| Immunoglobulin heavy chain variable region, partial | ATV91294.1 | 9 kDa | 0 | 7 | 3.5 | 4.95 |
| Immunoglobulin kappa chain variable region | EHH25606.1/ ATV91337.1 | 11 kDa | 7 | 0 | 3.5 | 4.95 |
| Immunoglobulin heavy chain, partial | AER46899.1 | 15 kDa | 0 | 6 | 3 | 4.24 |
| CD9 antigen | NP_001247590.1 (+1) | 25 kDa | 6 | 0 | 3 | 4.24 |
| Immunoglobulin heavy chain variable region, partial | AKE97878.1 | 14 kDa | 0 | 6 | 3 | 4.24 |
| Immunoglobulin G light chain variable region, partial | AMS24876.1 | 12 kDa | 0 | 5 | 2.5 | 3.54 |
| Immunoglobulin G heavy chain variable region, partial | AMS24982.1 | 13 kDa | 2 | 3 | 2.5 | 0.71 |
| Immunoglobulin heavy chain variable region, partial | ANZ54775.1 | 11 kDa | 0 | 5 | 2.5 | 3.54 |
| Immunoglobulin heavy chain variable region, partial | AHN94776.1 | 13 kDa | 0 | 5 | 2.5 | 3.54 |
| Immunoglobulin kappa light chain variable region, partial | APG53617.1 | 10 kDa | 0 | 5 | 2.5 | 3.54 |
| Immunoglobulin light chain variable region, partial | APW29808.1 | 10 kDa | 0 | 5 | 2.5 | 3.54 |
| Immunoglobulin G light chain variable region, partial | AMS25506.1 | 12 kDa | 4 | 0 | 2 | 2.83 |
| Immunoglobulin heavy chain variable region, partial | ATV91224.1 | 11 kDa | 4 | 0 | 2 | 2.83 |
| Immunoglobulin heavy chain variable region, partial | AIN43915.1 | 13 kDa | 0 | 4 | 2 | 2.83 |
| Immunoglobulin kappa chain variable region, partial | ATV91080.1 | 13 kDa | 4 | 0 | 2 | 2.83 |

**Supplementary Table 8.** Unique proteins to D4 EVs relative to D0 and D7 EVs.

| Identified Protein [Macaca mulatta] | Accession Number | Molecular Weight | Total Spectrum Counts | | | |
|---|---|---|---|---|---|---|
| | | | NHP 2401 D4 | NHP 2441 D4 | Average | Standard Deviation |
| Chain M, Light Chain Of Fab Of Rhesus Mab 2.5b | pdb\|3Q6G\|M | 22 kDa | 314 | 0 | 157 | 222.03 |
| Immunoglobulin lambda light chain, partial | ACN96972.1 | 22 kDa | 284 | 0 | 142.0 | 200.82 |
| Immunoglobulin lambda light chain, partial | ACN96977.1 | 21 kDa | 0 | 258 | 129.0 | 182.43 |
| Chain L, DH522UCA Fab fragment light chain | EHH20076.1/ 5UKN_L | 25 kDa | 143 | 0 | 71.5 | 101.12 |
| Immunoglobulin kappa light chain, partial | ACN96958.1 | 21 kDa | 0 | 121 | 60.5 | 85.56 |
| Immunoglobulin heavy chain, partial | AAR83983.1 | 16 kDa | 0 | 105 | 52.5 | 74.25 |
| Immunoglobulin heavy chain variable region, partial | ALW83516.1 | 13 kDa | 0 | 73 | 37 | 51.62 |
| Immunoglobulin G heavy chain variable region, partial | AMS25221.1 | 13 kDa | 67 | 0 | 34 | 47.38 |
| Chain H, DH522UCA Fab fragment heavy chain | pdb\|5UKN\|H | 24 kDa | 61 | 0 | 30.5 | 43.13 |
| Immunoglobulin lambda light chain, partial | AER46458.1 | 13 kDa | 48 | 0 | 24.0 | 33.94 |
| Immunoglobulin heavy chain, partial | AAS19722.1 | 16 kDa | 41 | 0 | 20.5 | 28.99 |
| Immunoglobulin lamba chain constant region | EHH20080.1/ ATV91011.1 | 11 kDa | 33 | 0 | 16.5 | 23.33 |
| Immunoglobulin lambda light chain, partial | AER46314.1 | 13 kDa | 0 | 30 | 15 | 21.21 |
| Immunoglobulin kappa light chain variable region, partial | APG53677.1 | 10 kDa | 0 | 28 | 14 | 19.80 |
| Immunoglobulin heavy chain variable region, partial | AKE97669.1 | 14 kDa | 27 | 0 | 14 | 19.09 |
| Membrane primary amine oxidase isoform X2 | EHH25010.1/ XP_001096495.2 | 85 kDa | 27 | 0 | 14 | 19.09 |
| Immunoglobulin heavy chain variable region, partial | ARO71081.1 | 13 kDa | 26 | 0 | 13 | 18.38 |
| Immunoglobulin heavy chain variable region, partial | ATV90971.1 | 13 kDa | 11 | 14 | 13 | 2.12 |
| Immunoglobulin heavy chain variable region, partial | AKE97478.1 | 13 kDa | 0 | 24 | 12.0 | 16.97 |
| Immunoglobulin heavy chain variable region, partial | AHN95164.1 | 13 kDa | 0 | 23 | 11.5 | 16.26 |
| Immunoglobulin kappa light chain variable region, partial | APG53655.1 | 10 kDa | 22 | 0 | 11 | 15.56 |
| Immunoglobulin G heavy chain variable region, partial | AMS25280.1 | 13 kDa | 0 | 19 | 10 | 13.44 |
| Immunoglobulin G light chain variable region, partial | AMS25079.1 | 12 kDa | 19 | 0 | 10 | 13.44 |
| Immunoglobulin heavy chain variable region, partial | AHN94873.1 | 13 kDa | 18 | 0 | 9.0 | 12.73 |
| Immunoglobulin heavy chain variable region, partial | AHN94355.1 | 13 kDa | 0 | 16 | 8.0 | 11.31 |
| Immunoglobulin heavy chain variable region, partial | AIN43907.1 | 13 kDa | 0 | 16 | 8.0 | 11.31 |
| Immunoglobulin heavy chain variable region, partial | AHN94759.1 | 13 kDa | 14 | 0 | 7 | 9.90 |
| Immunoglobulin heavy chain variable region, partial | AKC54351.1 | 10 kDa | 0 | 14 | 7 | 9.90 |
| Immunoglobulin G light chain variable region, partial | AMS24787.1 | 12 kDa | 7 | 6 | 7 | 0.71 |
| Immunoglobulin G light chain variable region, partial | AMS24779.1 | 12 kDa | 13 | 0 | 6.5 | 9.19 |
| Immunoglobulin G light chain variable region, partial | AMS24726.1 | 11 kDa | 0 | 13 | 6.5 | 9.19 |

| | | | | | | |
|---|---|---|---|---|---|---|
| Immunoglobulin heavy chain variable region, partial | ARO71170.1 | 11 kDa | 0 | 12 | 6 | 8.49 |
| Immunoglobulin heavy chain variable region, partial | AKE98054.1 | 13 kDa | 11 | 0 | 5.5 | 7.78 |
| Immunoglobulin G heavy chain variable region, partial | AMS25218.1 | 13 kDa | 10 | 0 | 5 | 7.07 |
| Immunoglobulin heavy chain variable region, partial | AHN95052.1 | 13 kDa | 9 | 0 | 4.5 | 6.36 |
| Immunoglobulin kappa light chain variable region, partial | APG53691.1 | 10 kDa | 0 | 9 | 4.5 | 6.36 |
| Immunoglobulin heavy chain variable region, partial | AKC54330.1 | 11 kDa | 0 | 8 | 4 | 5.66 |
| Immunoglobulin G light chain variable region, partial | AMS25505.1 | 12 kDa | 7 | 0 | 3.5 | 4.95 |
| Immunoglobulin G light chain variable region, partial | AMS25499.1 | 12 kDa | 7 | 0 | 3.5 | 4.95 |
| Immunoglobulin heavy chain variable region, partial | AHN94948.1 | 14 kDa | 4 | 3 | 3.5 | 0.71 |
| Immunoglobulin heavy chain variable region, partial | AKE97474.1 | 13 kDa | 0 | 7 | 3.5 | 4.95 |
| Immunoglobulin heavy chain variable region, partial | AHN94777.1 | 13 kDa | 6 | 0 | 3 | 4.24 |
| Immunoglobulin heavy chain variable region, partial | AHN94843.1 | 14 kDa | 6 | 0 | 3 | 4.24 |
| Immunoglobulin G light chain variable region, partial | AMS24740.1 | 12 kDa | 2 | 3 | 2.5 | 0.71 |
| Cytokeratin-6C | EHH20755.1 | 60 kDa | 5 | 0 | 2.5 | 3.54 |
| Immunoglobulin heavy chain variable region | EHH28237.1/ ATV90954.1 | 13 kDa | 5 | 0 | 2.5 | 3.54 |
| Immunoglobulin heavy chain variable region, partial | AAO43419.1 | 11 kDa | 0 | 5 | 2.5 | 3.54 |
| Immunoglobulin lamda light chain variable region, partial | ASR74637.1 | 12 kDa | 5 | 0 | 2.5 | 3.54 |
| Periostin | EHH28960.1 | 87 kDa | 2 | 3 | 2.5 | 0.71 |
| PREDICTED: coagulation factor XIII B chain isoform X1 | XP_001111017.1 | 76 kDa | 5 | 0 | 2.5 | 3.54 |
| Immunoglobulin G light chain variable region, partial | AMS24744.1 | 12 kDa | 0 | 4 | 2 | 2.83 |
| Immunoglobulin kappa light chain variable region, partial | APG53735.1 | 10 kDa | 4 | 0 | 2 | 2.83 |
| Cluster of immunoglobulin kappa light chain variable region, partial (APG53735.1) | APG53735.1 | 10 kDa | 4 | 0 | 2 | 2.83 |
| Cluster of immunoglobulin lambda chain variable region, partial (ATV91558.1) | ATV91558.1 | 10 kDa | 0 | 4 | 2 | 2.83 |
| Cytokeratin-5 | EHH20757.1 | 63 kDa | 0 | 4 | 2 | 2.83 |
| Immunoglobulin G light chain variable region, partial | AMS25399.1 | 11 kDa | 4 | 0 | 2 | 2.83 |
| Immunoglobulin G light chain variable region, partial | AMS25319.1 | 12 kDa | 4 | 0 | 2 | 2.83 |
| Immunoglobulin G light chain variable region, partial | AMS24744.1 | 12 kDa | 0 | 4 | 2 | 2.83 |
| Immunoglobulin G light chain variable region, partial | AMS25466.1 | 12 kDa | 4 | 0 | 2 | 2.83 |
| Immunoglobulin gamma variable region, partial | AWN00208.1 | 13 kDa | 0 | 4 | 2 | 2.83 |
| Immunoglobulin heavy chain variable region, partial | AHN94627.1 | 13 kDa | 0 | 4 | 2 | 2.83 |
| Immunoglobulin heavy chain variable region, partial | ANZ54616.1 | 11 kDa | 4 | 0 | 2 | 2.83 |
| Immunoglobulin heavy chain variable region, partial | ATV91124.1 | 13 kDa | 0 | 4 | 2 | 2.83 |
| Immunoglobulin heavy chain, partial | AAC02642.1 | 16 kDa | 0 | 4 | 2 | 2.83 |
| Immunoglobulin kappa chain variable region, partial | ATV91469.1 | 9 kDa | 0 | 4 | 2 | 2.83 |
| Immunoglobulin kappa light chain variable region, partial | APG53735.1 | 10 kDa | 4 | 0 | 2 | 2.83 |

| | | | | | | |
|---|---|---|---|---|---|---|
| Immunoglobulin kappa light chain, partial | AER46569.1 | 13 kDa | 4 | 0 | 2 | 2.83 |
| Pleckstrin | AFH29190.1 | 40 kDa | 0 | 4 | 2 | 2.83 |

**Supplementary Table 9.** Unique proteins to D7 EVs relative to D0 and D4 EVs.

| Identified Protein [Macaca mulatta] | Accession Number | Molecular Weight | Total Spectrum Counts | | | |
|---|---|---|---|---|---|---|
| | | | NHP 2401 D7 | NHP 2441 D7 | Average | Standard Deviation |
| PREDICTED: pentraxin-related protein PTX3 | XP_001103515.1 | 42 kDa | 58 | 61 | 60 | 2.12 |
| PREDICTED: lactotransferrin | XP_014986625.1 | 78 kDa | 45 | 60 | 52.5 | 10.61 |
| Heat shock protein 90kda alpha (cytosolic), class A member 1 | NP_001182596.1 | 85 kDa | 58 | 24 | 41.0 | 24.04 |
| Heat shock protein HSP 90-beta | NP_001182462.1 | 83 kDa | 57 | 19 | 38.0 | 26.87 |
| PREDICTED: versican core protein isoform X1 | XP_001112269.1 | 373 kDa | 26 | 45 | 35.5 | 13.44 |
| PREDICTED: versican core protein isoform X2 | XP_014995844.1 | 265 kDa | 26 | 45 | 35.5 | 13.44 |
| MHC class I protein | SOF04253.1 | 41 kDa | 60 | 0 | 30 | 42.43 |
| Heat shock cognate 71 kda protein | NP_001248586.1 | 71 kDa | 37 | 22 | 30 | 10.61 |
| MHC class I antigen, partial | ACA65899.1 | 40 kDa | 45 | 13 | 29.0 | 22.63 |
| MHC class I antigen, partial | ACF93206.1 | 39 kDa | 39 | 18 | 28.5 | 14.85 |
| Glyceraldehyde-3-phosphate dehydrogenase | NP_001182355.1 | 36 kDa | 34 | 22 | 28.0 | 8.49 |
| Leucine-rich alpha-2-glycoprotein | EHH29497.1 | 38 kDa | 28 | 28 | 28.0 | 0.00 |
| Pyruvate kinase isozymes M1/M2 isoform a | AFE78766.1 | 58 kDa | 29 | 20 | 25 | 6.36 |
| PREDICTED: ficolin-1 | XP_014971885.1 | 35 kDa | 19 | 27 | 23 | 5.66 |
| Histone H2B type 1-K, partial | AFE72303.1 | 14 kDa | 30 | 16 | 23 | 9.90 |
| Ferritin light chain | NP_001248136.1 | 20 kDa | 24 | 20 | 22 | 2.83 |
| MHC class I antigen, partial | ACI24721.1 | 31 kDa | 35 | 9 | 22 | 18.38 |
| Histone H2B type 2-E, partial | AFE72265.1 | 11 kDa | 29 | 14 | 22 | 10.61 |
| Anion exchange protein 1 | EHH25046.1 | 105 kDa | 32 | 5 | 18.5 | 19.09 |
| MHC class I antigen, partial | ABQ41415.1 | 39 kDa | 37 | 0 | 18.5 | 26.16 |
| PREDICTED: myeloperoxidase isoform X1 | XP_001103896.3 | 84 kDa | 20 | 13 | 17 | 4.95 |
| MHC class I antigen, partial | ABQ41418.1 | 39 kDa | 32 | 0 | 16 | 22.63 |
| Cluster of tubulin beta-2B chain (AFI36483.1) | AFI36483.1 [5] | 50 kDa | 20 | 11 | 16 | 6.36 |
| Tubulin beta-2B chain | AFI36483.1 (+4) | 50 kDa | 20 | 11 | 15.5 | 6.36 |
| Cluster of PREDICTED: histone H3.1-like (XP_002803695.2) | XP_002803695.2 | 28 kDa | 17 | 12 | 14.5 | 3.54 |
| PREDICTED: histone H3.1-like | XP_002803695.2 | 28 kDa | 17 | 12 | 14.5 | 3.54 |
| PREDICTED: amiloride-sensitive amine oxidase [copper-containing] | XP_014990764.1 | 86 kDa | 20 | 6 | 13 | 9.90 |
| Heat shock 70 kda protein 1/2 | EHH18181.1 | 70 kDa | 13 | 12 | 13 | 0.71 |
| PREDICTED: carbamoyl-phosphate synthase [ammonia], mitochondrial | XP_001110375.1 | 165 kDa | 6 | 17 | 12 | 7.78 |
| Guanine nucleotide-binding protein G(i) subunit alpha-2 isoform 1, partial | AFE71714.1 | 40 kDa | 16 | 7 | 11.5 | 6.36 |
| PREDICTED: neutrophil elastase | XP_014977795.1 | 28 kDa | 15 | 7 | 11 | 5.66 |
| Unc-112-related protein 2 | EHH22743.1 (+1) | 76 kDa | 17 | 4 | 10.5 | 9.19 |
| ADP-ribosylation factor 1 | NP_001180216.1 | 21 kDa | 15 | 5 | 10 | 7.07 |
| MHC class I antigen | ACR38990.1 | 41 kDa | 0 | 18 | 9 | 12.73 |
| Integrin beta-2 precursor | AFH32301.1 | 85 kDa | 10 | 7 | 8.5 | 2.12 |
| PREDICTED: receptor-type tyrosine-protein phosphatase C isoform X1 | XP_014976209.1 | 148 kDa | 14 | 3 | 8.5 | 7.78 |
| Cell division control protein 42 homolog | NP_001244809.1 | 21 kDa | 11 | 6 | 8.5 | 3.54 |
| Cytosol aminopeptidase | NP_001247627.1 | 56 kDa | 11 | 5 | 8 | 4.24 |
| Fc gamma receptor RII, partial | AEC03703.1 | 31 kDa | 8 | 8 | 8 | 0 |
| Olfactomedin-4 | EHH29036.1 | 57 kDa | 8 | 8 | 8 | 0 |
| Guanine nucleotide-binding protein G(I)/G(S)/G(T) subunit beta-1 | AFI33718.1 | 37 kDa | 11 | 4 | 7.5 | 4.95 |
| PREDICTED: integrin alpha-M isoform X1 | XP_014981504.1 | 128 kDa | 7 | 8 | 7.5 | 0.71 |
| Immunoglobulin kappa light chain variable region, partial | APG53699.1 | 11 kDa | 15 | 0 | 7.5 | 10.61 |

| | | | | | | |
|---|---|---|---|---|---|---|
| Immunoglobulin G heavy chain variable region, partial | AMS24956.1 | 13 kDa | 14 | 0 | 7 | 9.9 |
| Catalase | EHH22994.1 | 60 kDa | 14 | 0 | 7 | 9.9 |
| Immunoglobulin kappa chain variable region, partial | ATV91410.1 | 10 kDa | 0 | 14 | 7 | 9.9 |
| Nidogen-1 precursor | AFI34002.1 | 136 kDa | 6 | 7 | 6.5 | 0.71 |
| PREDICTED: fructose-bisphosphate aldolase A isoform X2 | XP_014981390.1 | 39 kDa | 9 | 4 | 6.5 | 3.54 |
| Aspartyl aminopeptidase | AFH29347.1 | 53 kDa | 8 | 4 | 6 | 2.83 |
| Vascular cell adhesion protein 1 isoform a precursor | AFH33883.1 | 81 kDa | 8 | 4 | 6 | 2.83 |
| Platelet glycoprotein 4 | NP_001028085.1 | 53 kDa | 7 | 5 | 6 | 1.41 |
| MHC class I antigen, partial | AFV31657.1 | 41 kDa | 0 | 12 | 6 | 8.49 |
| Alcohol dehydrogenase 1B | NP_001247556.1 | 40 kDa | 7 | 5 | 6 | 1.41 |
| PREDICTED: transforming protein rhoa | XP_014986395.1 | 22 kDa | 8 | 4 | 6 | 2.83 |
| Aspartyl aminopeptidase | AFH29347.1 | 53 kDa | 8 | 4 | 6 | 2.83 |
| Tenascin | EHH23833.1 (+2) | 241 kDa | 7 | 4 | 5.5 | 2.12 |
| Polyubiquitin-C | EHH24564.1 | 26 kDa | 6 | 4 | 5 | 1.41 |
| Ras-related protein Rab-5C | NP_001253100.1 | 23 kDa | 7 | 3 | 5 | 2.83 |
| Ras-related C3 botulinum toxin substrate 2 | NP_001248306.1 | 21 kDa | 6 | 4 | 5 | 1.41 |
| Polyubiquitin-C | EHH24564.1 | 26 kDa | 6 | 4 | 5 | 1.41 |
| Elongation factor 1-alpha 1 | NP_001182679.1 | 50 kDa | 5 | 4 | 4.5 | 0.71 |
| Laminin subunit gamma-1 precursor | AFJ71489.1 | 178 kDa | 5 | 4 | 4.5 | 0.71 |
| Elongation factor 2 | NP_001252725.1 | 95 kDa | 7 | 2 | 4.5 | 3.54 |
| Transforming growth factor-beta-induced protein ig-h3 precursor | AFH33796.1 | 75 kDa | 6 | 3 | 4.5 | 2.12 |
| Cathepsin G preproprotein | AFH28827.1 | 29 kDa | 7 | 2 | 4.5 | 3.54 |
| PREDICTED: thymidine phosphorylase | XP_001112945.1 | 50 kDa | 9 | 0 | 4.5 | 6.36 |
| Clathrin heavy chain 1 | AFE76626.1 | 147 kDa | 7 | 2 | 4.5 | 3.54 |
| Plastin-2 | NP_001253236.1 | 70 kDa | 8 | 0 | 4 | 5.66 |
| Interferon-induced GTP-binding protein Mx1 | AFI37198.1 | 75 kDa | 8 | 0 | 4 | 5.66 |
| Histone H2A type 2-A | AFI33531.1 | 14 kDa | 8 | 0 | 4 | 5.66 |
| Mannosyl-oligosaccharide 1,2-alpha-mannosidase IA, partial | AFE70673.1 | 52 kDa | 6 | 2 | 4 | 2.83 |
| Sulfhydryl oxidase 1 isoform a | AFH28885.1 | 82 kDa | 8 | 0 | 4 | 5.66 |
| Fatty acid synthase | AFE77136.1 | 273 kDa | 4 | 4 | 4 | 0 |
| Immunoglobulin kappa chain variable region, partial | ARW79875.1 | 10 kDa | 0 | 8 | 4 | 5.66 |
| PREDICTED: ras-related protein Rab-10 | XP_014967350.1 | 23 kDa | 5 | 3 | 4 | 1.41 |
| Endoplasmin precursor | AFE79921.1 | 93 kDa | 8 | 0 | 4 | 5.66 |
| Immunoglobulin light chain variable region, partial | APW29843.1 | 10 kDa | 0 | 8 | 4 | 5.66 |
| Immunoglobulin heavy chain variable region, partial | ATV91288.1 | 9 kDa | 8 | 0 | 4 | 5.66 |
| 78 kda glucose-regulated protein precursor | AFE63852.1 | 72 kDa | 8 | 0 | 4 | 5.66 |
| Immunoglobulin G light chain variable region, partial | AMS25138.1 | 11 kDa | 7 | 0 | 3.5 | 4.95 |
| PREDICTED: EH domain-containing protein 1 isoform X1 | XP_014969249.1 | 62 kDa | 5 | 2 | 3.5 | 2.12 |
| Rho-related GTP-binding protein rhoc precursor | AFE75896.1 | 22 kDa | 7 | 0 | 3.5 | 4.95 |
| Integrin beta-1 isoform 1A precursor | AFH32300.1 (+3) | 88 kDa | 5 | 2 | 3.5 | 2.12 |
| Cullin-associated NEDD8-dissociated protein 1 | NP_001248621.1 | 136 kDa | 6 | 0 | 3 | 4.24 |
| Monocyte differentiation antigen CD14 precursor | AFH30092.1 | 40 kDa | 4 | 2 | 3 | 1.41 |
| Rho-related GTP-binding protein rhog | EHH23200.1 | 21 kDa | 6 | 0 | 3 | 4.24 |

| | | | | | | |
|---|---|---|---|---|---|---|
| Immunoglobulin light chain variable region, partial | APD72158.1 | 12 kDa | 0 | 6 | 3 | 4.24 |
| Immunoglobulin lambda light chain, partial | AER46406.1 | 13 kDa | 0 | 5 | 2.5 | 3.54 |
| Adipocyte plasma membrane-associated protein | NP_001252979.1 | 47 kDa | 5 | 0 | 2.5 | 3.54 |
| Cathepsin B preproprotein | AFI34702.1 | 38 kDa | 0 | 5 | 2.5 | 3.54 |
| L-lactate dehydrogenase A chain | NP_001244664.1 | 40 kDa | 3 | 2 | 2.5 | 0.71 |
| Low-density lipoprotein receptor isoform 3 precursor, partial | AFE73357.1 | 37 kDa | 0 | 5 | 2.5 | 3.54 |
| ADP-ribosyl cyclase/cyclic ADP-ribose hydrolase 1 | NP_001248702.1 | 34 kDa | 5 | 0 | 2.5 | 3.54 |
| Ras-related protein Rab-1B | NP_001253093.1 | 22 kDa | 5 | 0 | 2.5 | 3.54 |
| PREDICTED: spectrin beta chain, erythrocytic isoform X1 | XP_014999378.1 | 268 kDa | 5 | 0 | 2.5 | 3.54 |
| Lipoprotein lipase | EHH28317.1 | 49 kDa | 5 | 0 | 2.5 | 3.54 |
| 14-3-3 protein zeta/delta | NP_001247828.1 | 28 kDa | 5 | 0 | 2.5 | 3.54 |
| UDP-glcnac:betagal beta-1,3-N-acetylglucosaminyltransferase 7 | AFE67015.1 | 46 kDa | 5 | 0 | 2.5 | 3.54 |
| Immunoglobulin G heavy chain variable region, partial | AMS24988.1 | 13 kDa | 5 | 0 | 2.5 | 3.54 |
| Ras-related protein Rab-8B | NP_001244884.1 | 24 kDa | 5 | 0 | 2.5 | 3.54 |
| PREDICTED: 14-3-3 protein epsilon isoform X1 | XP_014973666.1 | 29 kDa | 5 | 0 | 2.5 | 3.54 |
| Cluster of alpha-actinin-1 isoform a, partial (AFE68656.1) | AFE68656.1 [6] | 91 kDa | 5 | 0 | 2.5 | 3.54 |
| Alpha-actinin-1 isoform a, partial | AFE68656.1 (+5) | 91 kDa | 5 | 0 | 2.5 | 3.54 |
| Olfactomedin-4 precursor, partial | AFE73522.1 | 19 kDa | 5 | 0 | 2.5 | 3.54 |
| Immunoglobulin G light chain variable region, partial | AMS25103.1 | 12 kDa | 0 | 4 | 2 | 2.83 |
| Membrane-organizing extension spike protein, partial | EHH30801.1 | 67 kDa | 2 | 2 | 2 | 0 |
| PREDICTED: protein S100-A9 | XP_015008404.1 | 13 kDa | 0 | 4 | 2 | 2.83 |
| Hepatocyte-derived fibrinogen-related protein 1 | EHH28308.1 | 36 kDa | 0 | 4 | 2 | 2.83 |
| Actin-related protein 2/3 complex subunit 1B | AFH30667.1 | 41 kDa | 4 | 0 | 2 | 2.83 |
| PREDICTED: UTP--glucose-1-phosphate uridylyltransferase isoform X1 | XP_014967970.1 | 58 kDa | 4 | 0 | 2 | 2.83 |
| PREDICTED: coagulation factor XI isoform X1 | XP_014995138.1 | 70 kDa | 0 | 4 | 2 | 2.83 |
| 40S ribosomal protein SA | NP_001182409.1 | 33 kDa | 4 | 0 | 2 | 2.83 |
| 6-phosphogluconate dehydrogenase, decarboxylating | AFI34674.1 | 53 kDa | 4 | 0 | 2 | 2.83 |
| PREDICTED: actin-related protein 3 | XP_014965432.1 | 47 kDa | 4 | 0 | 2 | 2.83 |
| PREDICTED: retinal dehydrogenase 1 isoform X2 | XP_001097604.2 | 57 kDa | 4 | 0 | 2 | 2.83 |

**Supplementary Table 10.** Differentially expressed proteins in D0, D4 and D7. Upregulated proteins in each group are shaded.

| Identified Protein [Macaca mulatta] | Accession Number | MW | D0 Total Spectral Counts | | | | D4 Total Spectral Counts | | | | D7 Total Spectral Counts | | | |
|---|---|---|---|---|---|---|---|---|---|---|---|---|---|---|
| | | | NHP 2401 | NHP 2441 | Av. | St. Dev | NHP 2401 | NHP 2441 | Av. | St. Dev | NHP 2401 | NHP 2441 | Av. | St. Dev |
| PREDICTED: thrombospondin-1 | XP_014997402.1 | 129 kDa | 255 | 212 | 234 | 30.4 | 121 | 158 | 140 | 26.2 | 25 | 2 | 14 | 16.3 |
| PREDICTED: CD5 antigen-like | XP_001116945.1 | 38 kDa | 105 | 92 | 98.5 | 9.19 | 81 | 72 | 76.5 | 6.36 | 52 | 49 | 50.5 | 2.12 |
| PREDICTED: iggfc-binding protein | XP_014979309.1 | 391 kDa | 48 | 50 | 49.0 | 1.41 | 17 | 36 | 26.5 | 13.4 | 15 | 8 | 11.5 | 4.95 |
| Extracellular matrix protein 1 isoform 1 precursor | AFE67144.1 | 61 kDa | 12 | 12 | 12.0 | 0.00 | 6 | 4 | 5.0 | 1.41 | 2 | 4 | 3.0 | 1.41 |
| CD5 antigen-like | EHH15372.1/ XP_001116945.2 | 38 kDa | 86 | 86 | 86.0 | 0.00 | 77 | 68 | 72.5 | 6.36 | 50 | 47 | 48.5 | 2.12 |
| PREDICTED: iggfc-binding protein | EHH30036.1/ XP_028694767.1 | 183 kDa | 31 | 33 | 32.0 | 1.41 | 0 | 0 | 0.0 | 0.00 | 0 | 0 | 0.0 | 0.00 |
| PREDICTED: cholesteryl ester transfer protein isoform X1 | XP_014981711.1 | 58 kDa | 6 | 10 | 8 | 2.83 | 18 | 11 | 15 | 4.95 | 4 | 5 | 5 | 0.71 |
| Immunoglobulin heavy chain variable region, partial | ATV90971.1 | 13 kDa | 0 | 0 | 0 | 0.00 | 11 | 14 | 13 | 2.12 | 0 | 0 | 0 | 0.00 |
| Clusterin precursor | NP_001182403.1 | 52 kDa | 67 | 69 | 68.0 | 1.41 | 110 | 115 | 113 | 3.54 | 196 | 175 | 185 | 14.9 |
| Beta-1B-glycoprotein | EHH23143.1 | 52 kDa | 15 | 13 | 14.0 | 1.41 | 88 | 60 | 74.0 | 19.8 | 159 | 168 | 164 | 6.36 |
| PREDICTED: fibrinogen beta chain | XP_014994859.1 | 56 kDa | 15 | 18 | 16.5 | 2.12 | 6 | 13 | 9.5 | 4.95 | 245 | 159 | 202 | 60.8 |
| PREDICTED: fibrinogen gamma chain isoform X1 | XP_001089651.1 | 52 kDa | 8 | 18 | 13.0 | 7.07 | 13 | 16 | 14.5 | 2.12 | 194 | 113 | 154 | 57.3 |
| TPA: globin A1 | SAI82170.1 | 16 kDa | 9 | 7 | 8 | 1.41 | 14 | 19 | 17 | 3.54 | 81 | 57 | 69 | 17.0 |
| Ferritin heavy chain | NP_001182309.1 | 21 kDa | 14 | 0 | 7 | 9.90 | 3 | 0 | 2 | 2.12 | 77 | 53 | 65 | 17.0 |
| PREDICTED: complement C2 isoform X1 | XP_014991516.1 | 83 kDa | 2 | 0 | 1 | 1.41 | 13 | 8 | 11 | 3.54 | 46 | 40 | 43 | 4.24 |
| PREDICTED: pentraxin-related protein PTX3 | XP_001103515.1 | 42 kDa | 0 | 0 | 0 | 0.00 | 0 | 0 | 0 | 0.00 | 58 | 61 | 60 | 2.12 |
| PREDICTED: lactotransferrin | XP_014986625.1 | 78 kDa | 0 | 0 | 0 | 0.00 | 0 | 0 | 0 | 0.00 | 45 | 60 | 53 | 10.6 |
| PREDICTED: proteoglycan 4 isoform X4 | XP_014977337.1 | 132 kDa | 4 | 4 | 4 | 0.00 | 3 | 0 | 2 | 2.12 | 19 | 27 | 23 | 5.66 |
| Recname: Full=Amyloid protein A; altname: Full=Amyloid fibril protein AA | SAA_MACMU | 9 kDa | 0 | 0 | 0.0 | 0.00 | 12 | 0 | 6.0 | 8.49 | 47 | 33 | 40.0 | 9.90 |
| Ferritin light chain | NP_001248136.1 | 20 kDa | 0 | 0 | 0.0 | 0.00 | 0 | 0 | 0.0 | 0.00 | 24 | 20 | 22.0 | 2.83 |
| PREDICTED: fibrinogen alpha chain | XP_014994860.1 | 73 kDa | 9 | 18 | 14 | 6.36 | 25 | 30 | 28 | 3.54 | 207 | 184 | 196 | 16.3 |
| PREDICTED: carboxypeptidase | XP_015003402.1 | 52 kDa | 0 | 0 | 0 | 0.00 | 3 | 4 | 4 | 0.71 | 8 | 10 | 9 | 1.41 |

| | | | | | | | | | | | | | |
|---|---|---|---|---|---|---|---|---|---|---|---|---|---|
| N catalytic chain isoform X1 | | | | | | | | | | | | | |
| Glyceraldehyde-3-phosphate dehydrogenase | NP_001182355.1 | 36 kDa | 0 | 0 | 0 | 0.00 | 0 | 0 | 0 | 0.00 | 34 | 22 | 28 | 8.49 |
| PREDICTED: myeloperoxidase isoform X1 | XP_001103896.3 | 84 kDa | 0 | 0 | 0.0 | 0.00 | 0 | 0 | 0.0 | 0.00 | 20 | 13 | 16.5 | 4.95 |
| Pyruvate kinase isozymes M1/M2 isoform a | AFE78766.1 | 58 kDa | 0 | 0 | 0.0 | 0.00 | 0 | 0 | 0.0 | 0.00 | 29 | 20 | 24.5 | 6.36 |
| Amyloid protein A-like | EHH23059.1/ XP_001086242.2 | 17 kDa | 0 | 0 | 0.0 | 0.00 | 9 | 0 | 4.5 | 6.36 | 34 | 22 | 28.0 | 8.49 |
| PREDICTED: histone H3.1-like | XP_002803695.2 | 28 kDa | 0 | 0 | 0 | 0.00 | 0 | 0 | 0 | 0.00 | 17 | 12 | 15 | 3.54 |
| Ras-related protein Rap-1b | NP_001185607.1 | 21 kDa | 4 | 3 | 4 | 0.71 | 0 | 0 | 0 | 0.00 | 12 | 9 | 11 | 2.12 |
| Heat shock cognate 71 kda protein | NP_001248586.1 | 71 kDa | 0 | 0 | 0 | 0.00 | 0 | 0 | 0 | 0.00 | 37 | 22 | 30 | 10.6 |
| Integrin beta-2 precursor | AFH32301.1 | 85 kDa | 0 | 0 | 0 | 0 | 0 | 0 | 0 | 0 | 10 | 7 | 8.5 | 2.12 |
| Membrane-organizing extension spike protein, partial | EHH30801.1 | 67 kDa | 0 | 0 | 0 | 0 | 0 | 0 | 0 | 0 | 2 | 2 | 2 | 0 |
| Fc gamma receptor RII, partial | AEC03703.1 | 31 kDa | 0 | 0 | 0 | 0 | 0 | 0 | 0 | 0 | 8 | 8 | 8 | 0 |
| Cell division control protein 42 homolog | NP_001244809.1 | 21 kDa | 0 | 0 | 0 | 0 | 0 | 0 | 0 | 0 | 11 | 6 | 8.5 | 3.54 |
| Platelet glycoprotein 4 | NP_001028085.1 | 53 kDa | 0 | 0 | 0 | 0 | 0 | 0 | 0 | 0 | 7 | 5 | 6 | 1.41 |
| Elongation factor 1-alpha 1 | NP_001182679.1 | 50 kDa | 0 | 0 | 0 | 0 | 0 | 0 | 0 | 0 | 5 | 4 | 4.5 | 0.71 |
| PREDICTED: integrin alpha-M isoform X1 | XP_014981504.1 | 128 kDa | 0 | 0 | 0 | 0 | 0 | 0 | 0 | 0 | 7 | 8 | 7.5 | 0.71 |
| Heat shock 70 kda protein 1/2 | EHH18181.1 | 70 kDa | 0 | 0 | 0 | 0 | 0 | 0 | 0 | 0 | 13 | 12 | 12.5 | 0.71 |
| Laminin subunit gamma-1 precursor | AFJ71489.1 | 178 kDa | 0 | 0 | 0 | 0 | 0 | 0 | 0 | 0 | 5 | 4 | 4.5 | 0.71 |
| L-lactate dehydrogenase A chain | NP_001244664.1 | 40 kDa | 0 | 0 | 0 | 0 | 0 | 0 | 0 | 0 | 3 | 2 | 2.5 | 0.71 |
| Fatty acid synthase | AFE77136.1 | 273 kDa | 0 | 0 | 0 | 0 | 0 | 0 | 0 | 0 | 4 | 4 | 4 | 0 |
| Ras-related C3 botulinum toxin substrate 2 | NP_001248306.1 | 21 kDa | 0 | 0 | 0 | 0 | 0 | 0 | 0 | 0 | 6 | 4 | 5 | 1.41 |
| PREDICTED: leucine-rich alpha-2-glycoprotein isoform X1 | XP_001082565.2 | 42 kDa | 0 | 0 | 0 | 0 | 6 | 4 | 5 | 1.41 | 28 | 30 | 29 | 1.41 |
| PREDICTED: ras-related protein Rab-10 | XP_014967350.1 | 23 kDa | 0 | 0 | 0 | 0 | 0 | 0 | 0 | 0 | 5 | 3 | 4 | 1.41 |
| Leucine-rich alpha-2-glycoprotein | EHH29497.1 | 38 kDa | 0 | 0 | 0 | 0 | 0 | 0 | 0 | 0 | 28 | 28 | 28 | 0 |
| Alcohol dehydrogenase 1B | NP_001247556.1 | 40 kDa | 0 | 0 | 0 | 0 | 0 | 0 | 0 | 0 | 7 | 5 | 6 | 1.41 |
| Fibrinogen alpha chain isoform X2 | EHH26257.1/ XP_028704661.1 | 92 kDa | 0 | 0 | 0 | 0 | 0 | 25 | 12.5 | 17.7 | 186 | 168 | 177 | 12.7 |

**Supplementary Figures**

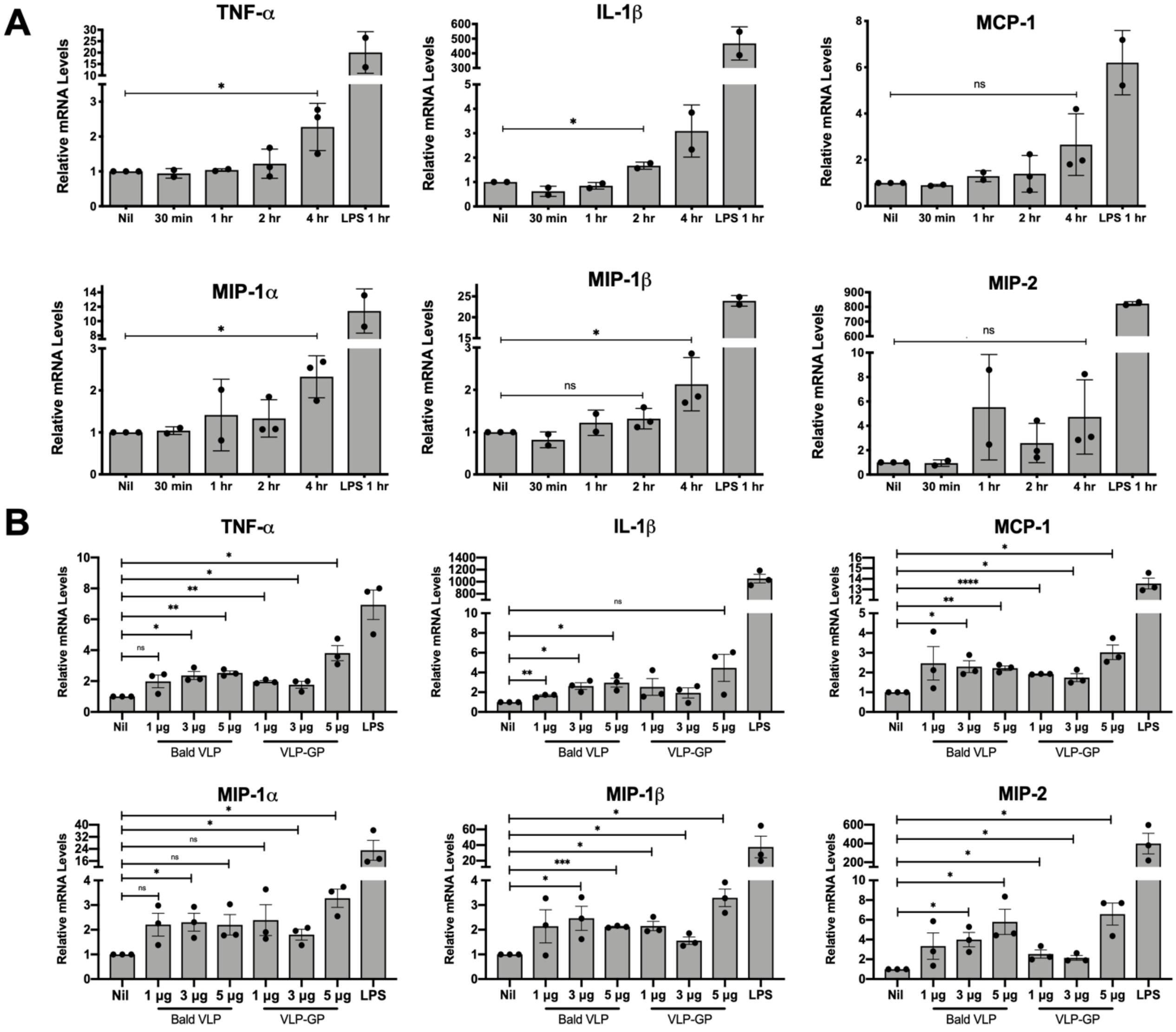


**Supplementary Figure 1. Pro-inflammatory mediators are differentially expressed *in vitro* in B10R macrophages over stimulation time course and in response to varying concentrations of EBOV VLPs. (A)** Pro-inflammatory cytokine and chemokine mRNA levels in B10R macrophages following stimulation with Bald VLP (5 µg/mL) for increasing lengths of time (30 minutes, 1, 2, or 4 hours) or LPS (100 ng/mL) for 1 hour, as measured by qRT-PCR. The unstimulated state (Nil) is shown for reference. 18S rRNA was used as a normalization gene. Data are shown as mean ± SD and each point represents one experiment, performed in duplicate ($n$ = 2 or 3, one-tailed unpaired t-test with Welch's correction, *$p < 0.05$). Statistical significance is measured against the Nil. **(B)** Pro-inflammatory cytokine and chemokine mRNA levels in B10R macrophages following stimulation with

Bald VLP or VLP-GP at increasing concentrations (1, 3, or 5 µg/mL) or LPS (100 ng/mL) for 4 hours, as measured by qRT-PCR. The unstimulated state (Nil) is shown for reference. 18S rRNA was used as a normalization gene. Data are shown as mean ± SEM and each point represents one experiment, performed in duplicate ($n$ = 3, one-tailed unpaired t-test with Welch's correction, *$p < 0.05$; **$p < 0.01$; ***$p < 0.001$; ****$p < 0.0001$). Statistical significance is measured against the Nil.

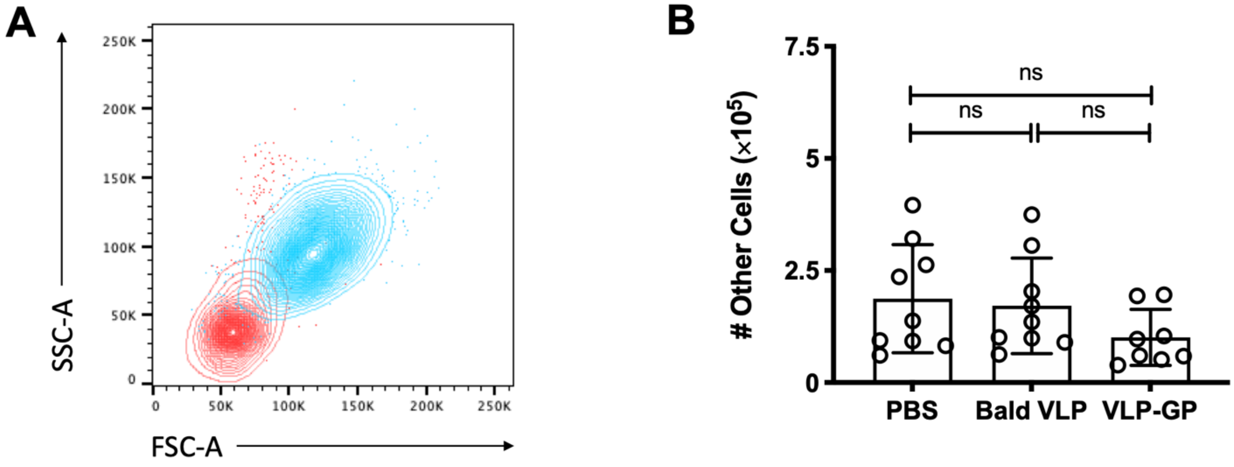


**Supplementary Figure 2. Flow cytometry results. (A)** Forward versus side scatter (FSC vs. SSC) gating plot to distinguish small (red, lymphoid$^{neg}$F4/80$^{lo}$CD11b$^{+}$) and large (blue, lymphoid$^{neg}$F4/80$^{hi}$CD11b$^{+}$) peritoneal macrophages. **(B)** Other cells (negative for all stains) present in peritoneal cavity (5 mL) do not account for any differences between the experimental groups ($n$ = 9 mice per group, one-way ANOVA with Tukey's correction for multiple comparisons, ns: not significant).

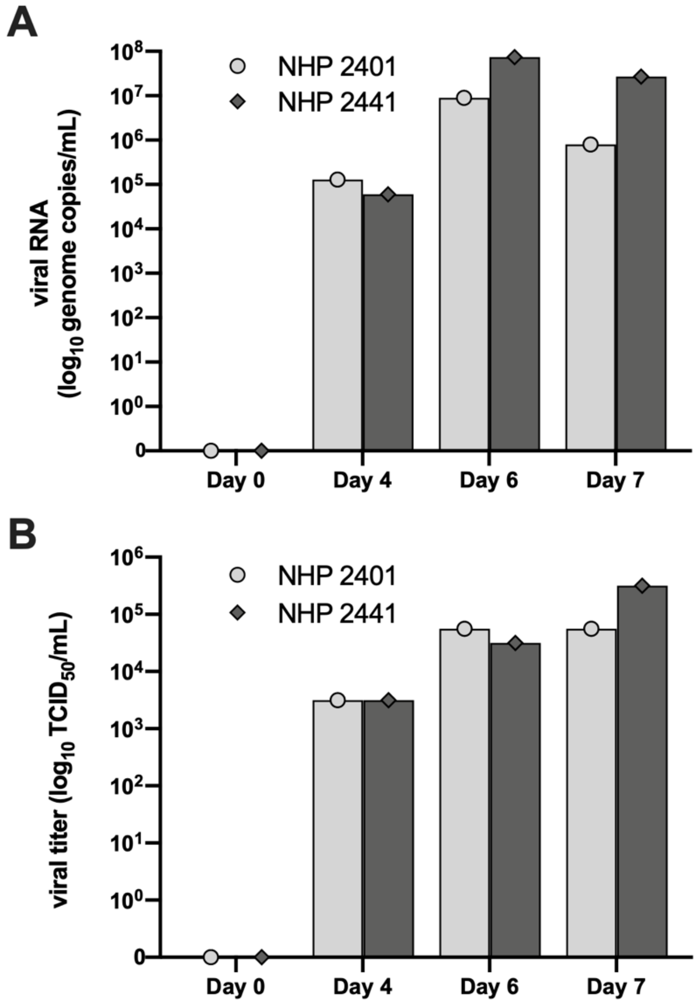


**Supplementary Figure 3. Levels of viremia in EBOV-infected rhesus macaques over infection time course. (A)** Viral RNA (genome copies/mL) and **(B)** viral titers ($TCID_{50}$/mL) in blood samples from rhesus macaques infected with 1000 $TCID_{50}$ of EBOV Makona C07. Samples were collected on day 0 (pre-infection), and days 4, 6, and 7 post-infection, and measured by qRT-PCR and $TCID_{50}$ assay, respectively ($n$ = 2). Values are expressed on a logarithmic scale.

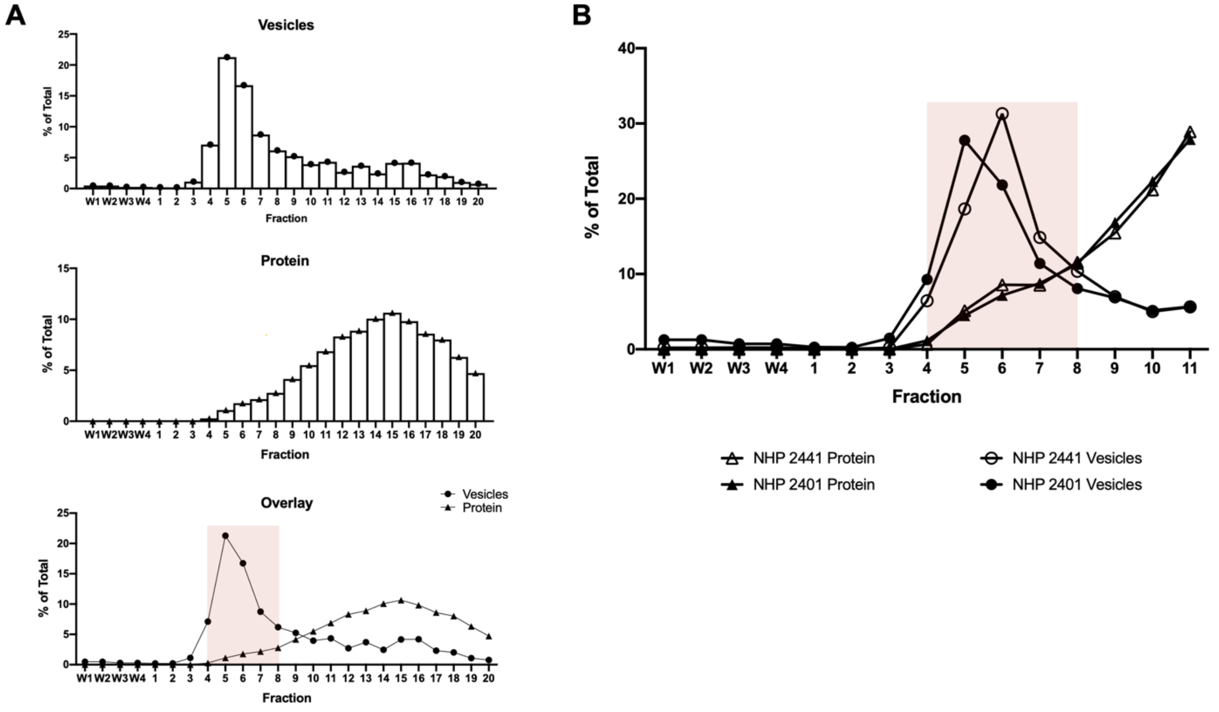


**Supplementary Figure 4. Serum-derived extracellular vesicles from EBOV-infected rhesus macaques elute from SEC column first in early fractions, followed by proteins. (A)** Example of the SEC elution profiles of a 10-mL SEC column loaded with serum from NHP 2401 6 days post-infection for vesicles (left panel), proteins (middle panel), and both (right panel), as measured by NTA, micro-BCA protein assay, and both, respectively. Waste fractions (W1-W4) were collected in volumes of 1 mL and subsequent fractions (1-20) were collected in volumes of 500 µL. Each data point shows the number present in a fraction as % of the total number that eluted from the column (*n* = 1). EVs eluted in fractions 4-8 (shaded area), while proteins eluted later in fractions 9-20. **(B)** Representative SEC elution profile of EVs and total proteins isolated from serum from NHP 2401 and 2441 6 days post-infection, as measured by NTA and micro-BCA protein assay. W1-W4 were collected in volumes of 1 mL and fractions 1-11 were collected in volumes of 500 µL. Each data point shows the number present in a fraction as % of the total number that eluted from the column (*n* = 2).

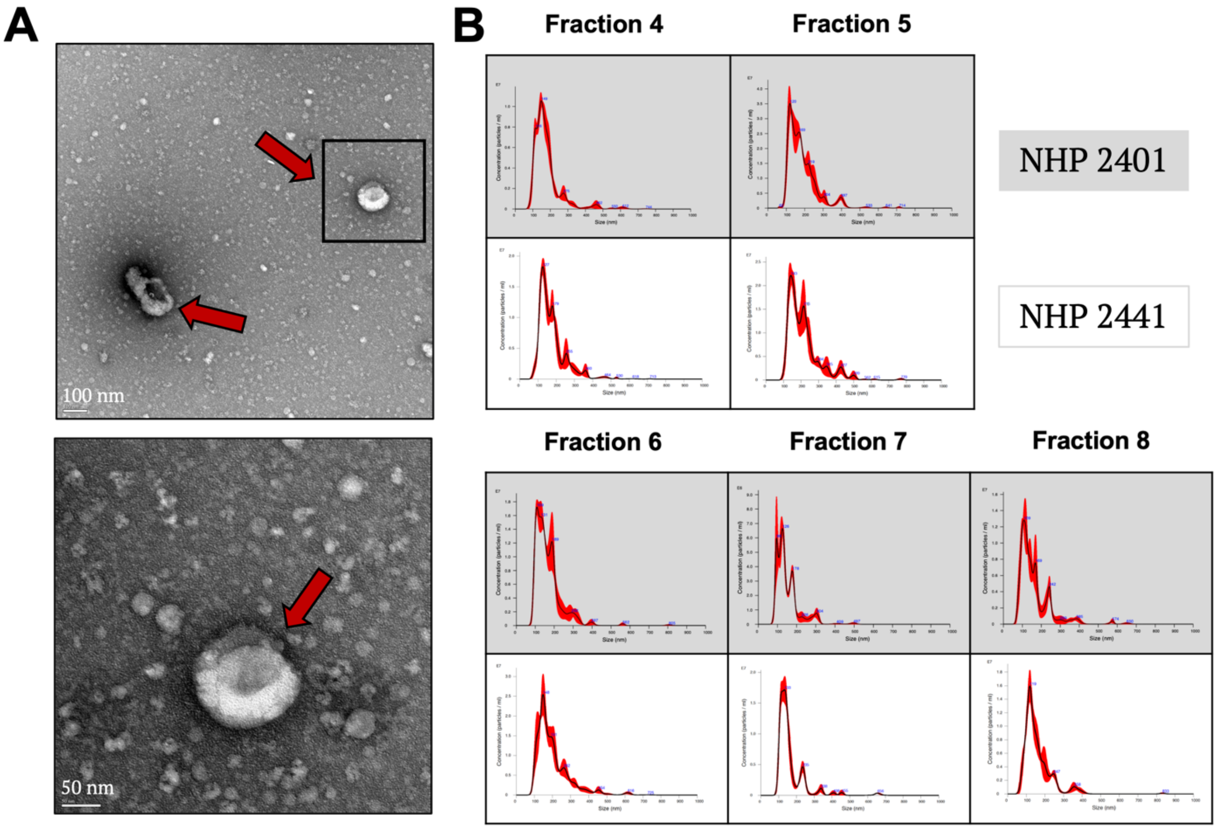


**Supplementary Figure 5. Isolation of serum-derived extracellular vesicles from EBOV-infected rhesus macaques by SEC. (A)** EVs (red arrows) in pooled fractions 4-8 isolated from SEC column loaded with serum from NHP 2401 6 days post-infection were prepared for TEM by negative staining with uranyl acetate to reveal the ultrastructure ($n$ = 1). Images are representative of transmission electron micrographs from 12 independent SEC serum-derived EV isolations. TEM magnifications: 30,000x (left panel) and 98,000x (right panel). **(B)** Graphical representation of particle distributions (concentration of estimated sizes) among fractions 4-8 eluted from SEC column loaded with serum from NHP 2401 and 2441 7 days post-infection, as generated by the NanoSight NS500 platform. Graphs represent mean ± SEM (red) of 3 separate NanoSight videos recorded per fraction ($n$ = 2). Results are representative of NTA data from 12 independent SEC serum-derived EV isolations.

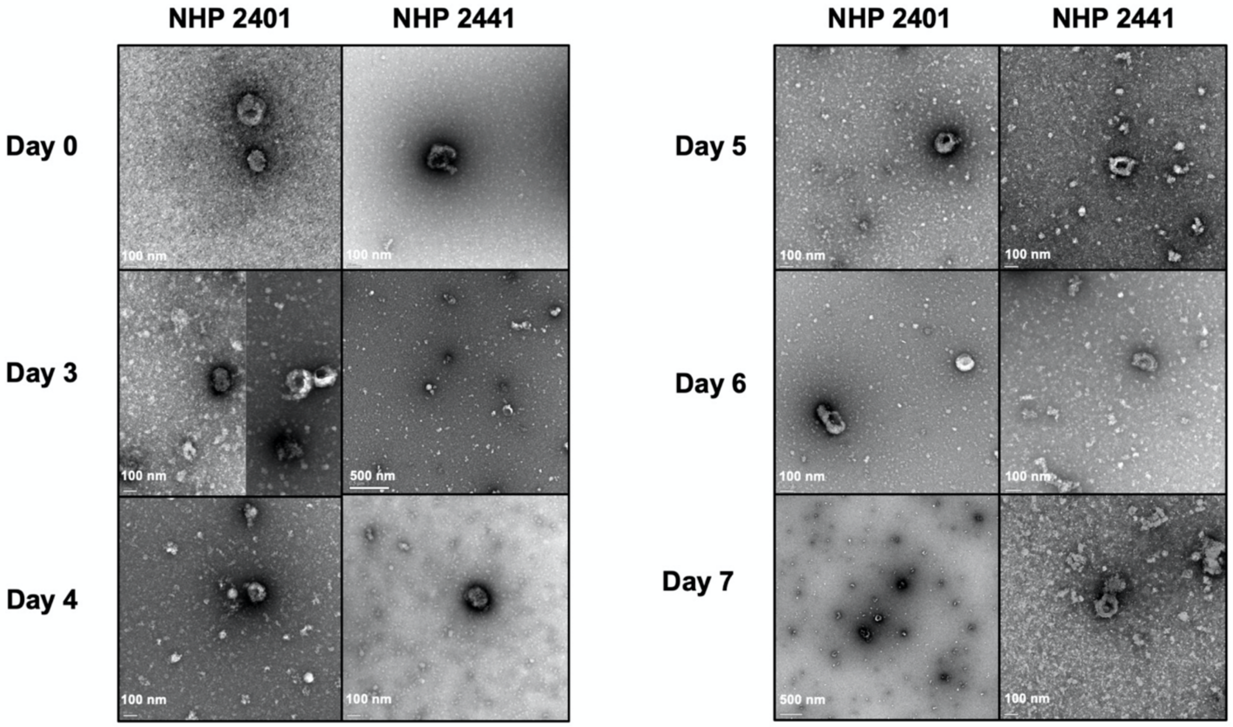


**Supplementary Figure 6. Transmission electron micrographs of serum-derived extracellular vesicles from EBOV-infected rhesus macaques by SEC.** EVs in pooled fractions 4-8 isolated from SEC column loaded with serum from NHP 2401 and NHP 2441 collected on day 0 (pre-infection) and days 3, 4, 5, 6, and 7 post-infection and prepared for TEM by negative staining with uranyl acetate to reveal the ultrastructure (*n* = 2). TEM magnifications for NHP 2401: D0 (30,00x), D3 (30,000x), D4 (30,000x), D5 (30,000x), D6 (30,000x), and D7 (9,300x). TEM magnifications for NHP 2441: D0 (30,000x), D3 (9,300x), D4 (30,000x), D5 (30,000x), D6 (30,000x), and D7 (30,000x).

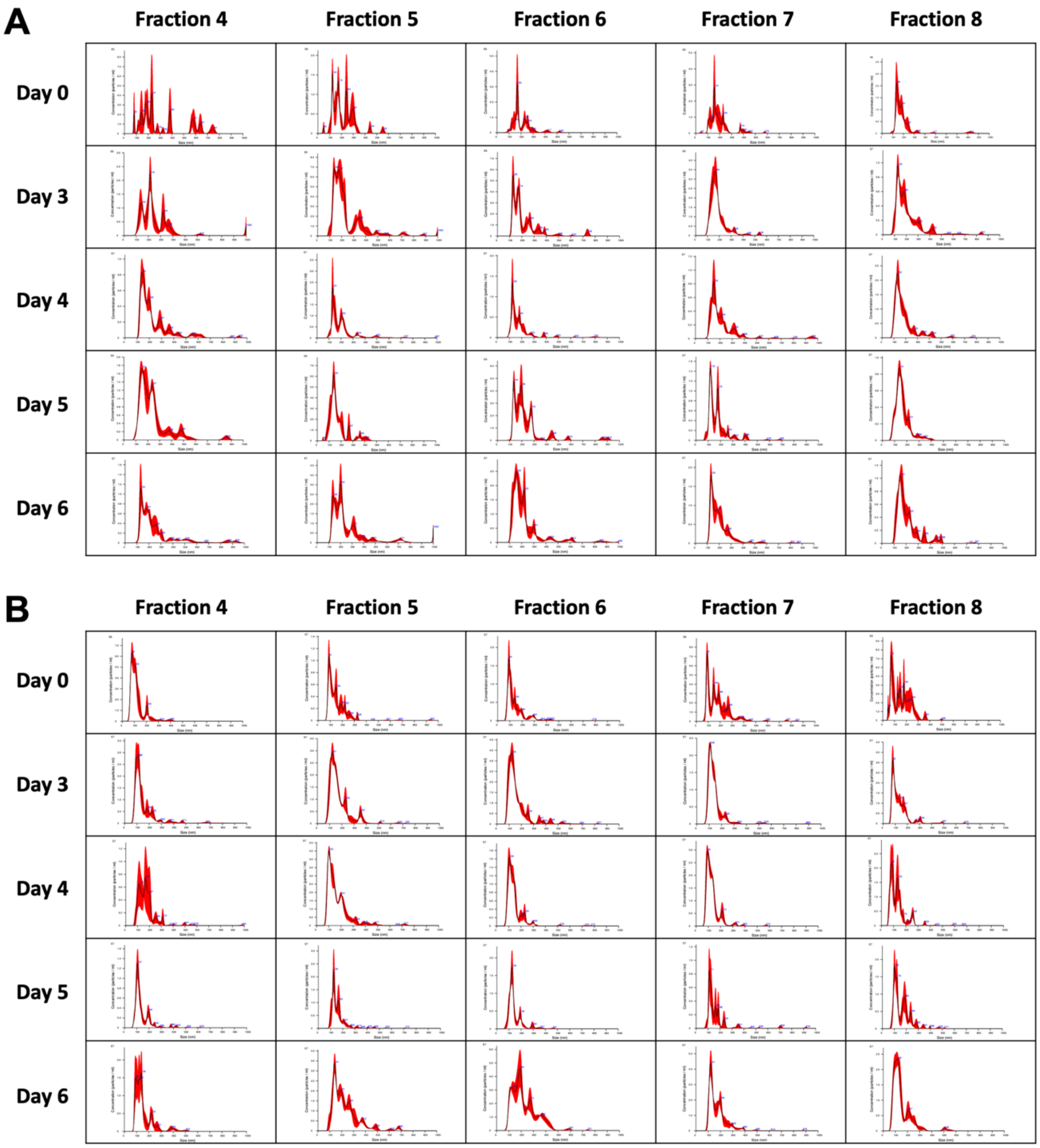


**Supplementary Figure 7. NTA summaries of SEC fraction isolations of serum-derived EVs from EBOV-infected rhesus macaques.** Graphical representation of particle distributions (concentration of estimated sizes) among fractions 4-8 eluted from SEC column loaded with serum from **(A)** NHP 2401 and **(B)** NHP 2441 on day 0 (pre-infection) and days 3, 4, 5, and 6 post-infection, as generated by the NTA software on the NanoSight NS500 platform. Graphs represent mean ± SEM (red) of 3 separate NanoSight videos recorded per fraction (*n* = 1).

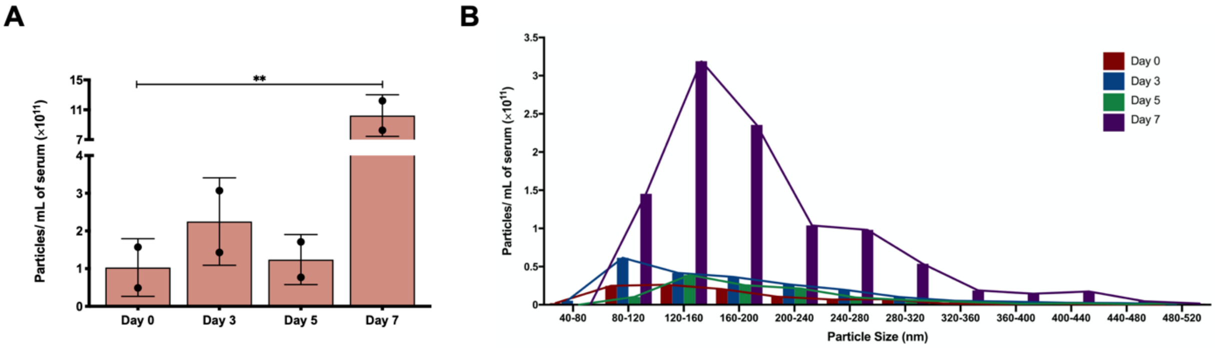


**Supplementary Figure 8. Concentration of all particles in circulation of EBOV-infected rhesus macaques peaks at the terminal stage of infection. (A)** Total particle concentrations in unmanipulated serum from NHP 2401 and 2441 on day 0 (pre-infection) and days 3, 5, and 7 post-infection, as measured by nanoparticle tracking analysis. Bars represent mean ± SD ($n$ = 2, repeated measures one-way ANOVA with Dunnett's correction for multiple comparisons, $**p < 0.01$). Statistical significance is measured against Day 0. **(B)** Histogram of unmanipulated serum particle size distributions (40-80 nm, 80-120 nm, 120-160 nm, 160-200 nm, 200-240 nm, 240-280 nm, 280-320 nm, 320-360 nm, 360-400 nm, 400-440 nm, 440-480 nm, 480-520 nm) according to respective concentrations in samples collected on day 0 (pre-infection) and days 3, 5, and 7 post-infection in NHP 2401 and 2441. Bars represent mean of serum analyses from two macaques per time point ($n$ = 2).

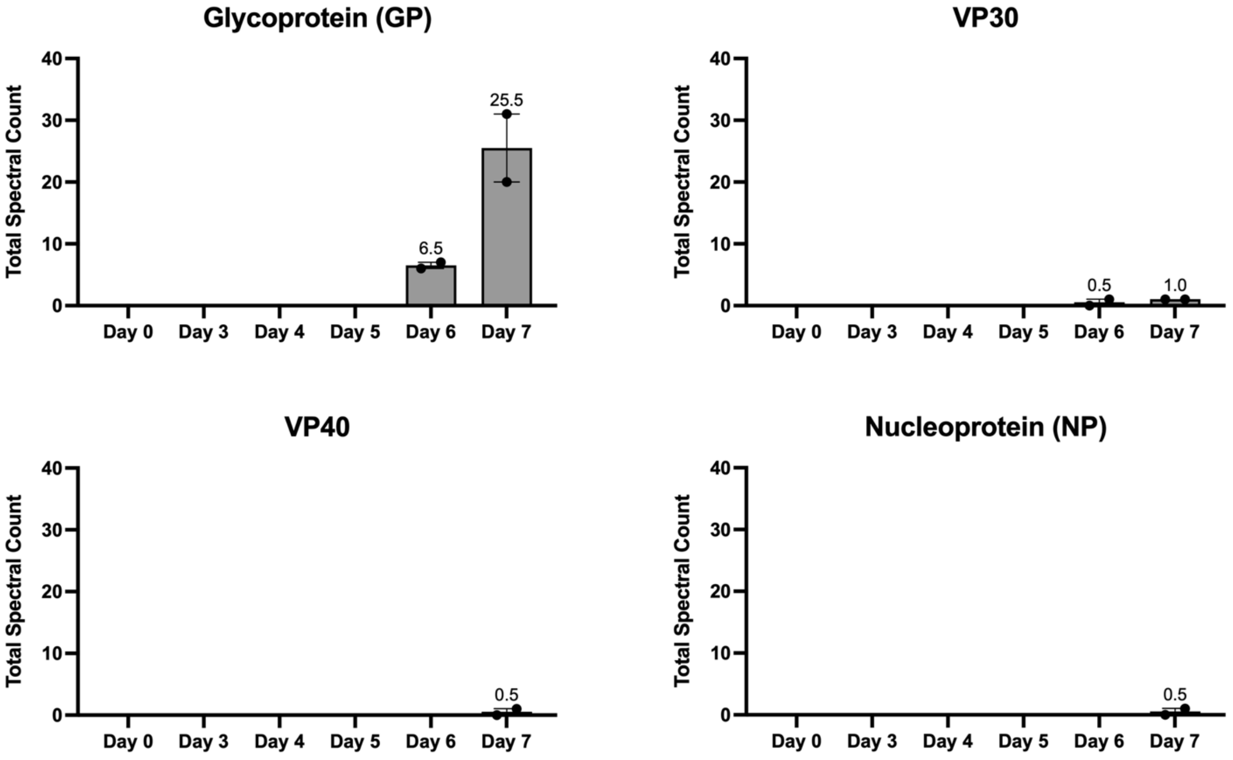


**Supplementary Figure 9. Quantitative proteomic profile of EBOV proteins identified in serum-derived EVs from EBOV-infected rhesus macaques before, during, and at terminal stage of infection.** LC-MS/MS proteomic files were generated using Mascot software and mapped against the UniProt_Viruses database. Protein identifications were accepted if they could be established at greater than 95.0% probability and contained at least 1 identified peptide. Proteins mapped to the *Zaire ebolavirus* (OX ID: 186538) were identified as GP (A0A0A0UG51_9MONO), VP30 (A0A0A7P6S7_9MONO), VP40 (A0A0H3W397_9MONO) and NP (A0A068J9B9_9MONO). Values are expressed as total spectral counts, and labels indicate the mean spectral count for each group.

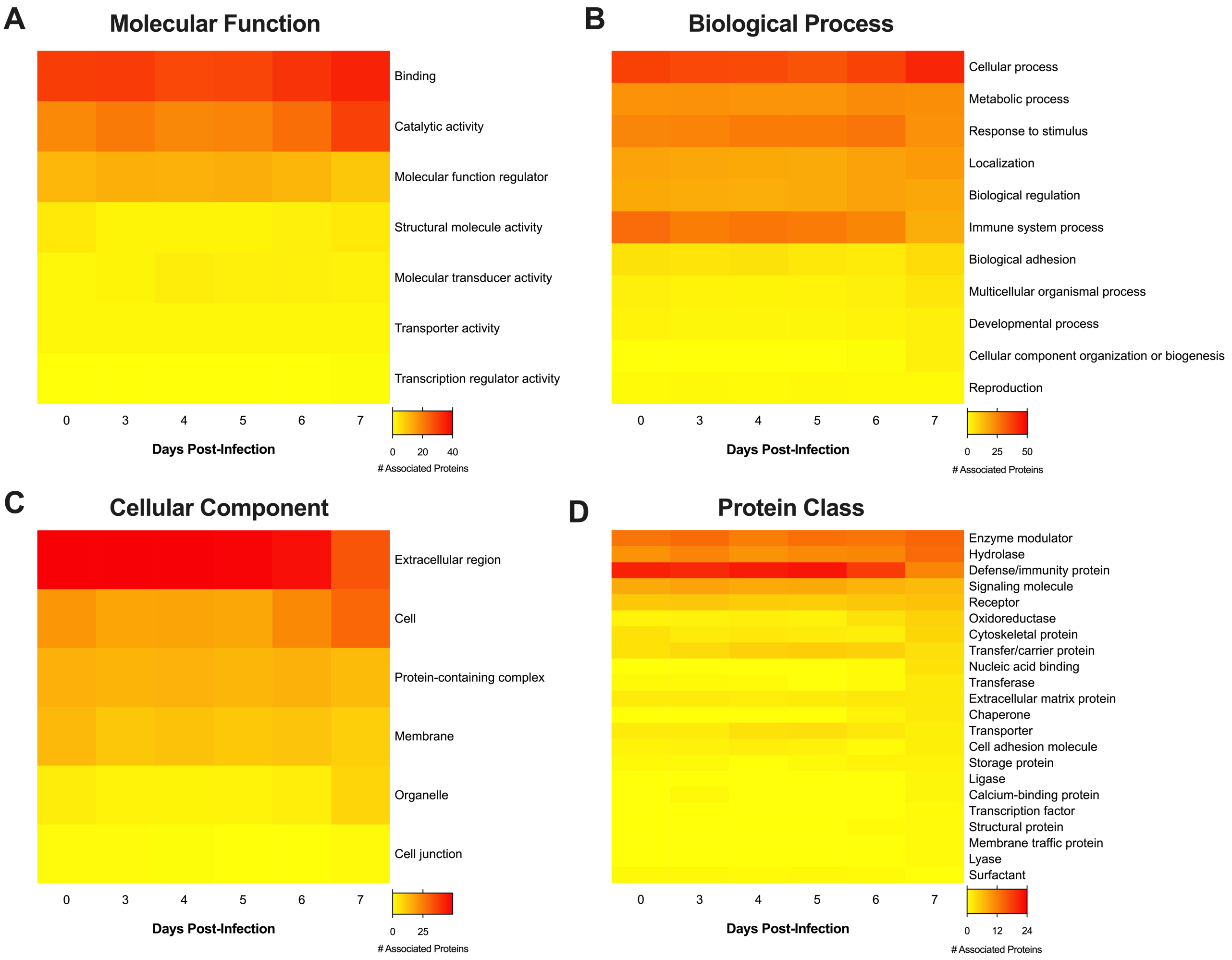


**Supplementary Figure 10. Comparison of proteins involved in GO pathways present in serum-derived EVs from EBOV-infected rhesus macaques pre- and post-infection.** Proteins from EVs isolated from serum collected on days 0, 3, 4, 5, 6 and 7 post-infection were classified using Gene Ontology analysis (Panther Database) according to **(A)** molecular function, **(B)** biological process, **(C)** cellular component, and **(D)** protein class. In all heatmaps, expression is indicated in a colour scale ranging from yellow (low) to red (high)
